# Topological Characterization of Human and Mouse m^5^C Epitranscriptome Revealed by Bisulfite Sequencing

**DOI:** 10.1155/2018/1351964

**Published:** 2018-06-13

**Authors:** Zhen Wei, Subbarayalu Panneerdoss, Santosh Timilsina, Jingting Zhu, Tabrez A. Mohammad, Zhi-Liang Lu, João Pedro de Magalhães, Yidong Chen, Rong Rong, Yufei Huang, Manjeet K. Rao, Jia Meng

**Affiliations:** ^1^Department of Biological Sciences, Xi'an Jiaotong-Liverpool University, Suzhou, Jiangsu 215123, China; ^2^Integrative Genomics of Ageing Group, Institute of Ageing and Chronic Disease, University of Liverpool, L7 8TX Liverpool, UK; ^3^Greehey Children's Cancer Research Institute, University of Texas Health Science Center at San Antonio, San Antonio, TX 78229, USA; ^4^Department of Cellular Structural Biology, University of Texas Health Science Center at San Antonio, San Antonio, TX 78229, USA; ^5^Institute of Integrative Biology, University of Liverpool, L7 8TX Liverpool, UK; ^6^Department of Epidemiology and Biostatistics, University of Texas Health Science Center at San Antonio, San Antonio, TX 78229, USA; ^7^Department of Electrical and Computer Engineering, University of Texas at San Antonio, San Antonio, TX 78249, USA

## Abstract

**Background:**

Compared with the well-studied 5-methylcytosine (m^5^C) in DNA, the role and topology of epitranscriptome m^5^C remain insufficiently characterized.

**Results:**

Through analyzing transcriptome-wide m^5^C distribution in human and mouse, we show that the m^5^C modification is significantly enriched at 5′ untranslated regions (5′UTRs) of mRNA in human and mouse. With a comparative analysis of the mRNA and DNA methylome, we demonstrate that, like DNA methylation, transcriptome m^5^C methylation exhibits a strong clustering effect. Surprisingly, an inverse correlation between mRNA and DNA m^5^C methylation is observed at CpG sites. Further analysis reveals that RNA m^5^C methylation level is positively correlated with both RNA expression and RNA half-life. We also observed that the methylation level of mitochondrial RNAs is significantly higher than RNAs transcribed from the nuclear genome.

**Conclusions:**

This study provides an in-depth topological characterization of transcriptome-wide m^5^C modification by associating RNA m^5^C methylation patterns with transcriptional expression, DNA methylations, RNA stabilities, and mitochondrial genome.

## 1. Introduction

DNA methylation is a well-established and extensively studied epigenetic phenomenon [1–4]. In contrast, mRNA methylation is still relatively an uncharted territory [5]. Although the presence of the chemical modifications to tRNA has been established in the 1970s [6–8], little is known about the epigenetic modifications to mRNA and other noncoding RNAs. Even less was known about their abundance, role, and mode of regulation until recently when several studies showed that *N* ^6^-methyladenosine (m^6^A) is the most abundant messenger RNA (mRNA) modification in eukaryotes [9], and suggested to regulate a number of biological processes including translation efficiency [10], circadian clock [11], microRNA processing [12], RNA-protein interaction [13], RNA stability [14], heat shock response [15], and differentiation [16].

Compared to m^6^A, even little is known about the abundance and role of transcriptome 5-methylcytosine (m^5^C) modification. Existing studies of m^5^C in cellular RNA have been largely confined to rRNA and tRNA [17]. For example, RNA m^5^C modification in plant rRNA and tRNA is reported to be conserved [18] and is shown to affect the stability of synthetic RNA [19, 20]. In the mammalian system, cytosine-5 methylation in tRNA has been shown to regulate Mg^2+^ binding, anticodon stem-loop conformation, and secondary structure stabilization [21, 22]. In addition, m^5^C in tRNAs is reported to regulate protein translation in stress response, tissue differentiation, and neurodevelopment disorders [23–29]. In rRNA, m^5^C is shown to regulate the translation process [30]. A recent study also showed that hm^5^C, the intermediate of RNA m^5^C demethylation, is enriched in poly(A)-tailed RNA and the coding sequences of the mRNA transcript, and it is associated with brain development and the active transcription of mRNA [11].

A recent advancement of the RNA bisulfite-sequencing (BS-Seq) technique [31–34] has enabled the transcriptome-wide m^5^C profiling at single-base resolution and confirmed its widespread existence in the human transcriptome [34, 35]. Intriguing differences with respect to the degree of transcriptome m^5^C methylation, functional classification, and position bias were reported with this technique [36], and it was recently shown that transcriptome m^5^C promotes mRNA export through methyltransferase NSUN2 and reader ALYREF [37].

It is observed that m^5^C modification may account for 20% of the total internal methylations on poly(A) RNA in the BHK21 cell line [38, 39]. However, it is not clear whether the transcriptome m^5^C modification is differentially enriched in different cell types, and the topological relationship between RNA methylation and DNA methylation under the same cell lines has not been investigated.

In this study, using the BS-Seq approach, we identified transcriptome-wide mRNA m^5^C methylome in mouse and human cells. Our results revealed that transcriptome m^5^C is enriched and conserved at the 5′UTRs of target transcripts in both human and mouse cells. Interestingly, under all the examined cell lines, we observed a negative correlation of the methylation patterns between RNA m^5^C methylation and DNA m^5^C under the CpG context, and the RNA m^5^C methylations are enriched on mitochondrial transcriptome.

## 2. Material and Methods

### 2.1. Sample Preparation and RNA Bisulfite Sequencing

MCF10A normal mammary epithelial cells and MDA-MB-468 breast cancer cells were obtained from ATCC. MCF10A cells were cultured and maintained in DMEM/F12 (Life Technologies, USA) supplemented with 5% horse serum, EGF (20 ng/ml), hydrocortisone (0.5 *μ*g/ml), insulin (10 *μ*g/ml), and anti-anti (Life Technologies, USA). Likewise, MDA-MB-468 cells were cultured in RPMI (Life Technologies, USA) supplemented with 10% FBS and anti-anti (Life Technologies, USA). For BS-Seq, total RNA was isolated from MCF10A and MDA-MB-468 cells and enriched for poly(A)+ RNA using poly(A) selection kits. The purified RNA is subjected to sodium bisulfite treatment at 60 degrees for 8 hours. The bisulfite-treated RNA was then reverse transcribed and subjected to deep sequencing using the Illumina RNA-Seq protocol. The data has been deposited under Gene Expression Ominous (GEO) with Accession Number GSE84230. To replenish the transcriptome BS-Seq data of the aforementioned human samples (MCF10A and MDA-MB-468), additional datasets are obtained from public resources, including DNA BS-Seq data from MCF10A (GEO GSM659628) [40], transcriptome m^5^C methylation data from mouse embryo stem cells (ESCs) and mouse whole brain profiled by RNA BS-Seq (GEO GSE83432) [36], and mouse ESC DNA methylation data (GSM1873374) [7, 41].

### 2.2. Quality Control and Alignment of BS-Seq Data

The FASTQ files from BS-Seq samples are trimmed with Trim Galore [42], it removes low-quality 3′ ends with a Phred score threshold of 20, and it can remove potential adaptor contamination. Then, the reads are aligned to the reference genomes of mouse and human (mm10 and hg19) with MeRanGs in MeRanTK [43]. The methylation is called using MeRanCall, and regions of the 5′ ends and 3′ ends of the reads are ignored based on the threshold cutoff suggested by the M-bias plot generated by MeRanGs. The minimum read coverage for the methylation report was set at 10, and the minimum read base quality (Phred score) for methylation call is filtered at 30. The maximum read duplication level is set at 10 to prevent the PCR artefacts; the minimum nonconversion rate to report is set at 0 to include the nonmethylated sites as background control for further analysis.

For DNA bisulfite samples, the trimmed reads are aligned using Bismark under the following alignment setting: --score_min L,0,-0.6. The SAM files are filtered by Samtools using -F 1540 and -q 30 to remove reads that are duplicated and quality scores that are lower than 30. The methylation status of genome-wide cytosine sites is reported from the filtered SAM files with the Bismark methylation extractor using the following argument: --cytosine report. Also, the conversion rate biased ends are also ignored during methylation call based on the M-bias plots. The minimum read coverage was filtered at 10 as well.

### 2.3. Filtering False Positive m^5^C Sites due to RNA Secondary Structure

It is known that secondary structures on RNAs prohibit bisulfite conversion and thus can result in false positive detection of transcriptome m^5^C sites. As shown in Figure S1, the detected m^5^C sites from MeRanTK are enriched with double-stranded regions of RNA, which are likely to be false positive errors due to a secondary structure. For this reason, an R package rBS2ndStructure was created to facilitate the elimination of the false positive methylation calls due to RNA secondary structures. Specifically, the RNA secondary structure is predicted with RNAfold from the Vienna RNA package [44] as it was performed by Amort et al. [36]. The transcriptome-wide full-length transcripts are extracted from UCSC gene annotation for both mm10 and hg19. Then, the double-stranded structures are predicted with the MEA method under alpha = 0.1. The folding temperature is set at 70 degrees, and the maximum pairing distance is set at 150 bp. For the mitochondrial chromosome and transcripts longer than 8000 bp, the structures are predicted using sliding windows of 2000 bp and step size of 1000 bp. For both the RNA and the DNA methylation reports, the methylation sites overlapped with the predicted regions of secondary structures are filtered. Due to the lack of computational resources to predict structures on large intronic sequences, the cytosine sites that do not locate on the exons of known transcripts or the mitochondrial chromosome are filtered. The resulting methylation reports are then analyzed under the R environment using primarily GenomicFeatures [45], Guitar [46], and ggplot2 [47] packages.

The rBS2ndStructure package is publically available at Github (https://github.com/ZhenWei10/rBS2ndStructure) with precomputed RNA secondary structures of genome assembly mm10 and hg19 for convenient processing of RNA BS-Seq result.

### 2.4. Quantitative Analysis of Methylation Status

The methylation ratio (mRatio) of a specific cytosine site is calculated by

(1) $$\begin{array}{c} Methylation\;ratio=\frac{\#\text{of }unconverted\;Cs}{\#\text{of }unconverted\;Cs+\#\text{of }converted\;Cs}, \end{array}$$

where “# of unconverted Cs” and “# of converted Cs” indicates the count of methylated (unconverted) Cs and unmodified Cs (converted Cs) at a specific cytosine site, respectively. The methylation rate is conceptually similar to the well-adapted concept of “beta value” in DNA methylation analysis [48], which indicates the percentage of methylated Cs among all Cs. Also, it is not difficult to show that

(2) $$\begin{array}{c} Methylation\;ratio=\frac{\#\text{of }unconverted\;C}{\#\text{of }unconverted\;C+\#\text{of }converted\;C}=1-\frac{\#\text{of }converted\;C}{\#\text{of }unconverted\;C+\#\text{of }converted\;C}=1-convertion\;rate, \end{array}$$

where the conversion rate has been previously defined in [35] and a smaller value suggests a higher percentage of RNA m^5^C methylation.

To differentiate a set of statistically significantly methylated cytosine sites against potential technical randomness due to incomplete bisulfite conversion, the *p* values for the methylation state of both the DNA and RNA methylation are calculated by Fisher's exact test against the background conversion odds after the filtering of the sites mapped to introns and secondary structures. The adjusted *p* values (FDR) are then adjusted by the Benjamin & Hochberg method. The positive methylation states were decided when FDR < 0.05.

For the mouse samples containing 3 biological replicates, the methylated sites are judged as FDR < 0.05 among all 3 replicates. For other insignificant methylated sites to be kept in the analysis, the sites should be reproduced 3 times with coverage > 10. The converted reads and nonconverted reads are added on each site when combining the biological replicates.

The background bisulfite nonconversion rate is 2.75%, 2.74%, 1.18%, and 0.81% for MCF10A, MDA468, mouse ESC, and mouse brain samples, respectively (taking the average for samples with more than one biological replicate). The difference among nonconversion rates might be due to the biological difference of cell lines, batch variation, and different BS-Seq protocols.

### 2.5. Differential Methylation Analysis

The odds ratio (OR) or methylation fold change from differential analysis is defined as

(3) $$\begin{array}{c} Odds\;ratio\;from\;differential\;methylation=\frac{\left(\#\text{of }unconverted\;Cs\;under\;cond\_1/\#\text{of }converted\;Cs\;under\;cond\_1\right)}{\left(\#\text{of }unconverted\;Cs\;under\;cond\_2/\#\text{of }converted\;Cs\;under\;cond\_2\right)}. \end{array}$$

Odds ratio (or methylation fold change) indicates whether the methylation is enriched under one condition compared with another condition. A value greater than 1 suggests increased methylation level, where as a value less than 1 suggests decreased methylation level. The statistical significance of the odds ratio is evaluated by the QNB method, which tests the homogeneity of association between methylated and unmodified molecules under two experimental conditions with the within-group variability assessed through 4 cross-linked negative binomial distributions [49].

Similar to the odds ratio from differential methylation analysis, the enrichment odds ratio of m^5^C sites within a specific region can be defined as

(4) $$\begin{array}{c} Enrichment\;odds\;ratio=\frac{\left(\#\text{of }m^{5}C\;sites\;within\;a\;region/\#\text{of }C\;sites\;within\;a\;region\right)}{\left(total\#\text{of }m^{5}C\;sites/total\#\text{of }C\;sites\right)}. \end{array}$$

A value greater than 1 suggests that methylation sites are enriched within the tested region, and the statistical significance of enrichment can be evaluated by Fisher's exact test. Please note that, in this analysis, we used the total number of cytosine sites reported from MeRanTK rather than the total number of all 4 types of nucleotides.

### 2.6. Assessing the Distribution of m^5^C Sites on mRNA

The distribution pattern of m^5^C sites on mRNA is assessed with the Guitar R/Bioconductor [46]. Compared with other software tools and methods, the Guitar package provides an improved resolution by relying on only the mRNA transcripts that simultaneously have sufficient long (more than 100 bp) 5′ UTRs, CDSs, and 3′ UTRs. For instance, transcripts without annotated 5′UTRs will be excluded from the analysis. Additionally, Guitar does not rely on only the primary transcript (often defined as the longest transcript among all isoforms in practice) when solving an ambiguous association between a m^5^C site and the isoform transcripts of a gene; instead, all ambiguous associations are considered with the weight of association evenly divided. For example, if a single m^5^C site locates on the 3′UTR of a transcript and CDS of another isoform transcript of that gene, it is counted as if half of the m^5^C site is located on the 3′ UTR and the other half located on 5′ UTR. In this way, the isoform information is largely retained. To our knowledge, the Guitar package should provide the most accurate assessment of a transcriptomic distribution pattern.

### 2.7. Differential Expression Analysis

Differential expression analysis was performed with the DESeq2 package [50] and the aligned RNA BS-Seq data.

### 2.8. Cell Culture and Viral Infection

Jurkat T lymphocytes were maintained in RPMI 1640 medium (Hyclone) supplemented with 5% (*v*/*v*) FBS (Gibco) and 100 U/ml penicillin/streptomycin (Hyclone). For infection, Jurkat cells were infected with known amounts (3 × 108 genome copies per 2 × 105 cells) of SRV for 18 hours at 37°C, followed by washing three times with PBS (Hyclone). Infected cells were incubated in completed culture medium for the indicated time. Successful infection was identified as the appearance of cytopathic effects in infected cells at 8 to 10 days postinfection.

### 2.9. Reverse Transcription and Real-Time PCR

SRV genome in culture medium was extracted by viral RNA extraction kit (TIANGEN) and reverse transcripted into cDNA by a reverse transcriptase PCR kit (TaKaRa). Cellular genome was extracted by a TIANamp Genomic DNA Kit (TaKaRa). Real-time PCR was performed in a 7500 Fast Real-Time PCR System (Applied Biosystems) by using a Premix Ex Taq (Probe qPCR) kit (TaKaRa). SRV genome positive control, primers, and probe, as well as GAPDH primers and probe were kindly provided by VRL China Ltd. [51].

### 2.10. Immunofluorescence Assay

Cells were seeded on poly-L-lysine (Sigma) coated slides, fixed with 4% paraformaldehyde for 15 minutes, permeabilized with precold pure methanol for 20 min at −20°C, and blocked with 5% BSA for 1 hour. Cells were then stained with the serum from an SRV-infected monkey (1 : 25 diluted in blocking buffer) overnight and visualized with DyLight™ 488-Labeled Anti-Human antibody (KPL). Cells were counterstained with Hoechst (Life Technologies) for 10 minutes and mounted on microscopy slides. Samples were imaged with a ZEISS LSM 880 Confocal Laser Scanning Microscope.

## 3. Results

### 3.1. Overview of mRNA m^5^C Methylome Revealed by BS-Seq

**Figure 1:**
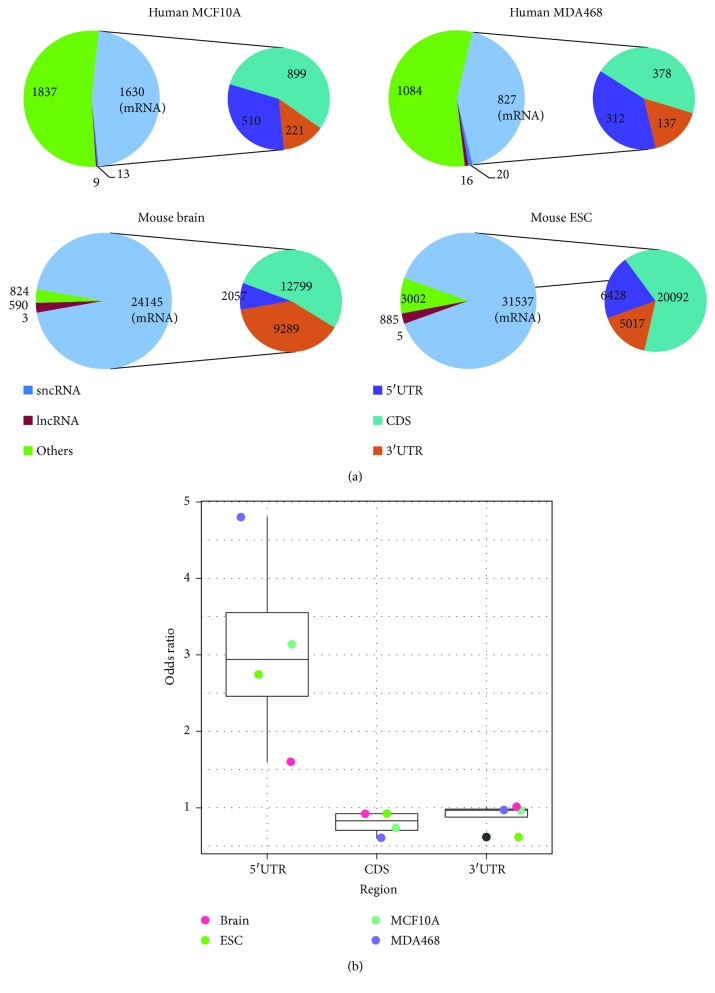
Distribution of transcriptome m^5^C modification sites in human and mouse. (a) The pie chart shows transcriptome-wide distribution of m^5^C sites in MCF10A, MDA-MB-468, mouse embryonic stem cell (ESC), and whole brain. The majority of the identified m^5^C sites are located on mRNAs. (b) Graph showing status of m^5^C frequency in different regions of mRNA. The result indicates that detected cytosine sites are consistently enriched at the 5′UTR on mRNA compared with the CDS and 3′UTR.

After successful processing of the RNA BS-Seq datasets, a total of 3440 (0.40%), 1915 (0.29%), 35,246 (0.757%), and 25,301 (0.50%) RNA cytosine sites were identified as m^5^C methylation (FDR < 0.05) sites in MCF10A, MDA-MB-468, mouse embryonic stem cell (ESC), and mouse whole brain, respectively. The overall transcriptome m^5^C methylation level was much lower than the DNA m^5^C methylation level (Figure S2). Importantly, we found that m^5^C was widespread in different RNA families, where more than 50% of them were located on mRNA (Figure 1(a)). In MCF10A cells, 7131 protein-coding genes had sites reported after the filtering, of which 225 (3.15%) mRNAs contained m^5^C sites. In MDA-MB-468 cells, 6320 protein-coding genes had reads aligned, of which 128 (2.06%) contained m^5^C sites. In ESC and brain samples, the methylation status was available for 11,325 and 13,108 protein-coding genes, of which 3579 (31.6%) and 3065 (23.4%) contained m^5^C sites. The difference in number of m^5^C sites between different conditions is mostly due to different sequencing depth.

### 3.2. mRNA m^5^C Is Enriched in 5′UTRs of Human and Mouse

To study the spatial organization of m^5^C sites in the transcriptome, we first analyzed the relative enrichment (see Materials and Methods for more details) of m^5^C sites on different types of RNA and at different regions (shown in Figure 1(b)) by compensating for the cytosine sites that do not carry m^5^C modification. Our results showed that m^5^C sites were consistently and significantly enriched at 5′UTRs in human and mouse with enrichment odds ratio of 3.138, 4.802, 2.744, and 1.601 (please see Table S1 for more details). The similar topology was already reported by previous studies [35, 36], and our observation further confirmed their conclusions. Also, we did observe a slight enrichment of m^5^C sites in 3′UTR in mouse brain (enrichment odds ratio = 1.013 and 1.19*E* − 02), which is also reported in the study of Amort et al. [36]. 3′UTR enrichment was not observed in the other samples (odds ratio = 0.964, 0.971, and 0.617).

**Figure 2:**
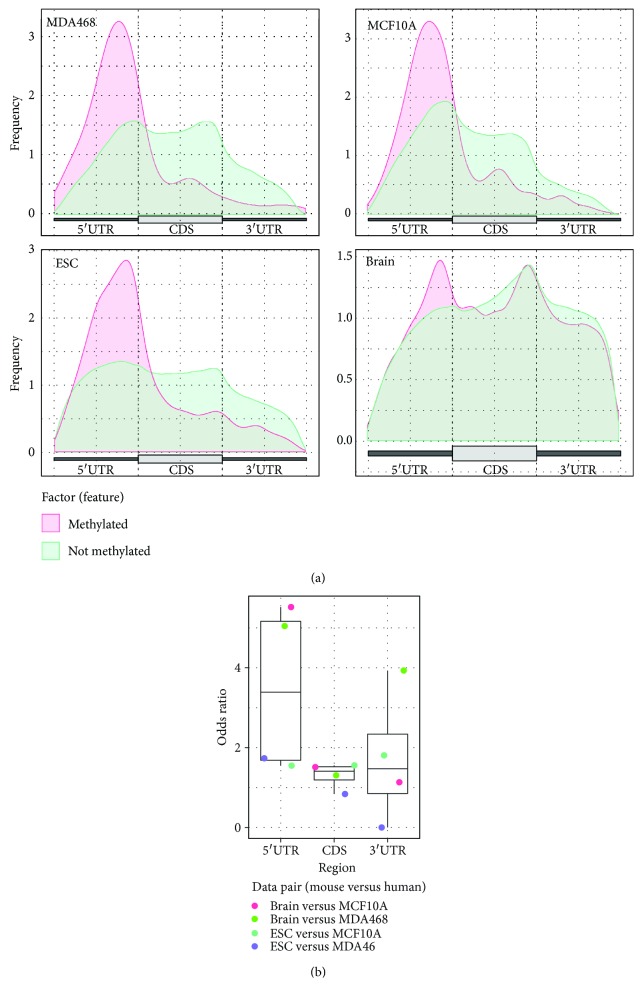
Conservation of m^5^C in different mRNA regions. (b) Graph showing the status of m^5^C frequency in different regions of the transcripts. We divided all the detected cytosine sites into 2 groups based on whether it is methylated. The result indicates that cytosine sites with significant methylation levels are consistently enriched at the 5′UTRs and near the start codon in all 4 samples. (b) A correlated methylation pattern is observed on 5′UTRs between different cell lines/tissues in human and mouse. The conserved cytosine residuals were retrieved with liftOver utility (http://genome.ucsc.edu/cgi-bin/hgLiftOver), and the correlation analysis is performed with Fisher's exact test. It is important to note that, although we failed to observe a correlated m^5^C methylation pattern on CDSs and 3′UTRs of mRNA, it is possible that such pattern may emerge on strictly matched cell lines/tissues.

To further substantiate these findings, we plotted the distribution of the methylated and unmethylated cytosine sites located on mRNAs with the Guitar package [46]. In order to improve the resolution of this analysis and differentiate the distribution of m^5^C sites on usually short 5′UTRs, only the mRNAs with a 5′UTR longer than 100 bp are used. As shown in Figure 2(a), the methylated cytosine sites were consistently enriched at 5′UTRs across all 4 samples when compared to unmethylated groups. Interestingly, this trend is also supported by the cytosine methylation sites reported by Squires et al. [35], and there is no significant enrichment of m^5^C sites observed in 3′UTR when all cytosine methylated sites were used as background (Figure S3).

When we further compared the methylation status of the conserved loci in human and mouse between different cell lines/tissues, we observed that, although the cell types/tissues we used were not strictly matched, a strong correlated methylation pattern was observed on the 5′UTR region (Figure 2(b) and Table S2). However, unlike the 5′UTR, the correlated pattern of m^5^C sites were not consistently observed in CDSs or 3′UTRs in our study; the observed heterogeneity of the m^5^C methylome in different transcript regions suggests that the m^5^C mapped to the 5′UTR of the transcripts are more likely to be functionally important.

### 3.3. m^5^C Site Exists under Different Nucleotide Contexts

**Figure 3:**
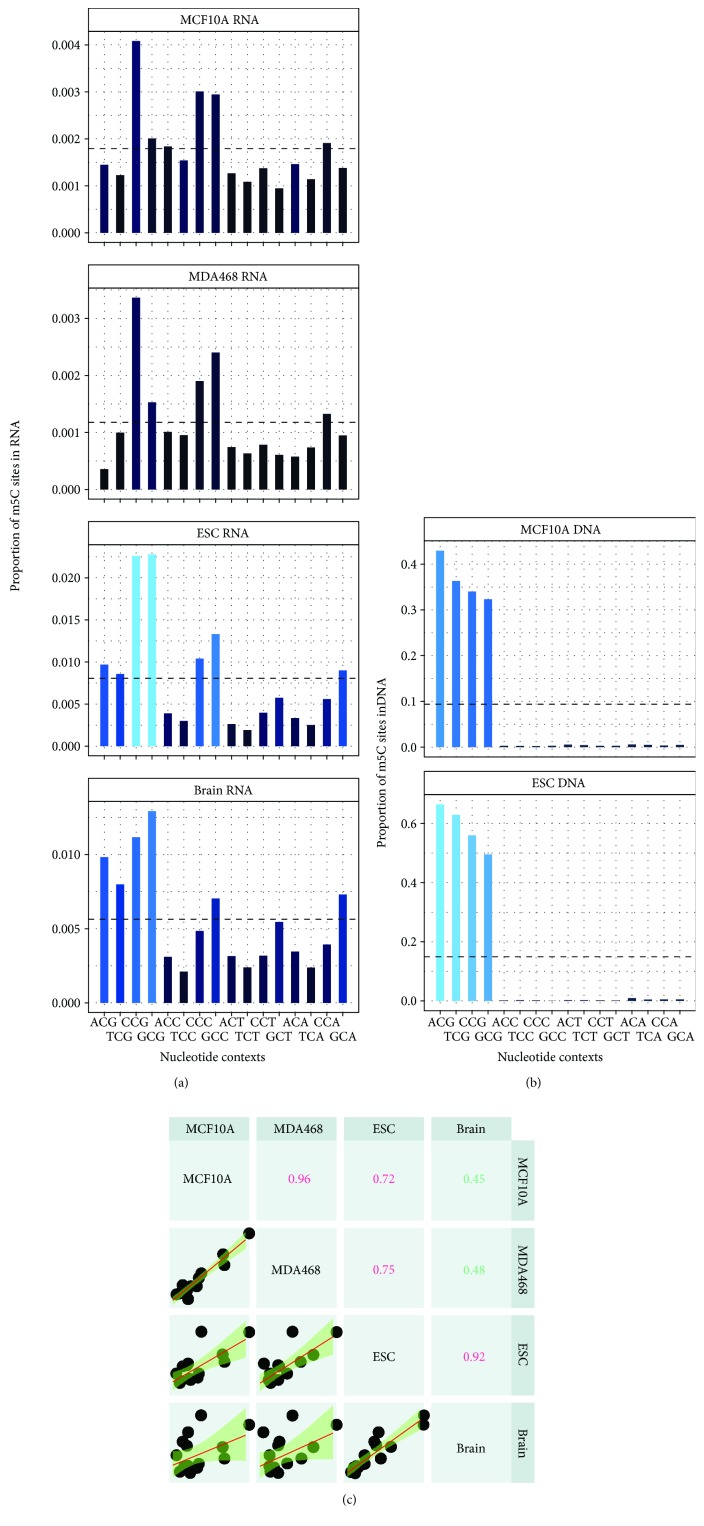
Comparative distributions of mRNA and DNA m^5^C methylation. (a) Bar graph shows the proportion of mRNA m^5^C sites under different combinations of C-centered trinucleotides in mouse and human cells. The dotted line shows the average percentage of methylation under all trinucleotide contexts within the entire transcriptome. We observed that RNA m^5^C occurs under all trinucleotide contexts and is slightly enriched in sequences containing CCG, GCG, GCC, GCU, and GCA. (b) Bar graph showing proportion of DNA m^5^C sites in mouse and human cells. DNA cytosine sites were enriched exclusively in sequences containing CG dinucleotides (ACG, CCG, CCG, and TCG). (c) The coefficient of correlation between RNA methylation and trinucleotide sequences was found to be consistent between samples from the same species (Pearson correlation = 0.96 for human and 0.92 for mouse) and also between human and mouse cells (Pearson correlation = 0.72, 0.75, 0.45, and 0.48).

Because RNA methyltransferase Dnmt2 shares strong sequence homology to all DNA DNMT methyltransferases [52], we reason that exploring the relationship between transcriptome and DNA m^5^C methylation profiles may unravel interesting interplay between the two kinds of reversible chemical modifications. In mammalian cells, DNA methylation occurs mainly at CG dinucleotides (including ACG, CCG, TCG, and GCG, see Figure 3(b)). To study whether, like DNA methylation, transcriptome m^5^C methylation also occurs at the similar position, we analyzed methylated cytosine in the transcriptome. For this purpose, we examined all the possible C-centered trinucleotide combinations. Unlike DNA, transcriptome m^5^C occurs at all C-centered trinucleotides (Figure 3(a)) and was observed to be specifically enriched at GCA, ACG, CCG, GCG, CCC, and GCC. These results were found to be consistent within the same species (Pearson correlation = 0.96 and 0.92, Figure 3(c)) and between different species (Pearson correlation = 0.72, 0.75, 0.45, and 0.48, Figure 3(c)).

### 3.4. Negative Correlation in Methylation Level Is Observed between mRNA and the Corresponding Exonic Region of DNA

**Figure 4:**
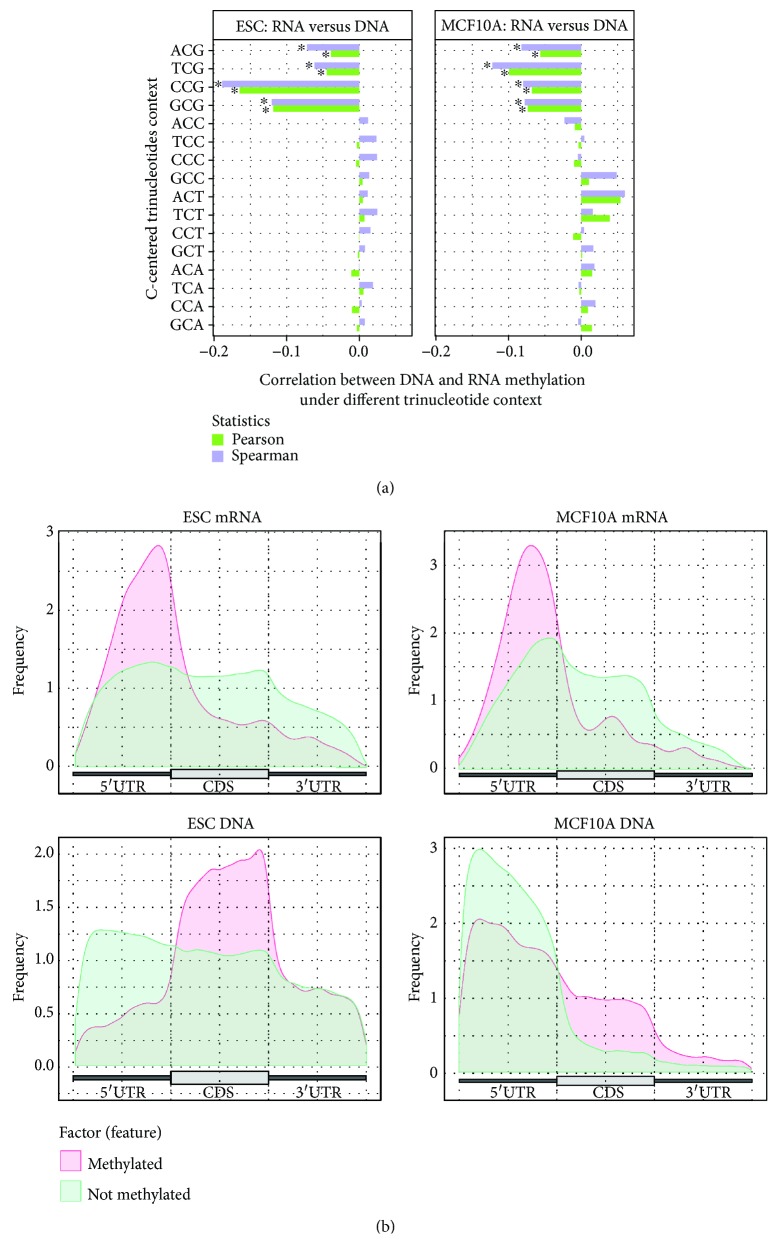
The methylation ratio of corresponding m^5^C DNA and mRNA CpG islands shows negative correlation. (a) Negative correlation is observed between DNA and mRNA methylation ratio consistently under all four CG containing trinucleotides (ACG, TCG, CCG, and GCG) in both human and mouse, that is, if a specific CG dinucleotide in DNA is methylated, the corresponding dinucleotide in mRNA is significantly less likely to be methylated. ^∗^The top 4 nucleotide contexts under which the strongest correlation between DNA and RNA methylation level exists. (b) Comparative distributions of m^5^C methylated CG sites in DNA and RNA show an enrichment of sites with a high methylation ratio in mRNA 5′UTR as opposed to an enrichment of low-methylation-ratio sites in DNA 5′UTR. The pattern is consistent in both the human MCF10A cell line and mouse embryo stem cells.

We next examined whether there exists any correlation between m^5^C methylated/nonmethylated (m^5^C methylation ratio) in the transcriptome and corresponding DNA exonic regions at each C-centered trinucleotide sites. Because DNA methylation occurs mainly at CG dinucleotides, as expected, we observed no strong correlation at non-CG trinucleotides. However, we observed significant negative correlation in methylation ratios between RNA and DNA at all four CG-containing trinucleotides. As a higher percentage of m^5^C in mRNA is detected, the corresponding DNA exonic CG dinucleotide was less likely to be methylated (Figure 4(a)). Next, we grouped m^5^C methylated at all CG sites according to their methylation ratio (methylated and unmethylated) and investigated their distributions in mRNA and the corresponding exonic regions of DNA. Consistent with our previous finding, we observed a significant negative correlation in both human and mouse cells. In particular, 5′UTR in mRNA showed a high methylation ratio, whereas the corresponding DNA region showed a significantly low methylation ratio (Figure 4(b)).

### 3.5. Transcriptome m^5^C Sites Exhibit a Clustering Effect

**Figure 5:**
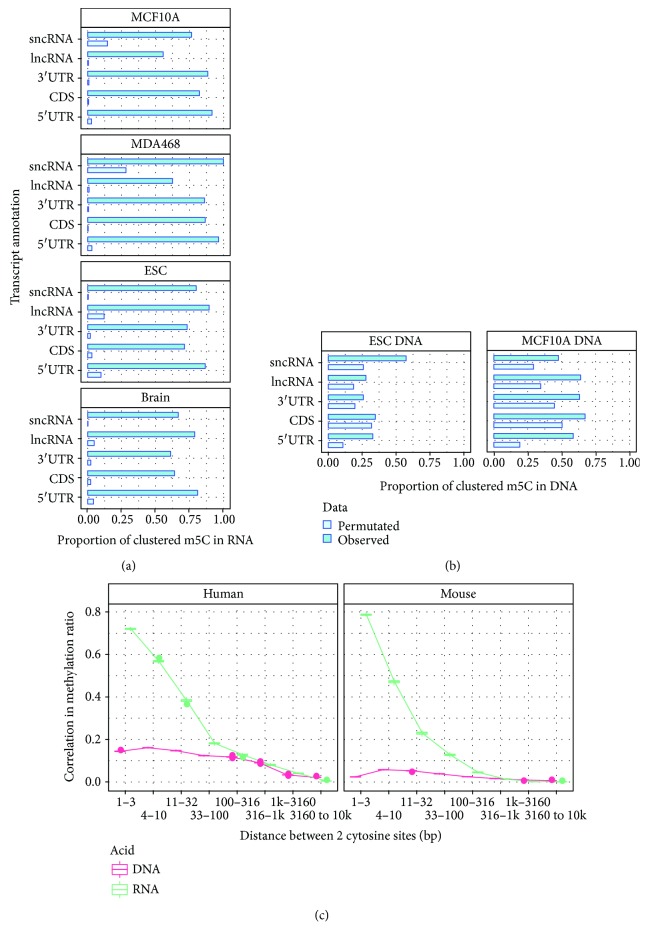
RNA m^5^C modification exhibits a clustering effect. (a) Bar graph shows the proportion of clustered m^5^C sites within 10 nt flanked regions. To evaluate the statistical significance, we generated 1000 permutated results as a comparison with the bars indicating a 99% confidence interval. Using these criteria, m^5^C methylation showed a strong clustering effect consistently on different RNA families and on different regions of mRNA in human and mouse. Around 50% of the m^5^C sites were clustered with each other within a 10 bp region. (b) DNA methylation also exhibited a clustering effect. However, the pattern is not that strong when all nucleotide contexts are considered. (c) Line graph showing correlation between RNA/DNA m^5^C methylation and distance between cytosine sites. RNA m^5^C methylation showed strong correlation with cytosine sites that are immediately close to each other. The clustering effect of DNA methylation is strong when only CpG context is considered (Figure S4).

In DNA methylation, it has been shown that the correlation of methylation rates between two CpG sites is related to the distance (see Figure S4), and the clustering effect can be as high as 0.7 for probes within 200 bp [53]. To address whether the mRNA m^5^C methylation also exhibits a clustering effect, we examined the proportion of m^5^C sites that are within 10 bp distance of other m^5^C sites and compared this proportion with that from 1000 times of random permutation. Our analysis revealed that m^5^C showed an obvious clustering effect in both mRNA and DNA (Figures 5(a) and 5(b). In the ESC cell line, more than 76.7% of the mRNA methylation sites had at least one methylation site mapped within the 10 nt-flanked region, compared with 7.7% of such event by random permutation of methylation states on insignificant methylation sites of the methylated genes. In mouse ESC and brain cells, more than 43.02% and 30.06% of mRNA m^5^C methylation sites existed within the m^5^C-p-m^5^C dimmers, compared with expected rate of 1.02% and 0.77% of such dimmers by the random permutation.

To further elucidate the clustering effect, we calculated the correlation of the methylation ratio between two cytosine sites with a specific distance. To our surprise, mRNA methylation exhibited a stronger clustering effect compared with DNA (Figure 5(c)). In addition, the correlation of the methylation ratio was consistently stronger within 1–3 nt distance as revealed by the higher correlation of the methylation ratio (0.76 in MCF10A and 0.79 in ESC). These results indicated that most CpC dimers are comethylated; the correlation of the methylation ratio can be as high as 0.58 in MCF10A and 0.47 in ESC for cytosine sites with a distance of 4–10 nt. Though the overall clustering effect of DNA methylation was not as strong as mRNA methylation, when only the CpG dinucleotide was considered, DNA methylation exhibited a stronger clustering effect than mRNA methylation (see Figure S4).

### 3.6. Transcriptome m^5^C Is Strongly Enriched in Mitochondrial Transcripts

**Figure 6:**
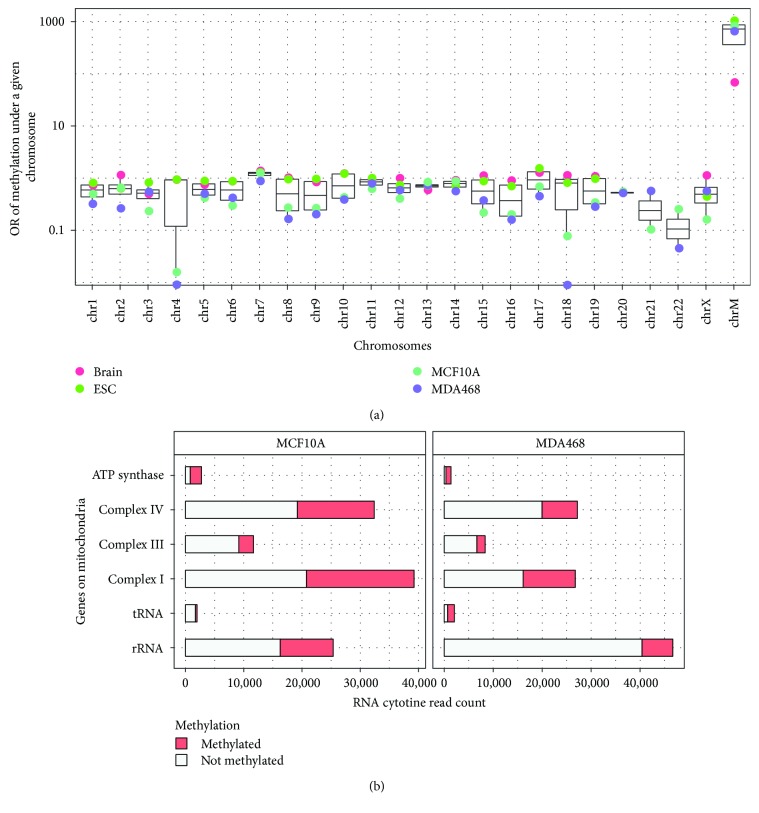
m^5^C is enriched on mRNAs transcribed from mitochondrial DNA. (a) Bar graph depicting m^5^C mRNA methylation sites on different chromosomes. RNAs transcribed from mitochondrial DNA (M) showed drastically increased frequency of m^5^C sites (enrichment odds ratio of 818.42949, 634.72723, 1028.52065, and 67.28553). (b) Bar graph showing the number of methylated cytosine reads stacked with unmodified cytosine reads generated from 6 major classes of mitochondrial genes. The RNA transcripts of all the major genes located on a mitochondrial chromosome were significantly methylated.

To further establish a physiological relevance of m^5^C distribution, we examined the methylation level of RNAs encoded in different chromosomes. Surprisingly, m^5^C modification was strongly enriched in RNAs transcribed specifically from mitochondrial DNA in normal and breast cancer cells as well as in mouse ESC and brain as revealed by enrichment odds ratios of 818.42949, 634.72723, 1028.52065, and 67.28553, respectively. In contrast, the enrichment odds ratios of RNA methylation for transcripts from other chromosomes were found to be roughly the same (Figure 6(a)). The RNA transcripts of all the major genes located on a mitochondrial chromosome were significantly methylated (Figure 6(b)). Previously, it was reported that methyltransferase NSUN5 can regulate mitochondrial gene expression [54], and we speculate that RNA m^5^C may play a more vital regulatory role in mitochondria-related biological processes.

### 3.7. Dysregulation of RNA Methylome in Breast Cancer

**Figure 7:**
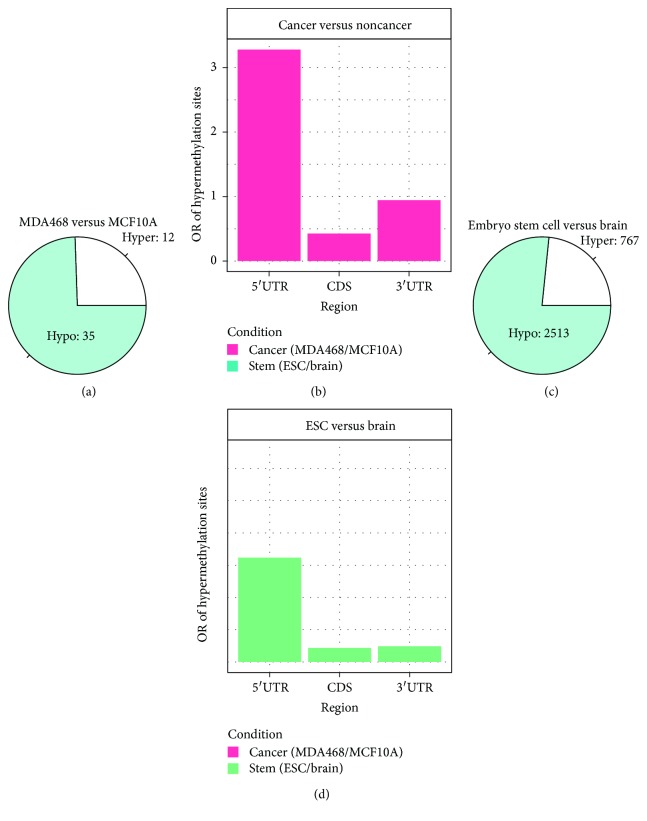
Differential m^5^C mRNA methylation in different tissues. (a) Pie-diagram showing hypo- and hypermethylation in MDA468 when compared to MCF10A. A total of 47 differential methylated genes were identified between the breast cancer (MDA-MB-468) and normal control cell lines (MCF10A) with primary hypomethylation under cancer condition. (b) Bar graph showing odds ratio of hypermethylation sites with respect to all differentially methylated sites on different regions of mRNA. Hypermethylated sites were strongly enriched in 5′UTRs. (c) Pie diagram showing hypermethylation in mouse embryo stem cells when compared to whole brain cells. (d) Bar graph showing odds ratio of hypermethylation sites with respect to all differentially methylated sites on different regions of mRNA in the mouse experiment. Hypermethylated sites were strongly enriched in 5′UTRs.

Comparison of normal (MCF10A) and breast cancer (MDA-BM-468) m^5^C epitranscriptomes identified 162 significant differential methylation sites (DMSs) located on 47 annotated genes at a significance level of 0.05. Among the 47 differentially methylated genes, 35 shows hypomethylation and 12 shows hypermethylation in cancer cells compared with the normal control cell line. The majority of the differential methylation sites show hypomethylation (Excel Sheet S1 and Figure 7(a)), and the m^5^C hypomethylations are mostly located in the CDS and 3′UTR region of mRNA but not in the 5′UTR region (Figure 7(b)). We then investigated whether different m^5^C mRNA methylation levels in normal and breast cancer cells have any functional correlation. We performed functional gene set enrichment analysis on genes containing DMS using the DAVID web server and found that many of the 47 differentially methylated genes are related to important biological functions of cancer, for example, regulation of apoptosis and programmed cell death with RTN4, NME2, CASP14, HSPB1, RPL11, and RPS3 differentially methylated (Excel Sheet S1).

Interestingly, like the difference between the breast cancer cell line MDA-MB-468 and the normal epicelial cell line MCF10A, similar mechanistic mouse stem cells [55] also exhibit dominant hypomethylation in the m^5^C epitranscriptome when compared with mouse brain cells with 2513 genes hypomethylated and 767 genes hypermethylated (Figure 7(c) and Excel Sheet S2). Also similar to the previous case, the hypomethylations are mostly located in the CDS and 3′UTR regions of mRNA, but not in the 5′UTR region (Figure 7(d)). Using DAVID, we found that hypermethylated genes in ESC cells are mostly enriched with the regulation of cell cycle (FZR1, E2F5, BOP1, TRRAP, CDK4, JUNB, etc.), cell death (SIVA1, MCL1, YPEL3, ARF6, UBQLN1, SHF, CIAPIN1, APLP1, GPX1, CASP3, etc.), and mRNA metabolic process (SCAF1, FIP1L1, STRAP, RBM15B, CWC15, XAB2, YBX1, AUH, SF3B2, APLP1, HNRNPL, etc.); the hypomethylated genes are enriched with functions related to ATP synthesis (ATP6V1F, ATP6V1C1, ATP6V0C, ATP6V1A, ATP6V0E, ATP6V1E1, ATP5C1, etc.) and mitochondrial ribosome (MRPL15, MRPL27, MRPL16, MRPL36, MRPL39, MRPL34, DAP3, etc.) (Excel Sheet S2). These results may suggest that the m^5^C methylations are selectively methylate transcripts having functions.

### 3.8. Positive Correlation between m^5^C mRNA Methylation and Expression Changes

In our data, as the gene expression is also estimated from RNA bisulfite-sequencing data, a direct comparison of expression and m^5^C methylation changes may be problematic due to dependent noise. To eliminate the interference of dependent noise between expression and methylation data, the samples are further divided for different purposes. Specifically, the 3 biological replicates are divided into 2 groups, with 1 sample used for the estimation of expression changes and the other 2 samples for estimation of methylation changes. The expression changes and methylation changes are then compared. This procedure was repeated for 3 times using different grouping combinations.

A consistent and significantly positive correlation is observed (0.274, 0.303, and 0.254) between log_2_ expression fold change and log_2_ methylation fold change when comparing mouse embryo stem cells with brain cells (Figure S5), suggesting that increased methylation level is likely to be associated with increased expression level. Although the specific molecular mechanism is not yet clear, the observed positive correlation between RNA m^5^C and RNA expression confirmed our previous observed anticorrelation between DNA and RNA m^5^C methylation (see Figure 4) from a different perspective.

**Figure 8:**
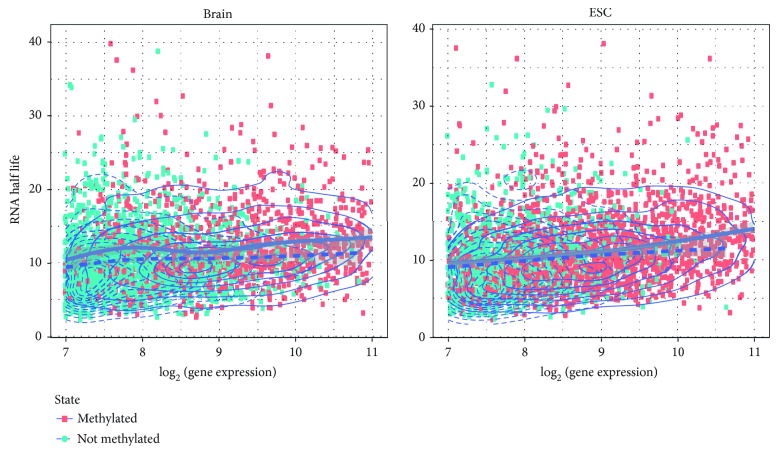
RNA m^5^C status is positively correlated with RNA half-life. In the above figure, each red dot represents a gene that carries m^5^C sites, and each blue dot represents a gene that does not carry an m^5^C site. When comparing the methylated and unmethylated genes of similar expression, the genes that carry an m^5^C site have longer RNA half-life than those that do not carry m^5^C sites. (a) Positive correlation between RNA methylation status and RNA half-life is observed in mouse brain (*p* value = 0.0374, generalized linear model of binomial family). (b) Positive correlation between RNA methylation status and RNA half-life is observed in mouse embryo stem cells (*p* value = 2.23*E* − 12, generalized linear model of binomial family).

To explain the positive correlation between expression and transcriptome m^5^C methylation, we compared the methylation status of all the genes and their half-life, where the half-life of mouse genes were obtained from a previous study [56]. The mRNAs are classified into two groups based on whether they have at least one m^5^C site or not. To exclude the confounding factor (effective size in methylation site calling), a generalized linear model of the binomial family was fitted to the half-life with both expression and methylation information. Our result suggests that there exists a significant positive correlation (*p* value = 2.23*e* − 12) between the mRNA half-life and its m^5^C methylation status in mouse embryo stem cells, and the positive association is also confirmed on mouse whole brain dataset (*p* value = 0.0374). To further exclude the impact of mRNA expression in calling methylation status, we also extracted the genes whose log_2_ expression levels fall between 7 and 11, and then fit their mRNA half-life with a local regression. As shown in Figure 8, compared with the genes of a similar expression level but without an m^5^C site, the half-life of the mRNAs that carry m^5^C sites is clearly longer and the pattern is consistent in both mouse brain and ESC.

### 3.9. Dysregulation of RNA Methylome after Simian Retrovirus Infection

**Figure 9:**
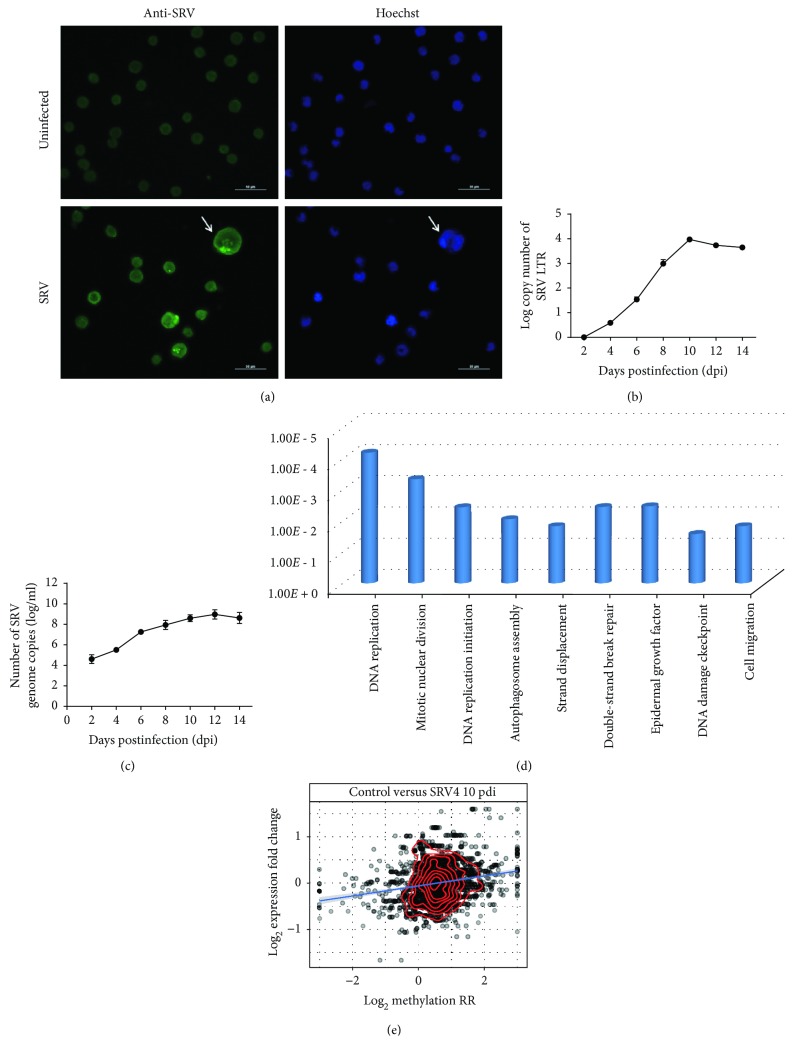
Dysregulation of RNA methylome after SRV infection of Jurkat cell. (a) At 10 days postinfection, uninfected or SRV-infected Jurkat cells were stained with SRV antibodies (green). Nuclei were visualized by Hoechst staining (blue). Arrows indicate the syncytium of infected cells. Scale bar: 50 *μ*M. (b) The relative level of SRV-LTR in infected Jurkat cells was measured every two days by real-time PCR. GAPDH was used as the internal control. The relative level of SRV-LTR at each time point was normalized to the data at 2 dpi; mean ± SD, *n* = 3. (c) The absolute copy number of the SRV genome in culture medium was measured every two days by real-time PCR. SRV-LTR and SRV genome were not detected in all uninfected cells and culture medium, respectively; mean ± SD, *n* = 3. (d) The differentially methylated genes are enriched with the following functions including DNA replication (*p* value = 6.07*E* − 5), mitotic nuclear division (*p* value = 4.37*E* − 4), DNA replication initiation (*p* value = 3.48*E* − 3), autophagosome assembly (*p* value = 8.54*E* − 3), strand displacement (*p* value = 1.42*E* − 2), double-strand break repair via homologous recombination (*p* value = 3.42*E* − 3), and so on, while hypomethylated genes are enriched with the following biological processes including negative regulation of epidermal growth factor receptor signaling pathway (*p* value = 3.24*E* − 3), DNA damage checkpoint (*p* value = 2.54*E* − 2), cell migration (*p* value = 1.42*E* − 2), and so on (see Figure 9 and Excel Sheet S5). (e) A weak but positive correlation (Pearson correlation = 0.07) is observed between RNA methylation level and expression level, which is consistent with our previous result; however, there are 23 genes that carry hyper- and hypomethylated sites simultaneously, which implies that RNA m^5^C carries more complicated biomolecular functions.

Simian retrovirus (SRV) infection of Jurkat T lymphocytes (Jurkat cells) was confirmed by syncytia formation, of which the membrane of the neighboring cells fused to one another. At 10 days postinfection, the formation of syncytium was observed among the Jurkat cells incubated with SRV (Figure 9(a)). The syncytium of Jurkat cells contains multiple nuclei and its size is dramatically larger than a single cell. SRV long terminal repeats (LTRs), which are reverse-transcribed from the RNA genome during the infection, contain critical sequences necessary for the integration, synthesis, and expression of viral DNA [1]. Therefore, the extent of SRV infection was assayed by monitoring SRV-LTR expression in Jurkat cells through quantitative real-time PCR. As shown in Figure 9(b), the copy number of SRV-LTR was gradually increased from 2 days to 10 days postinfection and then tended to be stable afterwards. Taken together, these results indicated that SRV was able to infect Jurkat cells and the infection reached maximum level after 10 days postinfection.

In order to investigate whether SRV could replicate in Jurkat cells, the SRV virions released in the culture medium were determined by measuring viral genome copy number through quantitative real-time PCR. As shown in Figure 9(c), the copy number of the SRV genome was gradually increased from 2 days to 14 days postinfection, suggesting that SRV was able to replicate in Jurkat cells.

We then measured the RNA methylome with bisulfite sequencing. A total of 2475 m^5^C sites located on 517 genes are reported as differentially methylated 10 days postinfection of SRV with QNB *p* value < 0.05. Among them, 389 sites located on 158 genes are hypomethylated, while 2086 sites from 382 genes are hypermethylated. A gene ontology analysis using the DAVID website suggests that the differentially methylated genes are related to virus infection, specifically, hypermethylated genes are enriched with DNA replication (*p* value = 6.07*E* − 5), mitotic nuclear division (*p* value = 4.37*E* − 4), DNA replication initiation (*p* value = 3.48*E* − 3), autophagosome assembly (*p* value = 8.54*E* − 3), strand displacement (*p* value = 1.42*E* − 2), double-strand break repair via homologous recombination (*p* value = 3.42*E* − 3), and so on, while hypomethylated genes are enriched with the following biological processes including negative regulation of epidermal growth factor receptor signaling pathway (*p* value = 3.24*E* − 3), DNA damage checkpoint (*p* value = 2.54*E* − 2), cell migration (*p* value = 1.42*E* − 2), and so on (see Figure 9 and Excel Sheet S3). Similar to before, a positive correlation (0.07) is observed between RNA methylation level and expression level; however, as there are 23 genes that carry hyper- and hypomethylated sites simultaneously, it is expected that RNA m^5^C carries more complicated biomolecular functions.

## 4. Discussion and Conclusion

The distribution of m^5^C methylation in mRNA has been mysterious with inconsistent evidence reported from previous studies [35, 37]. Here, we profiled the human and mouse m^5^C epitranscriptome using RNA BS-Seq data in human MCF10A, human MDA468, mouse ESC, and mouse whole brain cells. To eliminate the data sample bias, we employed a rigorous quality control procedure by filtering false positive m^5^C sites due to the secondary structure and performed a comprehensive comparative analysis on cross-species conserved locus, cross-sample comparison of topological transcriptome distributions of m^5^C, and differential m^5^C analysis. Our analysis clearly shows that m^5^C is enriched at the 5′UTR in human and mouse cells, confirming the discovery of a few independent studies [35, 36, 46]. Additionally, an unambiguous correlated methylation pattern is observed on 5′UTRs, but not on CDS and 3′UTR, in different mouse and human cell lines/tissues, suggesting a more complex aggregation pattern of m^5^C that may be further characterized. Together, these observations strongly imply the functional relevance of m^5^C RNA methylation and 5′UTR of mRNA. It is important to note that, although we failed to observe a correlated m^5^C methylation pattern on CDS and 3′UTR regions of mRNA, it is still possible that such pattern may emerge on strictly matched cell lines/tissues.

When comparing the DNA and RNA methylome in matched cell lines in human and mouse, a negative correlation in the methylation level is observed on matched locus on DNA and RNA, which is quite surprising given that the methyltransferase of DNA and RNA may share strong sequence homology [52]. This anticorrelation pattern is consistent at all four CG-containing trinucleotide contexts and ruled out the possibility of sample contamination or off-target effect, which should both lead to false positive correlation in data. It is possible that there exists an underlying biomolecular mechanism that functions on the matched locus of DNA and RNA in parallel to ensure their orchestrated methylation status.

Similar to DNA methylation, a clustering effect of m^5^C on mRNA is also observed in both human and mouse. The local dependency, that is, the adjacent cytosine locus often exhibits a similar methylation status, has been widely used in DNA methylation data analysis for more robust and accurate quantification of epigenetics status [57–59]. It is reasonable to expect that similar statistical approaches may be carried over into the field of single-base resolution RNA methylation data to enhance the analysis of bisulfite RNA methylation sequencing data. It is worth mentioning that, around 30%–43% of m^5^C residuals exist in pairs in our results after filtering potential secondary structures that may lead to incomplete conversion and false positive m^5^C sites. The number may be over- or underestimated because of the unfiltered secondary structure, which leads to an overestimation of the clustering effect, and structured regions excluded from the analysis, which may affect the estimation in both directions. It is necessary to develop a more sensitive unbiased approach that can eliminate the impact of the RNA structure to more accurately assess the distribution of transcriptome m^5^C modification.

Intriguingly, we observed a strong enrichment of m^5^C methylation on mitochondrial transcripts with more than 50 folds of enrichment. Previously, it was reported that methyltransferase NSUN5 can regulate mitochondrial gene expression [54], and we speculate that RNA m^5^C methylation may play a more vital regulatory role in mitochondria-related biological processes.

Additionally, in order to have a glimpse of the dynamics of m^5^C on mRNA, differential RNA methylation analysis was performed between breast cancer cell line MDA-MB-468 and the control cell line MCF10A; a total of 47 genes are reported to be differentially methylated, including RTN4, NME2, CASP14, HSPB1, RPL11, and RPS3, which are related to apoptosis and programmed cell death. Although we showed previously that m^5^C on mRNA are more likely to be linked to the 5′UTR function, it is observed that the differential methylation sites between the breast cancer cell line and normal control cell lines are mostly located on the CDS and 3′UTR. These observations together implied a profound role of m^5^C methylation on different regions of mRNA and in cancer pathology.

Interestingly, an overall positive correlation between RNA m^5^C methylation and RNA expression level is observed in our mouse and human datasets, which added to the growing importance of mRNA m^5^C methylation in regulating gene expression. Although the specific molecular mechanism is not yet clear, the observed positive correlation between RNA m^5^C and RNA expression echoes our previous observed anticorrelation between DNA and RNA m^5^C methylation from a different perspective, because it has been well established that DNA methylation is anticorrelated with RNA expression. However, as it is known that the most abundant RNA modification m^6^A methylation may enhance or reduce the stability of the RNA molecule through interaction with different m^6^A readers [14, 60] or regulate RN-protein interaction [13], it is reasonable to assume that RNA m^5^C may have versatile functionalities, and may get dominated by a distinct mechanism under a specific condition.

In summary, our study presented an in-depth topological characterization of the m^5^C RNA methylome in human and mouse. There are interesting patterns depicted and quantified, which call for further studies to explain novel biomolecular mechanisms.

## Abbreviations

m^6^A: *N* ^6^-Methyladenosine
hm^5^C: Hydroxymethylcytosine
*Ψ*: Pseudouridine
m^1^A: *N* ^1^-Methyladenosine
m^5^C: 5-Methylcytosine
BS-Seq: Bisulfite sequencing
MDA-MB-468: MDA468
MEF: Mouse embryo fibroblast
MEF-Dnmt2^−^: Mouse embryo fibroblast with Dnmt2 knockdown
WGBS: Whole genome bisulfite sequencing
RRBS: Reduced representation bisulfite sequencing
mRatio: Methylation ratio
OR: Odds ratio
DMS: Differential methylation site
lncRNA: Long noncoding RNA
sncRNA: Small noncoding RNA.

## Supplementary Materials

Supplementary Materials File 1. SI_figures_and_tables: additional figures and statistical analysis. File 2. SI_sheets_S1: differential methylation information between MCF10A and MDA468. File 3. SI_sheets_S2: differential methylation information between mouse ESC and mouse brain. File 4. SI_sheets_S3: differential methylation information between SRV-infected and -uninfected Jurkat cells.
