# Supplementary material for: Topological Characterization of Human and Mouse m5C Epitranscriptome Revealed by Bisulfite Sequencing
Source: Int J Genomics. 2018 Jun 13;2018:1351964. doi: 10.1155/2018/1351964 (PMC6020461; doi:10.1155/2018/1351964)
Supplement: Supplementary Materials — File 1. SI_figures_and_tables: additional figures and statistical analysis. File 2. SI_sheets_S1: differential methylation information between MCF10A and MDA468. File 3. SI_sheets_S2: differential methylation information between mouse ESC and mouse brain. File 4. SI_sheets_S3: differential methylation information between SRV-infected and -uninfected Jurkat cells. [file 1351964.f1.pdf]

Supporting Information – Tables and Figures

Topological characterization of m^{5}C epitranscriptome in human and mouse

Table S1. RNA m^{5}C methylation is enriched on 5'UTR of mRNA.

Region	Species	Cell	OR	p-value	Enriched
**5'UTR**	Human	MCF10A	3.138452587	3.42E-04	Yes
		MDA468	4.801521457	1.87E-82	Yes
	Mouse	Brain	1.600523029	4.68E-09	Yes
		ESC	2.744182653	0	Yes
**CDS**	Human	MCF10A	0.737478238	6.39E-09	Yes
		MDA469	0.607316233	0.999993833	No
	Mouse	Brain	0.921190561	1	No
		ESC	0.923214737	0	Yes
**3'UTR**	Human	MCF10A	0.963777736	1	No
		MDA470	0.971245244	1	No
	Mouse	Brain	1.013596292	1.19E-02	Yes
		ESC	0.616944645	1	No

Table shows the enrichment odds ratio of transcriptome m^{5}C at different regions of mRNA. The p-values are calculated from Fisher's exact test. m^{5}C is consistently enriched at 5'UTRs on mRNA.

Table S2. Correlated methylation status at the conserved locus in human and mouse

Region	Cell	p-values	Conserved	OR	95%CI lower	95%CI upper
5'UTR	Brain:MCF10A	5.08E-09	Yes	5.518488	3.276153	8.829768357
	Brain:MDA468	1.23E-06	Yes	5.045639	2.72693	8.699535575
	ESC:MCF10A	0.111851	Marginal	1.544756	0.864908	2.575701499
	ESC:MDA46	0.071708	Marginal	1.7308	0.920304	3.010289159
3'UTR	Brain:MCF10A	0.589205	No	1.13382	0.028268	6.546466183
	Brain:MDA468	0.23312	No	3.929861	0.094844	24.53885881
	ESC:MCF10A	0.428824	No	1.806182	0.045014	10.43730286
	ESC:MDA46	1	No	0	0	19.86828857
CDS	Brain:MCF10A	0.242101	No	1.509041	0.60286	3.137921831
	Brain:MDA468	0.504139	No	1.306364	0.26698	3.876797368
	ESC:MCF10A	0.127449	Marginal	1.552574	0.842335	2.630804016
	ESC:MDA46	1	No	0.838203	0.171255	2.48859736

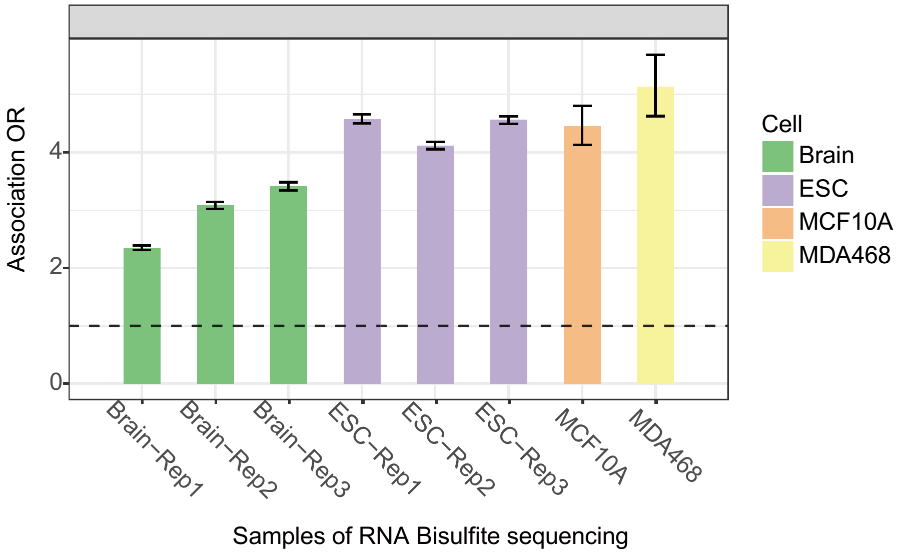

Figure S1. Association between reported methylated sites and double stranded RNA structures before filtering. The reported m^5C sites by MeRanTK approach are clearly enriched with double stranded RNA structure before the filtering, and the pattern is consistent in all the 4 samples. The odds ratio (OR) was calculated from Fisher's exact test and the error bar shows the 95% confidence interval of OR estimation.

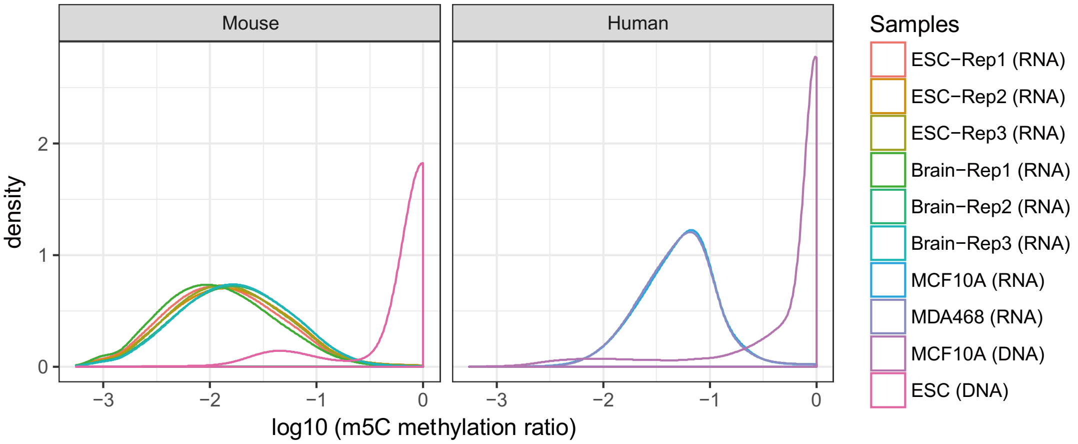

Figure S2. Distribution of RNA and DNA methylation ratio. The diagram shows the distribution of RNA and DNA methylation ratio under 4 different conditions. In general, DNA methylation ratio is much higher than RNA. The distribution patterns of biological replicates are relatively close.

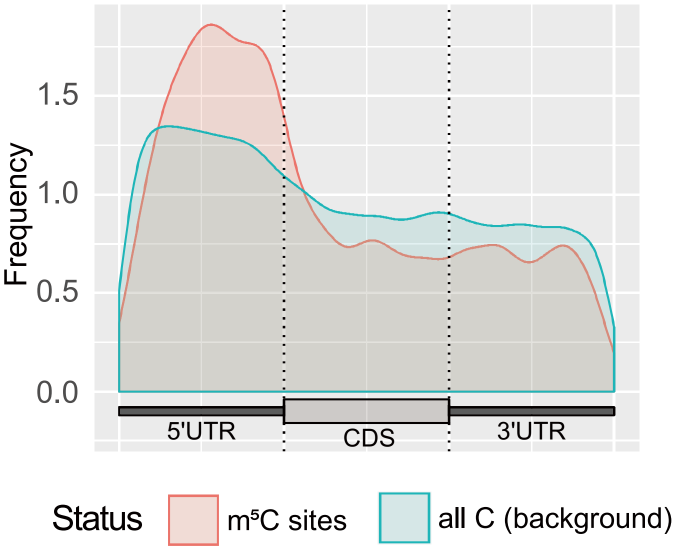

Figure S3. mRNA m^5C sites are enriched at 5'UTRs in HeLa cells. The 10275 RNA methylation sites from Hela cell line are extracted from a previous study (1). Compared with the background (all the transcriptomic Cytosine sites), the reported mRNA m^5C methylation sites are more enriched on 5'UTR. Prominent enrichment is not observed on 3'UTR. This trend is consistent with the other 4 samples tested (See **Figure 1** of the main text). Figure is plotted using default setting of Guitar package (2) with human hg19 genome assembly and UCSC gene annotation.

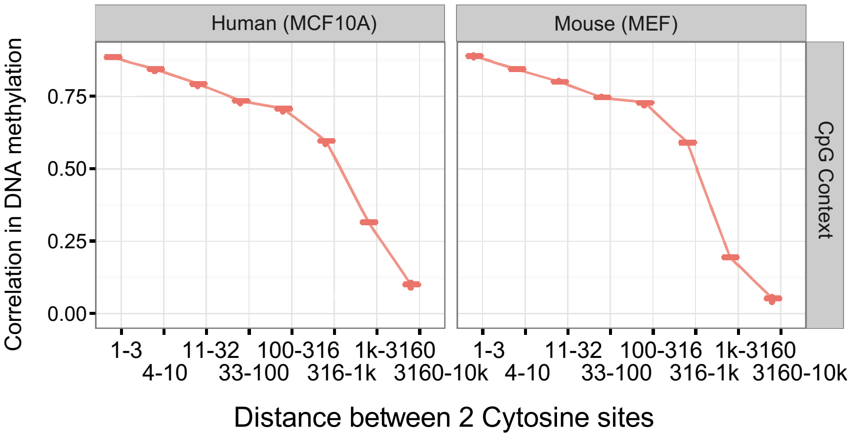

Figure S4. Strong clustering effect exists in DNA methylation under CpG context. Figure shows the correlation in methylation level between two cytosine sites of a certain distance. Strong correlation is observed between cytosine sites with a smaller distance.

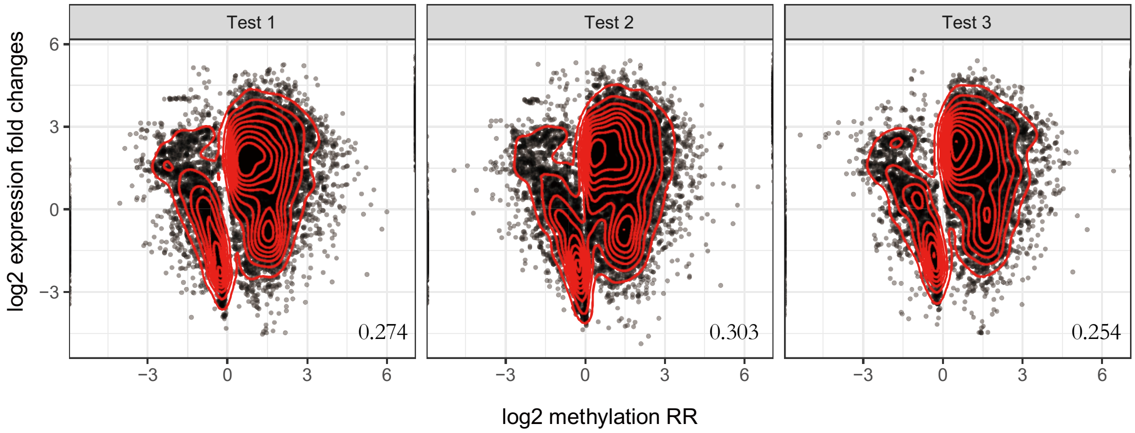

Figure S5. Positive correlations are observed between expression and mRNA m^5C methylation fold change. To eliminate the interference of dependent noise between expression and methylation data, the samples are further divided for different purposes, and the test was repeated for 3 times using different ways of sample grouping. A strong, consistent and significant positive correlation is observed (0.274, 0.303 and 0.254) between log2 expression fold change and log2 methylation fold change when comparing mouse embryo stem cells with brain cells, suggesting that increased methylation level is likely to be associated with increased expression level, but the underlying mechanism is not yet clear.

REFERENCE

1. Squires, J.E., Patel, H.R., Nousch, M., Sibbritt, T., Humphreys, D.T., Parker, B.J., Suter, C.M. and Preiss, T. (2012) Widespread occurrence of 5-methylcytosine in human coding and non-coding RNA. *Nucleic acids research*, gks144.
2. Cui, X., Wei, Z., Zhang, L., Liu, H., Sun, L., Zhang, S.-W., Huang, Y. and Meng, J. (2016) Guitar: An R/Bioconductor Package for Gene Annotation Guided Transcriptomic Analysis of RNA-Related Genomic Features. *BioMed Research International*, **2016**, 8.

SI_Sheets_S1

DM_MDA468-MCF10A

seqnames	start	end	strand	log2.RR	pvalue	DESeq2_log2FC	Gene Symbol
chrM	295	295	+	1.0030	0.00021644	1.6228	NA
chrM	12619	12619	-	2.0785	0.03297760	0.7119	NA
chrM	12184	12184	-	1.8780	0.02598220	(0.1410)	NA
chrM	12295	12295	-	1.4099	0.00347779	0.8877	NA
chrM	12319	12319	-	1.5821	0.00245258	0.8297	NA
chrM	12284	12284	-	1.9939	0.00258367	0.6871	NA
chrM	12301	12301	-	2.2546	0.02914261	0.7097	NA
chrM	12316	12316	-	0.9299	0.00974915	0.8860	NA
chrM	12245	12245	-	1.1695	0.01486688	0.7419	NA
chrM	12294	12294	-	1.8254	0.00159248	0.8893	NA
chrM	15864	15864	-	1.0747	0.02298167	0.8844	NA
chrM	12317	12317	-	1.4446	0.00325778	0.9271	NA
chrM	12187	12187	-	1.4089	0.01371059	0.1939	NA
chrM	12186	12186	-	1.8400	0.01610350	0.0140	NA
chr1	26799100	26799100	+	1.4239	0.01943261	0.0877	HMGN2
chr1	26799094	26799094	+	2.0048	0.01596889	(0.0000)	HMGN2
chr1	26799096	26799096	+	1.8338	0.01575193	(0.0578)	HMGN2
chr1	26799097	26799097	+	1.7815	0.02051692	(0.0011)	HMGN2
chr1	26799088	26799088	+	1.9933	0.02780126	(0.3456)	HMGN2
chr1	26799099	26799099	+	1.8962	0.00813283	0.0735	HMGN2
chr1	26799093	26799093	+	2.5782	0.00551700	0.0056	HMGN2
chr1	26799087	26799087	+	2.5816	0.01079698	(0.2384)	HMGN2
chr1	26799091	26799091	+	3.6798	0.00115739	(0.1009)	HMGN2
chr1	41327425	41327425	-	Inf	0.02967750	2.6847	CITED4
chr1	209605621	209605621	+	2.0357	0.01925418	0.8645	MIR205HG
chr1	209605619	209605619	+	1.6277	0.00486650	0.8642	MIR205HG
chr1	209605625	209605625	+	1.5275	0.02324784	0.7733	MIR205HG
chr1	209605622	209605622	+	1.7787	0.00647039	0.8622	MIR205HG
chr1	209605628	209605628	+	1.4557	0.01833902	0.8883	MIR205HG
chr1	209605627	209605627	+	1.5386	0.02570147	0.9033	MIR205HG

chr1	20960562 4	20960562 4	+	1.6281	0.00833441	0.8096	MIR205HG
chr6	30460963	30460963	+	1.1467	0.00887000	1.6978	NA
chr6	30460961	30460961	+	0.9504	0.00638062	1.6988	NA
chr7	75932209	75932209	+	1.2701	0.02567389	0.9649	HSPB1
chr8	14601728 4	14601728 4	-	2.4353	0.00042198	0.2769	RPL8
chr8	14601728 5	14601728 5	-	1.9399	0.00283365	0.2093	RPL8
chr8	14601728 3	14601728 3	-	2.2246	0.00044037	0.2361	RPL8
chr11	810019	810019	+	1.3051	0.03459002	(0.0088)	RPLP2
chr11	810021	810021	+	1.2345	0.03449497	(0.0409)	RPLP2
chr11	810036	810036	+	1.8146	0.02202777	(0.2267)	RPLP2
chr11	810024	810024	+	1.3823	0.03451201	(0.0391)	RPLP2
chr11	810018	810018	+	1.0798	0.02963545	0.1430	RPLP2
chr11	61734952	61734952	-	0.2656	0.02352192	(1.4369)	FTH1
chr11	61734956	61734956	-	0.1421	0.01781051	(1.3754)	FTH1
chr11	61734942	61734942	-	0.3599	0.04166230	(1.3864)	FTH1
chr11	61734950	61734950	-	0.3592	0.04067754	(1.3468)	FTH1
chr11	61734934	61734934	-	0.1952	0.02964588	(1.3401)	FTH1
chr11	61734955	61734955	-	0.1607	0.04216580	(1.2916)	FTH1
chr11	67817223	67817223	+	Inf	0.00304444	2.4040	TCIRG1
chr11	67817217	67817217	+	Inf	0.01060542	2.4852	TCIRG1
chr11	67817220	67817220	+	Inf	0.00340341	2.4742	TCIRG1
chr11	67817222	67817222	+	Inf	0.00151361	2.4477	TCIRG1
chr11	67817219	67817219	+	Inf	0.00252046	2.4648	TCIRG1
chr11	67817216	67817216	+	Inf	0.00531521	2.4813	TCIRG1
chr11	67817226	67817226	+	Inf	0.00412166	2.6548	TCIRG1
chr11	67817225	67817225	+	Inf	0.00803745	2.6429	TCIRG1
chr15	90328300	90328300	-	0.0000	0.03501987	(2.1380)	ANPEP
chr16	84651628	84651628	-	3.6959	0.04488490	(0.6192)	COTL1
chr16	84651630	84651630	-	3.8882	0.04794005	(0.5390)	COTL1
chr17	39777000	39777000	-	1.4036	0.00602756	1.8991	JUP
chr17	39776999	39776999	-	0.1194	0.00125961	1.8810	JUP
chr19	1038682	1038682	+	Inf	0.00130553	1.1744	CNN2
chr19	1038671	1038671	+	Inf	0.00255257	1.1832	CNN2
chr19	1038683	1038683	+	Inf	0.00107319	1.1419	CNN2
chr19	1038675	1038675	+	Inf	0.00078584	1.1795	CNN2
chr19	1038680	1038680	+	Inf	0.00012044	1.2601	CNN2
chrM	7328	7328	+	-0.5239	0.03899141	(0.8133)	NA

chrM	7334	7334	+	-0.4835	0.03142980	(0.9712)	NA
chrM	2758	2758	+	-1.7976	0.03150925	1.0289	NA
chrM	8037	8037	+	-2.0197	0.03178848	1.0142	NA
chrM	8031	8031	+	-2.3579	0.02782147	1.0737	NA
chrM	8053	8053	+	-1.8220	0.03651928	1.0127	NA
chrM	2901	2901	+	-1.6228	0.04641736	0.9079	NA
chrM	8077	8077	+	-1.7851	0.00836431	0.9757	NA
chrM	8055	8055	+	-1.7949	0.01178239	0.9499	NA
chrM	2893	2893	+	-1.6993	0.01503339	0.8373	NA
chrM	8071	8071	+	-2.0395	0.01378801	0.9892	NA
chrM	7337	7337	+	-0.6521	0.01831668	(0.9948)	NA
chrM	8050	8050	+	-1.7501	0.02547098	0.9921	NA
chrM	2902	2902	+	-1.6033	0.04540561	0.9036	NA
chrM	8713	8713	+	-0.4310	0.03364071	(0.7562)	NA
chrM	8069	8069	+	-1.5873	0.02794642	0.9814	NA
chrM	1867	1867	+	-2.2656	0.01008787	1.0920	NA
chrM	2886	2886	+	-1.5686	0.02798062	0.9125	NA
chrM	8081	8081	+	-1.6973	0.03497701	1.0435	NA
chr1	24019135	24019135	+	-0.6336	0.04431446	(0.6873)	RPL11
chr1	24019143	24019143	+	-2.5534	0.02659312	(0.6581)	RPL11
chr1	153507293	153507293	-	-2.2861	0.03650156	(1.0049)	S100A6
chr1	153507262	153507262	-	-1.2043	0.01664121	(0.8083)	S100A6
chr1	153507279	153507279	-	-0.4264	0.04878029	(0.9040)	S100A6
chr1	154938904	154938904	-	#NAME?	0.00404141	(2.7105)	SHC1
chr2	55277254	55277254	-	-0.8720	0.02395047	(2.1441)	RTN4
chr2	55277255	55277255	-	#NAME?	0.00847862	(2.1652)	RTN4
chr2	55277257	55277257	-	#NAME?	0.01109803	(2.1725)	RTN4
chr2	55277258	55277258	-	#NAME?	0.00514037	(2.3979)	RTN4
chr2	85133611	85133611	+	-1.7567	0.01941391	(0.4185)	TMSB10
chr2	216285454	216285454	-	#NAME?	0.00558349	(3.2027)	FN1
chr2	216296634	216296634	-	#NAME?	0.00805242	(3.1697)	FN1
chr2	216296631	216296631	-	#NAME?	0.02911234	(3.1804)	FN1
chr2	216230300	216230300	-	#NAME?	0.04084190	(3.1554)	FN1

chr2	216298084	216298084	-	#NAME?	0.00122074	(3.2056)	FN1
chr5	40834349	40834349	-	-2.3682	0.01881507	0.4874	RPL37
chr5	40834331	40834331	-	-2.8418	0.01310316	0.4326	RPL37
chr5	40834330	40834330	-	-2.7713	0.02737153	0.4417	RPL37
chr5	40834333	40834333	-	-3.7792	0.01219405	0.4569	RPL37
chr5	149826521	149826521	-	-2.3989	0.00546336	(0.9093)	RPS14
chr5	179233649	179233649	-	-0.5850	0.02623801	(1.5785)	MGAT4B
chr6	34205006	34205006	+	-0.4095	0.00015531	(1.2290)	HMGA1
chr6	34208627	34208627	+	-0.7228	0.03561993	(0.5363)	HMGA1
chr7	5567650	5567650	-	-1.5636	0.04506241	(0.1273)	ACTB
chr7	54823535	54823535	-	-2.0155	0.04003392	1.8753	SEC61G
chr7	54823516	54823516	-	-3.0968	0.01066809	2.1061	SEC61G
chr7	54823513	54823513	-	-3.3635	0.04719659	2.3872	SEC61G
chr7	75932358	75932358	+	-3.5500	0.01010825	0.3860	HSPB1
chr7	75932377	75932377	+	-3.4814	0.01524517	0.5227	HSPB1
chr7	75932362	75932362	+	#NAME?	0.00233464	0.3831	HSPB1
chr8	101733698	101733698	-	-0.8627	0.00914372	(0.8529)	PABPC1
chr9	136216892	136216892	+	-0.9317	0.04338719	(0.5014)	RPL7A
chr11	61732229	61732229	-	-1.4551	0.04339167	0.3602	FTH1
chr11	61732227	61732227	-	-1.4292	0.01030176	0.2948	FTH1
chr11	61732251	61732251	-	-1.4121	0.03341563	0.3936	FTH1
chr11	61732226	61732226	-	-2.2436	0.00222210	0.3210	FTH1
chr11	61732225	61732225	-	-1.7529	0.00756333	0.3171	FTH1
chr11	62334441	62334441	-	-1.3334	0.04355345	(0.1346)	EEF1G
chr11	62334462	62334462	-	-2.1679	0.01252382	(0.0936)	EEF1G
chr11	65306695	65306695	-	-0.4079	0.02790451	(0.7102)	LTBP3
chr11	75111844	75111844	+	-1.0508	0.03727205	(0.4757)	RPS3
chr12	6875851	6875851	+	-0.3907	0.04185005	(0.4587)	PTMS
chr12	6875852	6875852	+	-0.5039	0.03241342	(0.4785)	PTMS
chr12	57106952	57106952	-	-1.4124	0.02594472	(0.6397)	NACA
chr15	90328288	90328288	-	#NAME?	0.01311644	(2.5430)	ANPEP
chr17	16342696	16342696	+	-2.1307	0.00123664	(1.1996)	LRRC75A-AS1
chr17	48165718	48165718	+	-0.1473	0.03337030	(2.3145)	ITGA3
chr17	48165719	48165719	+	-0.1612	0.04054757	(2.2730)	ITGA3
chr17	49244197	49244197	+	-1.3810	0.00917170	(1.5091)	NME2

chr17	48165717	48165717	+	-0.1227	0.03620798	(2.2924)	ITGA3
chr17	79478518	79478518	-	-0.9225	0.00035980	(1.1371)	ACTG1
chr17	80195269	80195269	+	-0.8251	0.03573892	(1.8833)	SLC16A3
chr19	1104123	1104123	+	-1.1825	0.02551551	(0.9110)	GPX4
chr19	1104122	1104122	+	-1.1157	0.02048137	(0.9778)	GPX4
chr19	5690535	5690535	+	-1.4388	0.04328661	(0.2994)	RPL36
chr19	5690542	5690542	+	-2.3001	0.00497960	(0.3524)	RPL36
chr19	3982286	3982286	-	-0.0128	0.04992637	(1.0997)	EEF2
chr19	3976641	3976641	-	-2.1916	0.00507570	(0.9773)	EEF2
chr19	3982257	3982257	-	-0.6941	0.01267919	(1.1621)	EEF2
chr19	3981348	3981348	-	-1.0455	0.04284370	(0.4495)	EEF2
chr19	15168759	15168759	+	-1.2251	0.04404534	0.4982	CASP14
chr19	15168767	15168767	+	-1.6333	0.04524949	0.4495	CASP14
chr19	15168755	15168755	+	-1.2251	0.04404534	0.4982	CASP14
chr19	15168757	15168757	+	-1.0396	0.02268098	0.2584	CASP14
chr20	57466760	57466760	+	-0.6752	0.03928924	(0.6982)	GNAS
chr20	57466739	57466739	+	-0.3475	0.03545187	(0.8345)	GNAS
chr20	57466745	57466745	+	-0.3115	0.03844303	(0.6976)	GNAS
chr20	57466761	57466761	+	-0.3399	0.03308692	(0.7029)	GNAS
chr20	57466738	57466738	+	-0.3835	0.01472380	(0.7992)	GNAS
chr20	57466742	57466742	+	-0.2135	0.02755269	(0.8188)	GNAS
chr20	57466744	57466744	+	-0.1567	0.04963075	(0.7186)	GNAS
chr20	57466571	57466571	+	-1.9346	0.03503080	(1.1705)	GNAS
chr20	57466741	57466741	+	-0.2061	0.03297448	(0.7953)	GNAS
chr22	36678098	36678098	-	-1.6762	0.03065017	(1.4254)	MYH9
chr22	39713589	39713589	-	-0.1994	0.01348909	(1.7282)	RPL3
chrX	12994904	12994904	+	-1.0723	0.04403199	(0.0343)	TMSB4X

GO - DM_MDA468-MCF10A

Category	Term	PValue	Fold Enrichment	FDR
SP_PIR_KEYWORDS	acetylation	2.16E-14	4.866540164	2.62E-11
GOTERM_BP_FAT	GO:0006414~translational elongation	3.16E-13	35.0796794	4.64E-10
SP_PIR_KEYWORDS	ribosome	2.51E-12	56.462818	3.03E-09
SP_PIR_KEYWORDS	protein biosynthesis	5.39E-12	26.79647923	6.52E-09
GOTERM_CC_FAT	GO:0005829~cytosol	1.95E-11	5.285789474	2.36E-08
GOTERM_CC_FAT	GO:0022626~cytosolic	9.58E-11	35.50555556	1.16E-07

	ribosome			
GOTERM_CC_FAT	GO:0022625~cytosolic large ribosomal subunit	1.39E-09	58.86447368	1.67E-06
GOTERM_BP_FAT	GO:0006412~translation	2.62E-09	11.67716875	3.85E-06
GOTERM_CC_FAT	GO:0033279~ribosomal subunit	3.85E-09	22.46835938	4.65E-06
SP_PIR_KEYWORDS	ribosomal protein	5.20E-09	21.9243921	6.29E-06
GOTERM_CC_FAT	GO:0044445~cytosolic part	1.50E-08	18.92072368	1.81E-05
GOTERM_MF_FAT	GO:0003735~structural constituent of ribosome	2.91E-08	17.38794643	3.45E-05
GOTERM_CC_FAT	GO:0015934~large ribosomal subunit	4.70E-08	33.3858209	5.68E-05
KEGG_PATHWAY	hsa03010:Ribosome	9.20E-08	18.70344828	8.56E-05
SP_PIR_KEYWORDS	ribonucleoprotein	1.12E-07	14.7734255	1.35E-04
GOTERM_MF_FAT	GO:0005198~structural molecule activity	1.92E-07	6.655323344	2.28E-04
GOTERM_CC_FAT	GO:0005840~ribosome	2.21E-07	13.37651163	2.67E-04
GOTERM_CC_FAT	GO:0030529~ribonucleoprotein complex	2.45E-07	7.445825243	2.96E-04
SP_PIR_KEYWORDS	cytoplasm	3.58E-06	2.886404562	0.004331813
SP_PIR_KEYWORDS	phosphoprotein	4.33E-06	1.954737974	0.005239862
GOTERM_CC_FAT	GO:0043232~intracellular non-membrane-bounded organelle	1.64E-05	2.584957627	0.019804179
GOTERM_CC_FAT	GO:0043228~non-membrane-bounded organelle	1.64E-05	2.584957627	0.019804179
GOTERM_CC_FAT	GO:0005625~soluble fraction	3.87E-05	8.167412141	0.046741671
GOTERM_CC_FAT	GO:0001726~ruffle	4.94E-05	23.84701493	0.059628979
KEGG_PATHWAY	hsa05110:Vibrio cholerae infection	1.19E-04	18.16071429	0.110714732
SP_PIR_KEYWORDS	rrna-binding	5.91E-04	80.81932773	0.71176962
GOTERM_CC_FAT	GO:0031252~cell leading edge	7.98E-04	11.57789855	0.959342333
SP_PIR_KEYWORDS	blocked amino end	9.90E-04	19.91200828	1.189626736
GOTERM_MF_FAT	GO:0003723~RNA binding	0.001122335	4.068488858	1.321108896
GOTERM_CC_FAT	GO:0000267~cell fraction	0.001192774	3.245660203	1.430785214
SP_PIR_KEYWORDS	actin-binding	0.001850709	9.270773087	2.214277078
SP_PIR_KEYWORDS	cytoskeleton	0.002140865	5.040618449	2.557325732
KEGG_PATHWAY	hsa04810:Regulation of actin cytoskeleton	0.002836553	5.67627907	2.608809198
GOTERM_MF_FAT	GO:0019843~rRNA binding	0.00339162	33.57672414	3.943612386
GOTERM_MF_FAT	GO:0008092~cytoskeletal protein binding	0.003643177	4.507986111	4.230359791
SP_PIR_KEYWORDS	cell motility	0.004258629	457.9761905	5.027905801
GOTERM_BP_FAT	GO:0030036~actin cytoskeleton organization	0.00473306	7.126000843	6.741410077
KEGG_PATHWAY	hsa05412:Arrhythmogenic right ventricular cardiomyopathy (ARVC)	0.00518194	10.70526316	4.719824528

SP_PIR_KEYWORDS	disease mutation	0.0054868	2.878542995	6.434229397
GOTERM_BP_FAT	GO:0030029~actin filament-based process	0.005934037	6.682473819	8.383289119
SP_PIR_KEYWORDS	microfilament	0.006381304	305.3174603	7.446435928
KEGG_PATHWAY	hsa05414:Dilated cardiomyopathy	0.008810366	8.843478261	7.905265722
SMART	SM00152:THY	0.009329317	201.7555556	7.073111491
GOTERM_CC_FAT	GO:0030863~cortical cytoskeleton	0.009745974	19.56428571	11.15338045
INTERPRO	IPR016323:Thymosin beta-4, chordata	0.012246701	158.6571429	13.28094981
INTERPRO	IPR001152:Thymosin beta-4	0.012246701	158.6571429	13.28094981
KEGG_PATHWAY	hsa04510:Focal adhesion	0.013521888	5.059701493	11.89958629
SP_PIR_KEYWORDS	methylation	0.014749801	7.569854388	16.44119635
GOTERM_BP_FAT	GO:0051235~maintenance of location	0.016046823	15.09821429	21.17828456
GOTERM_MF_FAT	GO:0003779~actin binding	0.016079713	4.978144172	17.46746686
GOTERM_MF_FAT	GO:0019900~kinase binding	0.016372653	7.253072626	17.75800223
GOTERM_MF_FAT	GO:0003729~mRNA binding	0.016710831	14.75340909	18.09224101
GOTERM_CC_FAT	GO:0042995~cell projection	0.01802548	3.209253945	19.71930997
GOTERM_BP_FAT	GO:0042989~sequestering of actin monomers	0.018050591	107.3650794	23.50697606
SP_PIR_KEYWORDS	structural protein	0.01902495	101.7724868	20.7199901
SP_PIR_KEYWORDS	deafness	0.019234604	13.73928571	20.92455843
GOTERM_MF_FAT	GO:0019899~enzyme binding	0.019352252	3.723613767	20.66040818
PIR_SUPERFAMILY	PIRSF001828:Thymosin_beta	0.020787901	92.45	17.08609608
GOTERM_CC_FAT	GO:0005856~cytoskeleton	0.020789735	2.313902969	22.4059737
GOTERM_CC_FAT	GO:0044448~cell cortex part	0.02642977	11.55	27.63369946
UP_SEQ_FEATURE	mutagenesis site	0.02681487	2.225288159	29.12099805
SP_PIR_KEYWORDS	rna-binding	0.027415737	4.240520282	28.53922567
SP_PIR_KEYWORDS	isopeptide bond	0.030290944	5.742648156	31.05139574
GOTERM_BP_FAT	GO:0032989~cellular component morphogenesis	0.031491021	4.056615089	37.54476344
GOTERM_BP_FAT	GO:0000904~cell morphogenesis involved in differentiation	0.037373511	5.280249805	42.89876679
INTERPRO	IPR004001:Actin, conserved site	0.038676631	49.58035714	36.62748615
GOTERM_MF_FAT	GO:0042802~identical protein binding	0.041327067	3.042890625	39.33675015
SP_PIR_KEYWORDS	methylated amino acid	0.041798543	45.79761905	40.31638304
GOTERM_BP_FAT	GO:0007010~cytoskeleton organization	0.042181889	3.69375273	46.95405007
KEGG_PATHWAY	hsa05416:Viral myocarditis	0.043534925	8.594366197	33.91435317
SP_PIR_KEYWORDS	elongation factor	0.043843105	43.61678005	41.83767464

GOTERM_BP_FAT	GO:0008104~protein localization	0.04819929	2.556311413	51.65099446
SP_PIR_KEYWORDS	cell shape	0.049951288	38.16468254	46.17325266
GOTERM_BP_FAT	GO:0042254~ribosome biogenesis	0.052649245	7.920374707	54.87188906
GOTERM_BP_FAT	GO:0006928~cell motion	0.054714401	3.39047619	56.29768312
GOTERM_BP_FAT	GO:0022604~regulation of cell morphogenesis	0.059720388	7.376226827	59.58149474
KEGG_PATHWAY	hsa05410:Hypertrophic cardiomyopathy (HCM)	0.060105763	7.178823529	43.8339952
UP_SEQ_FEATURE	cross-link:Glycyl lysine isopeptide (Lys-Gly) (interchain with G-Cter in ubiquitin)	0.067884997	6.860373295	58.94290704
GOTERM_BP_FAT	GO:0051129~negative regulation of cellular component organization	0.068780349	6.804828974	64.94729355
PIR_SUPERFAMILY	PIRSF002337:Actin	0.06898696	27.19117647	47.14236066
GOTERM_MF_FAT	GO:0003746~translation elongation factor activity	0.069717436	27.04791667	57.50771785
SMART	SM00268:ACTIN	0.070629369	25.86609687	43.63158861
GOTERM_MF_FAT	GO:0019901~protein kinase binding	0.071880881	6.623979592	58.66337694
GOTERM_CC_FAT	GO:0005938~cell cortex	0.072979252	6.56609589	59.949391
GOTERM_BP_FAT	GO:0001525~angiogenesis	0.073903048	6.528957529	67.67947456
GOTERM_BP_FAT	GO:0030837~negative regulation of actin filament polymerization	0.08155655	23.00680272	71.39387942
GOTERM_BP_FAT	GO:0031032~actomyosin structure organization	0.08155655	23.00680272	71.39387942
GOTERM_MF_FAT	GO:0045182~translation regulator activity	0.083633955	22.38448276	64.45423338
GOTERM_BP_FAT	GO:0032272~negative regulation of protein polymerization	0.084345897	22.2134647	72.64568081
GOTERM_CC_FAT	GO:0008305~integrin complex	0.084894964	22.03793103	65.74166102
SP_PIR_KEYWORDS	non-syndromic deafness	0.085808861	21.80839002	66.1915019
GOTERM_CC_FAT	GO:0012505~endomembrane system	0.087403991	2.451790281	66.85878277
INTERPRO	IPR004000:Actin/actin-like	0.091728897	20.34065934	67.13003658
GOTERM_BP_FAT	GO:0000902~cell morphogenesis	0.092638573	3.619047619	76.07233965
SP_PIR_KEYWORDS	sulfation	0.093596037	19.91200828	69.51280952
GOTERM_BP_FAT	GO:0042981~regulation of apoptosis	0.093985082	2.403695807	76.58942514
GOTERM_BP_FAT	GO:0043067~regulation of programmed cell death	0.097056035	2.380014075	77.73002267
GOTERM_BP_FAT	GO:0031333~negative regulation of protein complex assembly	0.098169127	18.94677871	78.13049236

GOTERM_BP_FAT	GO:0010941~regulation of cell death	0.098221097	2.371253287	78.1490253

SI_Sheets_S2

GO - hyper in ESC

Category	Term	PValue	Fold Enrichment	FDR
SP_PIR_KEYWORDS	acetylation	1.76E-45	2.6017	2.42E-42
SP_PIR_KEYWORDS	phosphoprotein	9.17E-43	1.7228	1.27E-39
SP_PIR_KEYWORDS	cytoplasm	2.94E-17	1.7595	4.05E-14
GOTERM_CC_FAT	GO:0030529~ribonucleoprotein complex	4.24E-17	3.5172	5.95E-14
SP_PIR_KEYWORDS	nucleus	1.36E-15	1.6165	1.84E-12
GOTERM_CC_FAT	GO:0043233~organelle lumen	8.82E-15	2.3032	1.23E-11
GOTERM_CC_FAT	GO:0070013~intracellular organelle lumen	2.08E-14	2.2850	2.93E-11
GOTERM_CC_FAT	GO:0031974~membrane-enclosed lumen	2.48E-14	2.2521	3.49E-11
SP_PIR_KEYWORDS	rna-binding	7.53E-13	3.0219	1.04E-09
SP_PIR_KEYWORDS	ribonucleoprotein	1.22E-12	3.9070	1.68E-09
GOTERM_MF_FAT	GO:0003723~RNA binding	8.88E-12	2.5301	1.31E-08
GOTERM_BP_FAT	GO:0006412~translation	1.50E-11	3.3691	2.60E-08
SP_PIR_KEYWORDS	protein biosynthesis	6.16E-11	4.9845	8.51E-08
GOTERM_CC_FAT	GO:0031981~nuclear lumen	2.96E-10	2.2146	4.16E-07
GOTERM_BP_FAT	GO:0016071~mRNA metabolic process	2.21E-09	3.1351	3.83E-06
GOTERM_BP_FAT	GO:0006396~RNA processing	2.44E-09	2.6936	4.23E-06
SP_PIR_KEYWORDS	ribosomal protein	2.49E-09	4.0649	3.43E-06
GOTERM_MF_FAT	GO:0003735~structural constituent of ribosome	2.67E-09	4.3307	3.95E-06
GOTERM_CC_FAT	GO:0005840~ribosome	6.29E-09	3.8731	8.83E-06
KEGG_PATHWAY	mmu03010:Ribosome	6.33E-09	5.0765	7.51E-06
GOTERM_BP_FAT	GO:0006397~mRNA processing	9.75E-09	3.2231	1.69E-05
GOTERM_MF_FAT	GO:0000166~nucleotide binding	2.55E-08	1.5817	3.77E-05
GOTERM_CC_FAT	GO:0043228~non-membrane-bounded organelle	4.33E-08	1.6361	6.08E-05

GOTERM_CC_FAT	GO:0043232~intracellular non-membrane-bounded organelle	4.33E-08	1.6361	6.08E-05
GOTERM_CC_FAT	GO:0005739~mitochondrion	7.96E-08	1.7917	1.12E-04
SP_PIR_KEYWORDS	mrna processing	1.04E-07	3.2199	1.44E-04
SP_PIR_KEYWORDS	methylation	2.52E-07	3.2556	3.48E-04
SP_PIR_KEYWORDS	transcription regulation	5.07E-07	1.6719	7.00E-04
SP_PIR_KEYWORDS	repressor	6.81E-07	2.5217	9.41E-04
GOTERM_CC_FAT	GO:0031967~organelle envelope	9.62E-07	2.2442	1.35E-03
GOTERM_BP_FAT	GO:0008380~RNA splicing	1.06E-06	3.1828	1.84E-03
GOTERM_CC_FAT	GO:0019866~organelle inner membrane	1.06E-06	2.7365	1.49E-03
GOTERM_CC_FAT	GO:0031975~envelope	1.08E-06	2.2359	1.52E-03
GOTERM_CC_FAT	GO:0044429~mitochondrial part	1.11E-06	2.2601	1.56E-03
SP_PIR_KEYWORDS	ribosome	1.11E-06	10.4274	1.53E-03
GOTERM_CC_FAT	GO:0005681~spliceosome	2.29E-06	3.9980	3.22E-03
SP_PIR_KEYWORDS	mrna splicing	2.98E-06	3.2089	4.12E-03
GOTERM_MF_FAT	GO:0008134~transcription factor binding	5.46E-06	2.6339	8.08E-03
GOTERM_CC_FAT	GO:0005654~nucleoplasm	6.12E-06	2.0691	8.59E-03
GOTERM_CC_FAT	GO:0031090~organelle membrane	8.66E-06	1.8724	1.22E-02
INTERPRO	IPR012677:Nucleotide-binding, alpha-beta plait	1.10E-05	2.9646	1.77E-02
SP_PIR_KEYWORDS	mitochondrion	1.49E-05	1.8552	2.06E-02
SP_PIR_KEYWORDS	nucleotide-binding	1.54E-05	1.5521	2.13E-02
GOTERM_MF_FAT	GO:0005198~structural molecule activity	2.22E-05	2.1507	3.28E-02

GO - hypo in ESC

Category	Term	PValue	Fold Enrichment	FDR
SP_PIR_KEYWORDS	acetylation	1.17E-194	2.7579	1.69E-191
SP_PIR_KEYWORDS	phosphoprotein	2.08E-97	1.5810	3.01E-94
SP_PIR_KEYWORDS	ribonucleoprotein	4.43E-64	4.5198	6.42E-61
GOTERM_CC_FAT	GO:0030529~ribonucleoprotein complex	4.00E-57	3.2816	5.87E-54
SP_PIR_KEYWORDS	cytoplasm	1.59E-56	1.7497	2.31E-53
GOTERM_MF_FAT	GO:0003735~structural constituent of ribosome	2.96E-55	5.3845	4.78E-52
GOTERM_BP_FAT	GO:0006412~translation	4.05E-55	3.8094	7.37E-52
SP_PIR_KEYWORDS	ribosomal protein	9.99E-51	4.8980	1.45E-47
GOTERM_CC_FAT	GO:0005840~ribosome	2.22E-47	4.4366	3.25E-44
KEGG_PATHWAY	mmu03010:Ribosome	5.12E-47	5.3904	6.29E-44
GOTERM_CC_FAT	GO:0005739~mitochondrion	3.70E-43	2.0276	5.43E-40
GOTERM_BP_FAT	GO:0006091~generation of precursor metabolites and energy	6.34E-41	3.6582	1.15E-37
SP_PIR_KEYWORDS	mitochondrion	4.38E-38	2.3708	6.35E-35
GOTERM_CC_FAT	GO:0044429~mitochondrial part	2.56E-37	2.6547	3.76E-34
GOTERM_CC_FAT	GO:0031090~organelle membrane	3.75E-33	2.1928	5.51E-30
GOTERM_CC_FAT	GO:0031966~mitochondrial membrane	2.93E-30	2.8032	4.31E-27
KEGG_PATHWAY	mmu00190:Oxidative phosphorylation	2.45E-29	3.7389	3.01E-26
SP_PIR_KEYWORDS	protein biosynthesis	4.63E-29	4.2364	6.71E-26
GOTERM_CC_FAT	GO:0005740~mitochondrial envelope	4.89E-29	2.6983	7.17E-26
GOTERM_CC_FAT	GO:0019866~organelle inner membrane	8.03E-29	2.9306	1.18E-25
SP_PIR_KEYWORDS	mitochondrion inner membrane	2.85E-28	3.7094	4.13E-25
GOTERM_CC_FAT	GO:0005743~mitochondrial inner membrane	5.43E-28	2.9570	7.98E-25
GOTERM_CC_FAT	GO:0031967~organelle envelope	5.79E-28	2.3734	8.50E-25
GOTERM_CC_FAT	GO:0031975~envelope	9.18E-28	2.3647	1.35E-24
GOTERM_BP_FAT	GO:0046907~intracellular transport	8.65E-26	2.5082	1.57E-22
KEGG_PATHWAY	mmu05016:Huntington's disease	1.63E-25	3.0700	2.00E-22
GOTERM_MF_FAT	GO:0000166~nucleotide binding	9.70E-25	1.5555	1.57E-21
GOTERM_CC_FAT	GO:0005829~cytosol	1.14E-24	2.2634	1.67E-21
GOTERM_CC_FAT	GO:0033279~ribosomal subunit	2.87E-24	5.3284	4.22E-21
SP_PIR_KEYWORDS	respiratory chain	3.34E-23	5.5404	4.84E-20
SP_PIR_KEYWORDS	rna-binding	3.75E-23	2.3608	5.44E-20

GOTERM_MF_FAT	GO:0003723~RNA binding	7.88E-23	2.0877	1.27E-19
SP_PIR_KEYWORDS	transit peptide	2.24E-22	2.3802	3.24E-19
KEGG_PATHWAY	mmu05012:Parkinson's disease	8.26E-22	3.2749	1.02E-18
GOTERM_CC_FAT	GO:0070469~respiratory chain	2.45E-21	5.0497	3.59E-18
GOTERM_CC_FAT	GO:0031974~membrane-enclosed lumen	2.57E-21	1.7374	3.77E-18
GOTERM_CC_FAT	GO:0043233~organelle lumen	4.41E-21	1.7474	6.47E-18
GOTERM_MF_FAT	GO:0005198~structural molecule activity	7.89E-21	2.3028	1.27E-17
GOTERM_CC_FAT	GO:0070013~intracellular organelle lumen	1.37E-20	1.7382	2.01E-17
SP_PIR_KEYWORDS	ribosome	1.52E-19	8.1787	2.20E-16
SP_PIR_KEYWORDS	electron transport	1.68E-19	4.0893	2.44E-16
GOTERM_CC_FAT	GO:0043232~intracellular non-membrane-bounded organelle	3.54E-19	1.5109	5.19E-16
GOTERM_CC_FAT	GO:0043228~non-membrane-bounded organelle	3.54E-19	1.5109	5.19E-16
GOTERM_BP_FAT	GO:0008104~protein localization	5.07E-19	1.9072	9.22E-16
SP_PIR_KEYWORDS	nucleotide-binding	5.54E-19	1.5946	8.03E-16
KEGG_PATHWAY	mmu05010:Alzheimer's disease	2.77E-18	2.7053	3.40E-15
UP_SEQ_FEATURE	transit peptide:Mitochondrion	3.10E-18	2.1451	5.84E-15
GOTERM_CC_FAT	GO:0042470~melanosome	3.94E-18	4.1374	5.79E-15
GOTERM_CC_FAT	GO:0048770~pigment granule	3.94E-18	4.1374	5.79E-15
GOTERM_BP_FAT	GO:0022900~electron transport chain	7.12E-18	3.6636	1.30E-14
GOTERM_BP_FAT	GO:0015031~protein transport	7.20E-18	1.9515	1.31E-14
GOTERM_BP_FAT	GO:0045184~establishment of protein localization	1.54E-17	1.9366	2.80E-14
GOTERM_BP_FAT	GO:0030163~protein catabolic process	4.83E-16	1.9869	8.10E-13
SP_PIR_KEYWORDS	transport	8.41E-16	1.5514	1.29E-12
GOTERM_BP_FAT	GO:0009057~macromolecule catabolic process	1.31E-15	1.8822	2.42E-12
SP_PIR_KEYWORDS	proteasome	2.56E-15	4.8466	3.70E-12
GOTERM_MF_FAT	GO:0032553~ribonucleotide binding	3.88E-15	1.4780	6.27E-12
GOTERM_MF_FAT	GO:0032555~purine ribonucleotide binding	3.88E-15	1.4780	6.27E-12
SP_PIR_KEYWORDS	ubl conjugation	4.12E-15	2.0134	5.95E-12
GOTERM_BP_FAT	GO:0034613~cellular protein localization	6.67E-15	2.3488	1.21E-11
GOTERM_MF_FAT	GO:0017076~purine nucleotide binding	7.04E-15	1.4613	1.13E-11
SP_PIR_KEYWORDS	protein transport	9.06E-15	2.0757	1.32E-11
GOTERM_BP_FAT	GO:0070727~cellular macromolecule localization	1.03E-14	2.3332	1.88E-11
GOTERM_MF_FAT	GO:0015077~monovalent	1.40E-14	3.7565	2.26E-11

	inorganic cation transmembrane transporter activity			
GOTERM_CC_FAT	GO:0000502~proteasome complex	1.51E-14	4.4465	2.22E-11
GOTERM_BP_FAT	GO:0006457~protein folding	2.39E-14	3.1688	4.34E-11
GOTERM_BP_FAT	GO:0044257~cellular protein catabolic process	2.50E-14	1.9396	4.55E-11
GOTERM_BP_FAT	GO:0006886~intracellular protein transport	3.17E-14	2.3730	5.78E-11
GOTERM_CC_FAT	GO:0016023~cytoplasmic membrane-bounded vesicle	3.35E-14	2.0764	4.92E-11
GOTERM_BP_FAT	GO:0051603~proteolysis involved in cellular protein catabolic process	3.69E-14	1.9358	6.71E-11
GOTERM_CC_FAT	GO:0031988~membrane-bounded vesicle	3.76E-14	2.0654	5.51E-11
GOTERM_CC_FAT	GO:0031982~vesicle	3.79E-14	1.9425	5.56E-11
GOTERM_CC_FAT	GO:0031410~cytoplasmic vesicle	3.95E-14	1.9538	5.78E-11
GOTERM_MF_FAT	GO:0015078~hydrogen ion transmembrane transporter activity	4.67E-14	3.7912	7.53E-11
GOTERM_BP_FAT	GO:0044265~cellular macromolecule catabolic process	9.06E-14	1.8529	1.65E-10
GOTERM_BP_FAT	GO:0043632~modification-dependent macromolecule catabolic process	1.22E-13	1.9416	2.22E-10
GOTERM_BP_FAT	GO:0019941~modification-dependent protein catabolic process	1.22E-13	1.9416	2.22E-10
SP_PIR_KEYWORDS	mrna splicing	1.40E-13	2.6977	2.02E-10
GOTERM_BP_FAT	GO:0008380~RNA splicing	2.07E-13	2.5910	3.77E-10
GOTERM_BP_FAT	GO:0006119~oxidative phosphorylation	2.26E-13	4.3681	4.11E-10
SP_PIR_KEYWORDS	ubl conjugation pathway	3.30E-13	1.9771	4.79E-10
SP_PIR_KEYWORDS	atp-binding	3.42E-13	1.5569	4.96E-10
GOTERM_BP_FAT	GO:0006397~mRNA processing	3.64E-13	2.3492	6.62E-10
SP_PIR_KEYWORDS	mrna processing	4.35E-13	2.4536	6.31E-10
GOTERM_CC_FAT	GO:0016469~proton-transporting two-sector ATPase complex	1.12E-12	4.8379	1.65E-09
GOTERM_CC_FAT	GO:0015934~large ribosomal subunit	1.14E-12	5.0096	1.68E-09
SP_PIR_KEYWORDS	isopeptide bond	1.55E-12	2.3030	2.25E-09
GOTERM_BP_FAT	GO:0015986~ATP synthesis coupled proton transport	2.06E-12	5.1184	3.75E-09
GOTERM_BP_FAT	GO:0015985~energy coupled proton transport, down electrochemical gradient	2.06E-12	5.1184	3.75E-09
GOTERM_CC_FAT	GO:0044445~cytosolic part	2.29E-12	3.9695	3.36E-09
GOTERM_BP_FAT	GO:0016071~mRNA metabolic process	2.38E-12	2.1948	4.34E-09

SP_PIR_KEYWORDS	Hydrogen ion transport	3.58E-12	4.7254	5.19E-09
GOTERM_CC_FAT	GO:0015935~small ribosomal subunit	3.89E-12	5.6591	5.71E-09
GOTERM_MF_FAT	GO:0051082~unfolded protein binding	7.42E-12	3.7579	1.20E-08
KEGG_PATHWAY	mmu03050:Proteasome	8.75E-12	3.8949	1.08E-08
SP_PIR_KEYWORDS	nucleus	1.42E-11	1.2650	2.05E-08
GOTERM_BP_FAT	GO:0009150~purine ribonucleotide metabolic process	4.41E-11	2.9176	8.02E-08
GOTERM_MF_FAT	GO:0032559~adenyl ribonucleotide binding	6.22E-11	1.4468	1.01E-07
GOTERM_BP_FAT	GO:0009152~purine ribonucleotide biosynthetic process	6.24E-11	3.0236	1.14E-07
GOTERM_BP_FAT	GO:0034220~ion transmembrane transport	7.98E-11	4.5090	1.45E-07
GOTERM_MF_FAT	GO:0022890~inorganic cation transmembrane transporter activity	9.24E-11	2.8244	1.49E-07
GOTERM_MF_FAT	GO:0030554~adenyl nucleotide binding	1.03E-10	1.4281	1.66E-07
KEGG_PATHWAY	mmu00020:Citrate cycle (TCA cycle)	1.13E-10	4.4798	1.39E-07
GOTERM_MF_FAT	GO:0005524~ATP binding	1.28E-10	1.4418	2.07E-07
UP_SEQ_FEATURE	cross-link:Glycyl lysine isopeptide (Lys-Gly) (interchain with G-Cter in ubiquitin)	1.44E-10	2.4508	2.71E-07
SP_PIR_KEYWORDS	methylation	1.52E-10	2.3315	2.20E-07
GOTERM_MF_FAT	GO:0001883~purine nucleoside binding	1.60E-10	1.4212	2.59E-07
GOTERM_BP_FAT	GO:0009260~ribonucleotide biosynthetic process	2.30E-10	2.9146	4.19E-07
GOTERM_BP_FAT	GO:0009259~ribonucleotide metabolic process	2.73E-10	2.7776	4.96E-07
GOTERM_BP_FAT	GO:0016192~vesicle-mediated transport	3.08E-10	1.8288	5.60E-07
GOTERM_BP_FAT	GO:0006396~RNA processing	3.09E-10	1.8599	5.63E-07
GOTERM_MF_FAT	GO:0001882~nucleoside binding	3.23E-10	1.4121	5.21E-07
GOTERM_BP_FAT	GO:0006099~tricarboxylic acid cycle	4.20E-10	5.8323	7.64E-07
GOTERM_CC_FAT	GO:0005759~mitochondrial matrix	4.24E-10	2.4931	6.23E-07
GOTERM_CC_FAT	GO:0031980~mitochondrial lumen	4.24E-10	2.4931	6.23E-07
INTERPRO	IPR012677:Nucleotide-binding, alpha-beta plait	4.28E-10	2.3349	7.61E-07
SP_PIR_KEYWORDS	Chaperone	4.69E-10	2.6172	6.80E-07
GOTERM_BP_FAT	GO:0019320~hexose catabolic process	4.78E-10	3.9454	8.70E-07
GOTERM_BP_FAT	GO:0006007~glucose catabolic process	4.78E-10	3.9454	8.70E-07

GOTERM_BP_FAT	GO:0015992~proton transport	4.78E-10	3.9454	8.70E-07
GOTERM_CC_FAT	GO:0022626~cytosolic ribosome	5.50E-10	6.5125	8.07E-07
GOTERM_CC_FAT	GO:0031981~nuclear lumen	6.26E-10	1.5577	9.18E-07
GOTERM_BP_FAT	GO:0044275~cellular carbohydrate catabolic process	6.34E-10	3.6824	1.15E-06
SP_PIR_KEYWORDS	viral nucleoprotein	7.17E-10	5.4524	1.04E-06
GOTERM_BP_FAT	GO:0006818~hydrogen transport	7.96E-10	3.8710	1.45E-06
SMART	SM00360:RRM	8.97E-10	2.3716	1.23E-06
SP_PIR_KEYWORDS	tricarboxylic acid cycle	1.02E-09	6.4568	1.48E-06
GOTERM_BP_FAT	GO:0046356~acetyl-CoA catabolic process	1.11E-09	5.5893	2.02E-06
GOTERM_BP_FAT	GO:0006006~glucose metabolic process	1.30E-09	2.5927	2.36E-06
GOTERM_BP_FAT	GO:0046365~monosaccharide catabolic process	1.30E-09	3.7993	2.37E-06
KEGG_PATHWAY	mmu03040:Spliceosome	1.41E-09	2.4435	1.73E-06
GOTERM_BP_FAT	GO:0009142~nucleoside triphosphate biosynthetic process	1.53E-09	2.9902	2.79E-06
GOTERM_BP_FAT	GO:0006096~glycolysis	2.06E-09	4.1247	3.75E-06
GOTERM_BP_FAT	GO:0006084~acetyl-CoA metabolic process	2.60E-09	4.8363	4.73E-06
GOTERM_MF_FAT	GO:0008135~translation factor activity, nucleic acid binding	3.04E-09	2.9282	4.91E-06
GOTERM_BP_FAT	GO:0009201~ribonucleoside triphosphate biosynthetic process	3.31E-09	2.9697	6.03E-06
GOTERM_BP_FAT	GO:0009206~purine ribonucleoside triphosphate biosynthetic process	3.31E-09	2.9697	6.03E-06
GOTERM_CC_FAT	GO:0033178~proton-transporting two-sector ATPase complex, catalytic domain	3.38E-09	6.4359	4.96E-06
GOTERM_CC_FAT	GO:0005681~spliceosome	3.57E-09	2.6470	5.23E-06
SP_PIR_KEYWORDS	endoplasmic reticulum	3.89E-09	1.6406	5.64E-06
GOTERM_BP_FAT	GO:0015980~energy derivation by oxidation of organic compounds	4.00E-09	2.8987	7.28E-06
SP_PIR_KEYWORDS	er-golgi transport	4.41E-09	3.2093	6.38E-06
GOTERM_BP_FAT	GO:0009145~purine nucleoside triphosphate biosynthetic process	4.56E-09	2.9381	8.30E-06
GOTERM_MF_FAT	GO:0046961~proton-transporting ATPase activity, rotational mechanism	4.60E-09	6.9084	7.42E-06
SP_PIR_KEYWORDS	Spliceosome	4.96E-09	2.7999	7.19E-06
SP_PIR_KEYWORDS	ligase	5.15E-09	2.0306	7.46E-06
GOTERM_BP_FAT	GO:0051187~cofactor catabolic process	5.24E-09	4.6852	9.53E-06
GOTERM_BP_FAT	GO:0009109~coenzyme catabolic process	5.93E-09	4.8978	1.08E-05
GOTERM_BP_FAT	GO:0006754~ATP biosynthetic	6.39E-09	3.0793	1.16E-05

	process			
GOTERM_MF_FAT	GO:0003743~translation initiation factor activity	6.66E-09	3.4713	1.08E-05
GOTERM_CC_FAT	GO:0012505~endomembrane system	6.79E-09	1.6945	9.96E-06
GOTERM_BP_FAT	GO:0009205~purine ribonucleoside triphosphate metabolic process	9.91E-09	2.8126	1.80E-05
GOTERM_BP_FAT	GO:0045333~cellular respiration	1.24E-08	3.4773	2.26E-05
GOTERM_BP_FAT	GO:0009199~ribonucleoside triphosphate metabolic process	1.33E-08	2.7850	2.42E-05
GOTERM_BP_FAT	GO:0009060~aerobic respiration	1.35E-08	4.9683	2.46E-05
INTERPRO	IPR000504:RNA recognition motif, RNP-1	2.08E-08	2.1895	3.70E-05
GOTERM_BP_FAT	GO:0046034~ATP metabolic process	2.08E-08	2.8933	3.79E-05
GOTERM_BP_FAT	GO:0006164~purine nucleotide biosynthetic process	2.18E-08	2.4767	3.97E-05
SP_PIR_KEYWORDS	molecular chaperone	2.27E-08	7.0103	3.29E-05
GOTERM_BP_FAT	GO:0006413~translational initiation	2.61E-08	4.1531	4.75E-05
GOTERM_MF_FAT	GO:0019829~cation-transporting ATPase activity	2.67E-08	5.1016	4.31E-05
GOTERM_BP_FAT	GO:0046164~alcohol catabolic process	2.70E-08	3.2777	4.92E-05
GOTERM_MF_FAT	GO:0019787~small conjugating protein ligase activity	3.05E-08	2.5508	4.93E-05
SP_PIR_KEYWORDS	Initiation factor	3.33E-08	3.5689	4.82E-05
GOTERM_BP_FAT	GO:0019318~hexose metabolic process	3.34E-08	2.2879	6.09E-05
GOTERM_BP_FAT	GO:0009141~nucleoside triphosphate metabolic process	3.64E-08	2.6074	6.63E-05
GOTERM_CC_FAT	GO:0005794~Golgi apparatus	4.06E-08	1.5699	5.95E-05
GOTERM_BP_FAT	GO:0009144~purine nucleoside triphosphate metabolic process	4.06E-08	2.6799	7.39E-05
GOTERM_BP_FAT	GO:0006163~purine nucleotide metabolic process	4.29E-08	2.3179	7.81E-05
GOTERM_CC_FAT	GO:0048475~coated membrane	5.06E-08	3.3685	7.43E-05
GOTERM_CC_FAT	GO:0030117~membrane coat	5.06E-08	3.3685	7.43E-05
GOTERM_BP_FAT	GO:0016052~carbohydrate catabolic process	7.97E-08	2.9225	1.45E-04
GOTERM_CC_FAT	GO:0005783~endoplasmic reticulum	8.77E-08	1.4921	1.29E-04
SP_PIR_KEYWORDS	glycolysis	1.14E-07	3.8946	1.65E-04
GOTERM_CC_FAT	GO:0005768~endosome	1.84E-07	1.9537	2.70E-04
GOTERM_BP_FAT	GO:0034404~nucleobase, nucleoside and nucleotide biosynthetic process	2.30E-07	2.1601	4.19E-04
GOTERM_BP_FAT	GO:0034654~nucleobase, nucleoside, nucleotide and nucleic acid biosynthetic process	2.30E-07	2.1601	4.19E-04

SMART	SM00320:WD40	2.49E-07	2.0106	3.41E-04
GOTERM_MF_FAT	GO:0016879~ligase activity, forming carbon-nitrogen bonds	3.00E-07	2.1064	4.84E-04
GOTERM_MF_FAT	GO:0008092~cytoskeletal protein binding	3.06E-07	1.7136	4.94E-04
GOTERM_MF_FAT	GO:0046933~hydrogen ion transporting ATP synthase activity, rotational mechanism	3.26E-07	7.2466	5.26E-04
GOTERM_CC_FAT	GO:0045259~proton-transporting ATP synthase complex	3.89E-07	5.3471	5.71E-04
GOTERM_MF_FAT	GO:0042625~ATPase activity, coupled to transmembrane movement of ions	3.92E-07	3.0036	6.33E-04
SP_PIR_KEYWORDS	golgi apparatus	3.95E-07	1.6121	5.72E-04
GOTERM_CC_FAT	GO:0009898~internal side of plasma membrane	4.85E-07	1.8516	7.12E-04
GOTERM_BP_FAT	GO:0005996~monosaccharide metabolic process	7.33E-07	2.0657	1.33E-03
GOTERM_MF_FAT	GO:0004842~ubiquitin-protein ligase activity	7.35E-07	2.5293	1.19E-03
GOTERM_BP_FAT	GO:0006605~protein targeting	7.46E-07	2.3139	1.36E-03
INTERPRO	IPR019775:WD40 repeat, conserved site	7.50E-07	1.9276	1.33E-03
GOTERM_CC_FAT	GO:0005762~mitochondrial large ribosomal subunit	7.68E-07	4.7570	1.13E-03
GOTERM_CC_FAT	GO:0000315~organellar large ribosomal subunit	7.68E-07	4.7570	1.13E-03
GOTERM_MF_FAT	GO:0016881~acid-amino acid ligase activity	7.72E-07	2.1609	1.25E-03
GOTERM_MF_FAT	GO:0043021~ribonucleoprotein binding	8.28E-07	3.9856	1.34E-03
GOTERM_CC_FAT	GO:0005761~mitochondrial ribosome	8.32E-07	3.6216	1.22E-03
GOTERM_CC_FAT	GO:0000313~organellar ribosome	8.32E-07	3.6216	1.22E-03
SP_PIR_KEYWORDS	endosome	8.38E-07	2.0653	1.21E-03
GOTERM_BP_FAT	GO:0045454~cell redox homeostasis	8.41E-07	3.0545	1.53E-03
SP_PIR_KEYWORDS	wd repeat	1.03E-06	1.8969	1.50E-03
GOTERM_MF_FAT	GO:0042623~ATPase activity, coupled	1.05E-06	1.9660	1.69E-03
GOTERM_BP_FAT	GO:0019725~cellular homeostasis	1.10E-06	1.7484	2.01E-03
GOTERM_MF_FAT	GO:0019843~rRNA binding	1.18E-06	4.6499	1.90E-03
GOTERM_CC_FAT	GO:0030135~coated vesicle	1.19E-06	2.3319	1.74E-03
GOTERM_BP_FAT	GO:0034621~cellular macromolecular complex subunit organization	1.31E-06	1.9002	2.39E-03
GOTERM_BP_FAT	GO:0034622~cellular macromolecular complex assembly	1.36E-06	1.9636	2.48E-03
GOTERM_BP_FAT	GO:0009165~nucleotide	1.62E-06	2.0861	2.95E-03

	biosynthetic process			
INTERPRO	IPR019781:WD40 repeat, subgroup	1.86E-06	1.9399	3.31E-03
GOTERM_CC_FAT	GO:0031252~cell leading edge	2.28E-06	2.3724	3.35E-03
GOTERM_MF_FAT	GO:0016887~ATPase activity	2.35E-06	1.8155	3.79E-03
SP_PIR_KEYWORDS	RNA binding	2.70E-06	6.2913	3.91E-03
SP_PIR_KEYWORDS	gtp-binding	2.75E-06	1.7768	3.99E-03
GOTERM_BP_FAT	GO:0006417~regulation of translation	3.59E-06	2.4462	6.53E-03
GOTERM_BP_FAT	GO:0030705~cytoskeleton-dependent intracellular transport	3.60E-06	3.8259	6.55E-03
INTERPRO	IPR017986:WD40 repeat, region	3.61E-06	1.9268	6.43E-03
UP_SEQ_FEATURE	domain:PCI	3.62E-06	4.9016	6.82E-03
INTERPRO	IPR001680:WD40 repeat	4.10E-06	1.8562	7.30E-03
GOTERM_BP_FAT	GO:0010608~posttranscriptional regulation of gene expression	4.83E-06	2.1327	8.78E-03
GOTERM_CC_FAT	GO:0030027~lamellipodium	5.42E-06	2.8531	7.96E-03
GOTERM_CC_FAT	GO:0005654~nucleoplasm	5.51E-06	1.5004	8.09E-03
SP_PIR_KEYWORDS	blocked amino end	5.53E-06	3.9574	8.01E-03
SP_PIR_KEYWORDS	nitration	5.69E-06	3.7388	8.24E-03
GOTERM_CC_FAT	GO:0016585~chromatin remodeling complex	5.70E-06	3.1260	8.36E-03
INTERPRO	IPR019782:WD40 repeat 2	6.07E-06	1.9366	1.08E-02
GOTERM_CC_FAT	GO:0008287~protein serine/threonine phosphatase complex	6.40E-06	3.6776	9.39E-03
KEGG_PATHWAY	mmu04120:Ubiquitin mediated proteolysis	6.62E-06	1.9958	8.13E-03
GOTERM_BP_FAT	GO:0043623~cellular protein complex assembly	6.86E-06	2.3380	1.25E-02
SP_PIR_KEYWORDS	ubiquinone	7.05E-06	4.4301	1.02E-02
GOTERM_BP_FAT	GO:0055114~oxidation reduction	7.16E-06	1.4678	1.30E-02
GOTERM_BP_FAT	GO:0032446~protein modification by small protein conjugation	8.08E-06	2.5970	1.47E-02
GOTERM_BP_FAT	GO:0070647~protein modification by small protein conjugation or removal	8.62E-06	2.3521	1.57E-02
GOTERM_MF_FAT	GO:0050136~NADH dehydrogenase (quinone) activity	9.15E-06	4.3177	1.48E-02
GOTERM_MF_FAT	GO:0003954~NADH dehydrogenase activity	9.15E-06	4.3177	1.48E-02
GOTERM_MF_FAT	GO:0008137~NADH dehydrogenase (ubiquinone) activity	9.15E-06	4.3177	1.48E-02
GOTERM_BP_FAT	GO:0006511~ubiquitin-dependent protein catabolic process	9.29E-06	2.1266	1.69E-02
GOTERM_MF_FAT	GO:0008565~protein transporter activity	9.33E-06	2.7778	1.51E-02

GOTERM_BP_FAT	GO:0006796~phosphate metabolic process	1.19E-05	1.3941	2.17E-02
GOTERM_BP_FAT	GO:0006793~phosphorus metabolic process	1.19E-05	1.3941	2.17E-02
GOTERM_BP_FAT	GO:0033043~regulation of organelle organization	1.34E-05	2.0496	2.43E-02
GOTERM_CC_FAT	GO:0030120~vesicle coat	1.35E-05	3.9075	1.98E-02
SMART	SM00088:PINT	1.40E-05	5.6115	1.91E-02
SP_PIR_KEYWORDS	mitochondrion outer membrane	1.40E-05	2.7262	2.03E-02
UP_SEQ_FEATURE	nucleotide phosphate-binding region:GTP	1.45E-05	1.7174	2.73E-02
GOTERM_CC_FAT	GO:0005905~coated pit	1.47E-05	3.6633	2.15E-02
GOTERM_CC_FAT	GO:0000118~histone deacetylase complex	1.47E-05	3.6633	2.15E-02
SP_PIR_KEYWORDS	rrna-binding	1.50E-05	5.4524	2.17E-02
GOTERM_BP_FAT	GO:0007264~small GTPase mediated signal transduction	1.52E-05	1.7739	2.77E-02
GOTERM_CC_FAT	GO:0030662~coated vesicle membrane	1.53E-05	2.9491	2.24E-02
GOTERM_MF_FAT	GO:0003729~mRNA binding	1.57E-05	2.9523	2.53E-02
SP_PIR_KEYWORDS	kinase	1.64E-05	1.4460	2.38E-02
GOTERM_CC_FAT	GO:0033176~proton-transporting V-type ATPase complex	1.88E-05	4.7758	2.75E-02
INTERPRO	IPR005225:Small GTP-binding protein	2.09E-05	2.0217	3.72E-02
GOTERM_CC_FAT	GO:0005852~eukaryotic translation initiation factor 3 complex	2.16E-05	5.8613	3.17E-02
GOTERM_MF_FAT	GO:0016651~oxidoreductase activity, acting on NADH or NADPH	2.49E-05	2.9697	4.01E-02
GOTERM_CC_FAT	GO:0005832~chaperonin-containing T-complex	2.70E-05	7.8150	3.97E-02
SP_PIR_KEYWORDS	Redox-active center	2.73E-05	3.3553	3.96E-02
SP_PIR_KEYWORDS	Aminoacyl-tRNA synthetase	2.73E-05	3.3553	3.96E-02
GOTERM_BP_FAT	GO:0043933~macromolecular complex subunit organization	2.74E-05	1.6126	4.98E-02
INTERPRO	IPR000717:Proteasome component region PCI	2.74E-05	5.1805	4.87E-02
PIR_SUPERFAMILY	PIRSF002584:molecular chaperone t-complex-type	2.98E-05	6.3106	4.81E-02
GOTERM_CC_FAT	GO:0000151~ubiquitin ligase complex	3.14E-05	2.7353	4.61E-02
GOTERM_CC_FAT	GO:0030118~clathrin coat	3.38E-05	3.4478	4.96E-02

SI_Sheets_S3

DMS 10dpi

seqnames	start	end	log2.RR	pvalue	padj	GENE_SYMBOL
chr4	1193043	1193043	1.2696E+00	1.42E-250	2.74E-246	LOC100130872
chr4	1193063	1193063	1.0646E+00	8.32E-238	8.03E-234	LOC100130872
chr4	1193053	1193053	1.1573E+00	9.58E-233	6.16E-229	LOC100130872
chr4	1193052	1193052	1.2227E+00	3.80E-228	1.84E-224	LOC100130872
chr4	1193072	1193072	1.1284E+00	2.76E-227	1.07E-223	LOC100130872
chr4	1193045	1193045	1.0930E+00	1.63E-226	5.25E-223	LOC100130872
chr4	1193050	1193050	1.0026E+00	9.09E-217	2.51E-213	LOC100130872
chr4	1193047	1193047	1.0592E+00	3.56E-207	8.60E-204	LOC100130872
chr4	1193049	1193049	8.7507E-01	6.70E-197	1.44E-193	LOC100130872
chr4	1193079	1193079	1.2792E+00	2.21E-196	4.26E-193	LOC100130872
chr4	1193080	1193080	1.3185E+00	2.29E-189	4.02E-186	LOC100130872
chr4	1193055	1193055	1.0996E+00	4.35E-177	7.00E-174	LOC100130872
chr4	1193040	1193040	1.1545E+00	2.72E-156	4.04E-153	LOC100130872
chr4	166263650	166263650	5.4550E-01	4.65E-88	6.41E-85	MSMO1
chr4	166263634	166263634	5.9566E-01	1.64E-83	2.11E-80	MSMO1
chr4	166263641	166263641	4.6154E-01	2.82E-74	3.40E-71	MSMO1
chr4	166263630	166263630	5.2636E-01	8.14E-71	9.24E-68	MSMO1
chr4	166263629	166263629	4.0942E-01	2.56E-58	2.75E-55	MSMO1
chr4	166263632	166263632	4.6409E-01	4.60E-56	4.67E-53	MSMO1
chr4	177252863	177252863	9.3232E-01	2.94E-54	2.84E-51	SPCS3
chr4	177252862	177252862	1.1693E+00	1.95E-53	1.79E-50	SPCS3
chr4	177252859	177252859	9.9318E-01	1.83E-52	1.61E-49	SPCS3
chrX	24095306	24095306	2.6993E-01	2.26E-48	1.90E-45	EIF2S3
chrX	24095305	24095305	2.4194E-01	2.13E-46	1.71E-43	EIF2S3
chrX	24095310	24095310	2.4629E-01	3.97E-46	3.06E-43	EIF2S3
chr4	177252860	177252860	8.8821E-01	2.91E-45	2.16E-42	SPCS3
chrX	24095317	24095317	4.2728E-01	2.80E-44	2.00E-41	EIF2S3
chrX	24095308	24095308	2.4997E-01	1.39E-43	9.59E-41	EIF2S3
chrX	24095309	24095309	2.4299E-01	6.77E-43	4.51E-40	EIF2S3
chr6	34845930	34845930	1.8722E+00	3.33E-38	2.14E-35	TAF11
chr6	34845926	34845926	1.8794E+00	4.24E-38	2.64E-35	TAF11
chr4	166263626	166263626	5.2192E-01	8.02E-38	4.84E-35	MSMO1
chr4	159630561	159630561	9.2519E-01	1.77E-37	1.04E-34	PPID
chr6	34845924	34845924	1.6540E+00	2.59E-37	1.47E-34	TAF11
chr4	177252856	177252856	8.5357E-01	8.41E-37	4.64E-34	SPCS3

chr6	34845937	34845937	2.4233E+00	9.88E-37	5.30E-34	TAF11
chr4	159630555	159630555	1.1063E+00	1.99E-36	1.04E-33	PPID
chr6	34845923	34845923	1.6412E+00	4.99E-36	2.54E-33	TAF11
chr4	177252880	177252880	1.2255E+00	9.22E-35	4.56E-32	SPCS3
chr14	93649148	93649148	9.2888E-01	1.21E-34	5.74E-32	MOAP1
chr14	93649152	93649152	9.6882E-01	1.22E-34	5.74E-32	MOAP1
chr14	93649155	93649155	9.5102E-01	4.17E-34	1.92E-31	MOAP1
chr4	177252893	177252893	9.6778E-01	4.83E-34	2.17E-31	SPCS3
chr4	159630547	159630547	9.9525E-01	1.61E-33	7.04E-31	PPID
chr4	177252896	177252896	1.0669E+00	3.23E-33	1.38E-30	SPCS3
chr4	177252894	177252894	1.1281E+00	3.35E-33	1.40E-30	SPCS3
chrX	24095302	24095302	2.2844E-01	7.64E-33	3.14E-30	EIF2S3
chr14	93649141	93649141	1.1583E+00	1.42E-32	5.70E-30	MOAP1
chr6	34845932	34845932	1.6055E+00	5.72E-32	2.26E-29	TAF11
chr6	34845935	34845935	1.6044E+00	5.88E-32	2.27E-29	TAF11
chr6	34845933	34845933	1.4180E+00	5.82E-31	2.20E-28	TAF11
chr19	24010197	24010197	3.0463E-02	2.90E-30	1.08E-27	RPSAP58
chr1	36929796	36929796	1.5017E+00	5.02E-30	1.83E-27	MRPS15
chr6	34845936	34845936	1.2641E+00	8.90E-30	3.18E-27	TAF11
chr4	177252853	177252853	1.2567E+00	2.72E-29	9.57E-27	SPCS3
chr4	177252854	177252854	8.3436E-01	6.03E-29	2.08E-26	SPCS3
chr4	1813547	1813547	5.3746E-01	1.01E-28	3.43E-26	LETM1
chr4	159630548	159630548	6.4426E-01	2.10E-28	6.99E-26	PPID
chr14	93649161	93649161	9.4407E-01	5.58E-28	1.83E-25	MOAP1
chr14	93649139	93649139	9.5637E-01	1.18E-27	3.81E-25	MOAP1
chr4	159630545	159630545	1.2882E+00	1.49E-27	4.71E-25	PPID
chr8	22878091	22878091	1.1032E+00	1.82E-27	5.66E-25	TNFRSF10B
chrX	24095300	24095300	2.5205E-01	2.33E-27	7.13E-25	EIF2S3
chr6	34845906	34845906	1.5231E+00	1.13E-26	3.42E-24	TAF11
chr1	36929785	36929785	1.7138E+00	3.26E-26	9.68E-24	MRPS15
chrX	24095336	24095336	8.2306E-01	5.13E-26	1.50E-23	EIF2S3
chr14	93649142	93649142	1.0066E+00	1.18E-25	3.39E-23	MOAP1
chrX	24095299	24095299	2.5543E-01	1.71E-25	4.78E-23	EIF2S3
chr11	8941469	8941469	7.7907E-01	1.71E-25	4.78E-23	AKIP1
chr12	2997810	2997810	8.3608E-01	2.14E-25	5.91E-23	RHNO1
chr1	36929808	36929808	1.1389E+00	2.60E-25	7.08E-23	MRPS15
chr14	93649136	93649136	1.0979E+00	6.37E-25	1.71E-22	MOAP1
chr8	22878103	22878103	1.1139E+00	9.98E-25	2.64E-22	TNFRSF10B
chr1	36929809	36929809	1.1195E+00	1.27E-24	3.32E-22	MRPS15
chr1	36929804	36929804	1.3816E+00	3.66E-24	9.42E-22	MRPS15
chr14	93649168	93649168	1.0345E+00	4.63E-24	1.18E-21	MOAP1

chr7	56148077	56148077	1.7341E-01	4.72E-24	1.18E-21	SUMF2
chr7	56148079	56148079	1.6855E-01	8.10E-24	1.99E-21	SUMF2
chr6	34845915	34845915	1.3988E+00	8.15E-24	1.99E-21	TAF11
chr11	8941308	8941308	5.2709E-01	1.30E-23	3.14E-21	AKIP1
chr11	6505467	6505467	9.3251E-01	1.49E-23	3.55E-21	TIMM10B
chr8	22878100	22878100	1.1764E+00	2.26E-23	5.31E-21	TNFRSF10B
chr12	2997814	2997814	6.4243E-01	3.17E-23	7.38E-21	RHNO1
chr8	22878102	22878102	1.0071E+00	3.61E-23	8.30E-21	TNFRSF10B
chr11	8941473	8941473	7.5632E-01	5.51E-23	1.25E-20	AKIP1
chr7	56148080	56148080	1.6563E-01	6.22E-23	1.40E-20	SUMF2
chr14	93649138	93649138	8.2664E-01	7.23E-23	1.61E-20	MOAP1
chr8	22878099	22878099	1.0150E+00	8.26E-23	1.81E-20	TNFRSF10B
chr14	93649169	93649169	1.0719E+00	8.38E-23	1.82E-20	MOAP1
chr4	4237880	4237880	1.5283E+00	1.20E-22	2.57E-20	TMEM128
chr17	57188015	57188015	4.0766E-01	1.57E-22	3.33E-20	SKA2
chr11	8941307	8941307	5.5842E-01	1.83E-22	3.84E-20	AKIP1
chr11	8941230	8941230	8.5651E-01	3.28E-22	6.80E-20	AKIP1
chr17	57188014	57188014	4.5292E-01	3.87E-22	7.95E-20	SKA2
chr12	2997808	2997808	7.3257E-01	5.64E-22	1.15E-19	RHNO1
chr7	56148073	56148073	1.6818E-01	7.32E-22	1.47E-19	SUMF2
chr12	2997807	2997807	7.3604E-01	1.12E-21	2.22E-19	RHNO1
chr11	8941477	8941477	7.2303E-01	1.13E-21	2.23E-19	AKIP1
chr4	4237884	4237884	1.6046E+00	1.80E-21	3.51E-19	TMEM128
chr6	34845909	34845909	1.3242E+00	1.90E-21	3.66E-19	TAF11
chr11	6505473	6505473	7.8791E-01	5.63E-21	1.08E-18	TIMM10B
chr11	6505472	6505472	5.9237E-01	5.76E-21	1.09E-18	TIMM10B
chr11	8941306	8941306	5.2515E-01	7.61E-21	1.43E-18	AKIP1
chr9	33046789	33046789	8.1053E-01	8.29E-21	1.54E-18	SMU1
chr11	8941304	8941304	4.5016E-01	9.97E-21	1.83E-18	AKIP1
chr1	36929787	36929787	9.8671E-01	1.55E-20	2.82E-18	MRPS15
chr3	10143193	10143193	3.6611E-01	1.75E-20	3.16E-18	FANCD2
chr11	8941470	8941470	5.8628E-01	2.37E-20	4.24E-18	AKIP1
chr1	36929810	36929810	1.4377E+00	2.58E-20	4.57E-18	MRPS15
chr1	3697433	3697433	8.5769E-01	6.31E-20	1.11E-17	LRRC47
chr9	33046786	33046786	8.3292E-01	6.77E-20	1.18E-17	SMU1
chr1	3697438	3697438	8.7067E-01	7.69E-20	1.33E-17	LRRC47
chr7	56148088	56148088	1.3531E-01	1.14E-19	1.95E-17	SUMF2
chr4	4237894	4237894	1.5393E+00	1.30E-19	2.20E-17	TMEM128
chr7	102277721	102277721	4.8937E-01	1.36E-19	2.28E-17	POLR2J2
chr7	56148071	56148071	1.6627E-01	1.41E-19	2.36E-17	SUMF2
chr9	33046785	33046785	8.0262E-01	1.45E-19	2.40E-17	SMU1

chr17	57188012	57188012	4.3694E-01	1.85E-19	3.02E-17	SKA2
chr7	56148070	56148070	1.6594E-01	2.05E-19	3.32E-17	SUMF2
chr12	2997813	2997813	6.1995E-01	2.28E-19	3.67E-17	RHNO1
chr3	10143190	10143190	3.9707E-01	2.35E-19	3.75E-17	FANCD2
chrX	24095249	24095249	2.8945E-01	2.59E-19	4.10E-17	EIF2S3
chr12	2997804	2997804	1.1549E+00	2.78E-19	4.37E-17	RHNO1
chr3	10143189	10143189	3.9342E-01	2.92E-19	4.55E-17	FANCD2
chr7	56148076	56148076	1.4129E-01	3.07E-19	4.75E-17	SUMF2
chr1	36929784	36929784	1.6919E+00	3.14E-19	4.81E-17	MRPS15
chr11	8941303	8941303	4.7739E-01	3.66E-19	5.57E-17	AKIP1
chr1	3697437	3697437	7.8978E-01	4.10E-19	6.18E-17	LRRC47
chr11	8941478	8941478	6.5029E-01	5.15E-19	7.71E-17	AKIP1
chr4	1813546	1813546	5.3354E-01	6.64E-19	9.86E-17	LETM1
chr11	8941472	8941472	5.2676E-01	8.15E-19	1.20E-16	AKIP1
chr9	33046787	33046787	7.5267E-01	9.33E-19	1.36E-16	SMU1
chr11	8941479	8941479	7.0887E-01	1.04E-18	1.51E-16	AKIP1
chr4	1813544	1813544	5.2434E-01	1.29E-18	1.86E-16	LETM1
chr12	2997818	2997818	6.5366E-01	1.30E-18	1.86E-16	RHNO1
chr1	3697434	3697434	7.5022E-01	2.10E-18	2.98E-16	LRRC47
chr9	33046778	33046778	6.3568E-01	2.94E-18	4.15E-16	SMU1
chr1	3697435	3697435	8.0399E-01	3.10E-18	4.33E-16	LRRC47
chr17	43002695	43002695	5.1223E-01	3.28E-18	4.55E-16	KIF18B
chr8	22878106	22878106	1.1282E+00	4.24E-18	5.85E-16	TNFRSF10B
chr11	6505441	6505441	8.1152E-01	4.76E-18	6.52E-16	TIMM10B
chr4	4237883	4237883	1.6802E+00	5.18E-18	7.04E-16	TMEM128
chr4	4237881	4237881	1.3273E+00	6.52E-18	8.80E-16	TMEM128
chr12	2997801	2997801	1.9155E+00	8.87E-18	1.19E-15	RHNO1
chr9	33046790	33046790	7.6312E-01	1.48E-17	1.98E-15	SMU1
chr17	38458731	38458731	4.1364E-01	1.51E-17	2.00E-15	CDC6
chr11	6505475	6505475	8.1417E-01	1.97E-17	2.58E-15	TIMM10B
chr1	3697441	3697441	9.2443E-01	3.26E-17	4.26E-15	LRRC47
chr3	10143184	10143184	3.5021E-01	3.59E-17	4.66E-15	FANCD2
chr11	8941475	8941475	5.2967E-01	7.90E-17	1.02E-14	AKIP1
chr4	4237876	4237876	1.8080E+00	1.25E-16	1.59E-14	TMEM128
chr11	6505443	6505443	7.1960E-01	1.39E-16	1.76E-14	TIMM10B
chr17	57188028	57188028	4.6837E-01	2.61E-16	3.29E-14	SKA2
chr14	93649170	93649170	1.0235E+00	2.83E-16	3.55E-14	MOAP1
chr8	22878108	22878108	1.1105E+00	3.52E-16	4.38E-14	TNFRSF10B
chr3	33184297	33184297	1.9300E+00	6.60E-16	8.17E-14	CRTAP
chr17	38458730	38458730	4.2460E-01	8.42E-16	1.04E-13	CDC6
chr3	10143192	10143192	3.4217E-01	8.95E-16	1.09E-13	FANCD2

chr12	2997817	2997817	6.6695E-01	1.66E-15	2.01E-13	RHNO1
chr16	8947812	8947812	1.8285E+00	1.97E-15	2.38E-13	CARHSP1
chr7	102277724	102277724	4.6239E-01	2.44E-15	2.93E-13	POLR2J2
chrX	24095337	24095337	5.9236E-01	2.55E-15	3.04E-13	EIF2S3
chr4	4237897	4237897	1.7114E+00	3.20E-15	3.80E-13	TMEM128
chr17	38458729	38458729	4.0552E-01	3.74E-15	4.40E-13	CDC6
chr19	17453208	17453208	8.1805E-01	4.52E-15	5.29E-13	GTPBP3
chr1	3806145	3806145	1.2202E+00	4.65E-15	5.40E-13	C1orf174
chr1	21070460	21070460	6.9447E-01	4.69E-15	5.42E-13	HP1BP3
chr17	43002710	43002710	5.0059E-01	4.81E-15	5.53E-13	KIF18B
chr11	6505439	6505439	6.0934E-01	5.40E-15	6.17E-13	TIMM10B
chr11	8941300	8941300	5.5863E-01	5.76E-15	6.54E-13	AKIP1
chr3	10143186	10143186	3.7880E-01	7.85E-15	8.87E-13	FANCD2
chr4	6718853	6718853	7.2640E-01	9.57E-15	1.07E-12	BLOC1S4
chr20	49577403	49577403	7.6002E-01	9.65E-15	1.08E-12	MOCS3
chr17	57188044	57188044	6.1479E-01	1.22E-14	1.35E-12	SKA2
chr11	8941298	8941298	6.1457E-01	1.31E-14	1.44E-12	AKIP1
chr16	8947838	8947838	1.6432E+00	1.68E-14	1.84E-12	CARHSP1
chrX	24095334	24095334	7.5329E-01	1.72E-14	1.88E-12	EIF2S3
chr16	8947837	8947837	1.4543E+00	2.72E-14	2.95E-12	CARHSP1
chr14	75598977	75598977	1.0383E+00	2.77E-14	2.98E-12	TMED10
chr4	6718857	6718857	9.1022E-01	3.10E-14	3.32E-12	BLOC1S4
chr20	49577404	49577404	8.6524E-01	3.56E-14	3.79E-12	MOCS3
chr3	10143183	10143183	4.0059E-01	4.42E-14	4.69E-12	FANCD2
chr17	43002692	43002692	3.8914E-01	5.33E-14	5.63E-12	KIF18B
chrX	24095332	24095332	7.8969E-01	7.18E-14	7.53E-12	EIF2S3
chr8	22878109	22878109	7.9919E-01	7.43E-14	7.75E-12	TNFRSF10B
chr17	43002708	43002708	4.9220E-01	1.10E-13	1.14E-11	KIF18B
chr11	6505438	6505438	5.9234E-01	1.17E-13	1.21E-11	TIMM10B
chr12	2997838	2997838	8.6330E-01	1.29E-13	1.32E-11	RHNO1
chr12	2997803	2997803	7.7257E-01	1.46E-13	1.49E-11	RHNO1
chr11	8941482	8941482	7.9981E-01	2.05E-13	2.08E-11	AKIP1
chr16	8947840	8947840	2.1851E+00	2.34E-13	2.36E-11	CARHSP1
chrX	24095241	24095241	2.7677E-01	2.63E-13	2.64E-11	EIF2S3
chrX	24095326	24095326	7.3304E-01	2.76E-13	2.76E-11	EIF2S3
chr7	56148068	56148068	1.5515E-01	2.82E-13	2.81E-11	SUMF2
chr20	49577412	49577412	6.2485E-01	3.18E-13	3.15E-11	MOCS3
chr17	57188045	57188045	5.4443E-01	3.30E-13	3.25E-11	SKA2
chr11	6505459	6505459	5.3604E-01	3.84E-13	3.76E-11	TIMM10B
chr17	38458760	38458760	3.7414E-01	4.03E-13	3.93E-11	CDC6
chr14	75598992	75598992	8.9817E-01	4.22E-13	4.09E-11	TMED10

chr4	6718856	6718856	8.1207E-01	4.60E-13	4.44E-11	BLOC1S4
chr20	49577400	49577400	7.0848E-01	4.64E-13	4.46E-11	MOCS3
chr4	6718848	6718848	7.1894E-01	4.76E-13	4.55E-11	BLOC1S4
chr1	3697444	3697444	7.5974E-01	4.86E-13	4.63E-11	LRRC47
chr4	6718847	6718847	8.1951E-01	5.08E-13	4.81E-11	BLOC1S4
chr5	174955061	174955061	1.5726E+00	6.52E-13	6.14E-11	SFXN1
chr4	6718851	6718851	6.2749E-01	7.09E-13	6.65E-11	BLOC1S4
chr19	17453217	17453217	7.0074E-01	7.22E-13	6.74E-11	GTPBP3
chr17	38458727	38458727	3.3511E-01	7.59E-13	7.05E-11	CDC6
chr4	6718843	6718843	8.2118E-01	7.68E-13	7.10E-11	BLOC1S4
chr20	49577425	49577425	9.6159E-01	7.88E-13	7.24E-11	MOCS3
chr11	65180591	65180591	6.1682E-01	8.27E-13	7.57E-11	FRMD8
chr17	29110536	29110536	9.8075E-01	8.79E-13	8.01E-11	CRLF3
chr3	33184301	33184301	1.9305E+00	9.15E-13	8.25E-11	CRTAP
chr12	2997825	2997825	8.6047E-01	9.11E-13	8.25E-11	RHNO1
chr4	6718835	6718835	7.7479E-01	9.72E-13	8.73E-11	BLOC1S4
chr7	56148067	56148067	1.5689E-01	1.28E-12	1.14E-10	SUMF2
chr20	49577401	49577401	6.5153E-01	1.32E-12	1.18E-10	MOCS3
chr16	8947820	8947820	8.3225E-01	1.97E-12	1.74E-10	CARHSP1
chr17	38458726	38458726	2.9363E-01	2.41E-12	2.11E-10	CDC6
chr1	3806153	3806153	7.4467E-01	2.41E-12	2.11E-10	C1orf174
chr12	2997796	2997796	8.2327E-01	2.89E-12	2.52E-10	RHNO1
chr20	49577421	49577421	1.0943E+00	4.41E-12	3.83E-10	MOCS3
chr7	56148066	56148066	2.0870E-01	4.63E-12	4.01E-10	SUMF2
chr1	21070455	21070455	7.4532E-01	4.66E-12	4.01E-10	HP1BP3
chr3	33184300	33184300	1.4973E+00	4.98E-12	4.28E-10	CRTAP
chr17	57188046	57188046	5.3409E-01	1.55E-11	1.32E-09	SKA2
chr9	116169961	116169961	7.7894E-01	1.59E-11	1.35E-09	POLE3
chr1	3697443	3697443	7.5383E-01	2.42E-11	2.05E-09	LRRC47
chr17	29110532	29110532	7.1724E-01	2.75E-11	2.31E-09	CRLF3
chr12	2997816	2997816	7.5825E-01	2.90E-11	2.44E-09	RHNO1
chr14	75598989	75598989	6.9765E-01	2.93E-11	2.45E-09	TMED10
chr1	3806154	3806154	7.4985E-01	4.76E-11	3.96E-09	C1orf174
chr14	93649246	93649246	6.2384E-01	5.15E-11	4.27E-09	MOAP1
chr16	8947844	8947844	1.7580E+00	5.23E-11	4.31E-09	CARHSP1
chr14	75598994	75598994	1.0310E+00	5.50E-11	4.52E-09	TMED10
chr4	6718861	6718861	1.0318E+00	6.74E-11	5.51E-09	BLOC1S4
chr14	75202667	75202667	-3.9497E-01	7.26E-11	5.91E-09	FCF1
chr4	6718828	6718828	1.3762E+00	7.81E-11	6.34E-09	BLOC1S4
chr3	10143207	10143207	1.4568E+00	8.22E-11	6.64E-09	FANCD2
chr1	21070456	21070456	6.7180E-01	8.30E-11	6.68E-09	HP1BP3

chr13	38936678	38936678	5.7826E-01	8.35E-11	6.69E-09	UFM1
chr12	2997834	2997834	5.3953E-01	9.20E-11	7.28E-09	RHNO1
chr11	6505435	6505435	5.7068E-01	9.19E-11	7.28E-09	TIMM10B
chr1	3806156	3806156	5.4855E-01	9.14E-11	7.28E-09	C1orf174
chr8	104412027	104412027	2.1645E+00	1.04E-10	8.18E-09	SLC25A32
chr14	92630219	92630219	1.1074E+00	1.12E-10	8.78E-09	CPSF2
chr7	75045880	75045880	9.9658E-02	1.24E-10	9.70E-09	NSUN5P1
chr3	57306786	57306786	1.0689E+00	1.34E-10	1.05E-08	APPL1
chr19	34892837	34892837	3.1375E-01	1.53E-10	1.19E-08	GPI
chr11	8941338	8941338	9.8506E-01	1.80E-10	1.39E-08	AKIP1
chr4	6718850	6718850	6.8726E-01	1.95E-10	1.49E-08	BLOC1S4
chr11	8941344	8941344	7.4062E-01	1.95E-10	1.49E-08	AKIP1
chr11	8941226	8941226	7.7594E-01	2.16E-10	1.65E-08	AKIP1
chr1	3697511	3697511	1.0577E+00	2.44E-10	1.85E-08	LRRC47
chr14	75598995	75598995	1.0140E+00	2.92E-10	2.21E-08	TMED10
chr8	22878068	22878068	1.8171E+00	3.36E-10	2.54E-08	TNFRSF10B
chr11	8941244	8941244	7.2282E-01	3.75E-10	2.82E-08	AKIP1
chr17	43002709	43002709	4.4750E-01	3.95E-10	2.95E-08	KIF18B
chr19	1038695	1038695	1.2840E+00	4.07E-10	3.04E-08	CNN2
chr16	8947806	8947806	1.5394E+00	4.52E-10	3.35E-08	CARHSP1
chr12	2997819	2997819	1.0807E+00	4.56E-10	3.37E-08	RHNO1
chr17	29110538	29110538	8.0326E-01	4.77E-10	3.52E-08	CRLF3
chr19	34892846	34892846	3.4805E-01	5.34E-10	3.92E-08	GPI
chr10	105885647	105885647	1.0588E+00	5.42E-10	3.96E-08	SFR1
chr3	57306759	57306759	6.5033E-01	6.78E-10	4.94E-08	APPL1
chr1	21070448	21070448	5.3416E-01	7.63E-10	5.54E-08	HP1BP3
chr19	1038689	1038689	1.1355E+00	7.91E-10	5.72E-08	CNN2
chr17	38458723	38458723	4.4781E-01	7.94E-10	5.72E-08	CDC6
chr13	38936662	38936662	6.8387E-01	1.01E-09	7.26E-08	UFM1
chr15	32930560	32930560	9.7010E-01	1.16E-09	8.29E-08	ARHGAP11A
chr19	59093895	59093895	5.9434E-01	1.17E-09	8.31E-08	CENPBD1P1
chr17	29110539	29110539	6.8611E-01	1.35E-09	9.59E-08	CRLF3
chr17	38458761	38458761	3.3780E-01	1.37E-09	9.66E-08	CDC6
chr9	116169897	116169897	1.8370E+00	1.59E-09	1.12E-07	POLE3
chr8	22878072	22878072	1.4319E+00	1.59E-09	1.12E-07	TNFRSF10B
chr19	59093877	59093877	5.8031E-01	1.61E-09	1.12E-07	CENPBD1P1
chr15	32930567	32930567	7.3684E-01	1.73E-09	1.21E-07	ARHGAP11A
chr17	43002712	43002712	4.4378E-01	1.77E-09	1.23E-07	KIF18B
chr5	174955078	174955078	8.8526E-01	1.85E-09	1.28E-07	SFXN1
chr3	57306788	57306788	9.4439E-01	1.86E-09	1.28E-07	APPL1
chr19	59093891	59093891	5.8713E-01	1.94E-09	1.34E-07	CENPBD1P1

chr19	17453197	17453197	6.5845E-01	1.98E-09	1.36E-07	GTPBP3
chr19	17453221	17453221	5.6697E-01	2.13E-09	1.45E-07	GTPBP3
chr19	34892411	34892411	1.3110E+00	2.18E-09	1.48E-07	GPI
chr14	92630215	92630215	9.7801E-01	2.24E-09	1.51E-07	CPSF2
chr12	133298883	133298883	2.5953E-01	2.23E-09	1.51E-07	PGAM5
chr5	174955064	174955064	9.1512E-01	2.34E-09	1.57E-07	SFXN1
chr8	104412028	104412028	1.4290E+00	2.57E-09	1.72E-07	SLC25A32
chr13	38936660	38936660	7.9888E-01	2.75E-09	1.83E-07	UFM1
chr1	249144372	249144372	1.3141E-01	2.75E-09	1.83E-07	ZNF692
chr8	28048204	28048204	1.1533E+00	2.78E-09	1.84E-07	ELP3
chr17	43002688	43002688	3.9666E-01	2.78E-09	1.84E-07	KIF18B
chr8	104412030	104412030	1.4140E+00	2.86E-09	1.89E-07	SLC25A32
chr19	17453196	17453196	6.5291E-01	2.97E-09	1.95E-07	GTPBP3
chr11	8941342	8941342	7.6504E-01	3.39E-09	2.22E-07	AKIP1
chr7	56078949	56078949	1.0340E+00	3.41E-09	2.22E-07	PSPH
chr11	8941297	8941297	5.6155E-01	3.65E-09	2.38E-07	AKIP1
chr17	38458763	38458763	3.3737E-01	3.91E-09	2.54E-07	CDC6
chr4	166263624	166263624	5.2839E-01	4.30E-09	2.77E-07	MSMO1
chr15	32930561	32930561	7.2679E-01	4.29E-09	2.77E-07	ARHGAP11A
chr11	8941225	8941225	8.1350E-01	4.55E-09	2.92E-07	AKIP1
chr4	6718833	6718833	4.8108E-01	4.82E-09	3.08E-07	BLOC1S4
chr3	20211767	20211767	4.2527E-01	5.34E-09	3.41E-07	SGO1
chrX	24095255	24095255	3.0513E-01	6.12E-09	3.89E-07	EIF2S3
chr12	2997792	2997792	8.2583E-01	6.82E-09	4.32E-07	RHNO1
chr3	33184291	33184291	7.3522E-01	7.02E-09	4.43E-07	CRTAP
chr17	43002713	43002713	4.4924E-01	7.17E-09	4.51E-07	KIF18B
chr11	8941343	8941343	6.8227E-01	7.43E-09	4.66E-07	AKIP1
chr19	34892384	34892384	1.5160E+00	7.71E-09	4.82E-07	GPI
chr19	1038693	1038693	1.1024E+00	7.78E-09	4.85E-07	CNN2
chr19	1038696	1038696	1.2392E+00	7.92E-09	4.92E-07	CNN2
chr15	32930557	32930557	6.2825E-01	8.50E-09	5.26E-07	ARHGAP11A
chr5	174955080	174955080	7.1462E-01	8.74E-09	5.39E-07	SFXN1
chr10	124190697	124190697	1.7906E+00	8.80E-09	5.41E-07	PLEKHA1
chr19	34892834	34892834	2.8501E-01	9.13E-09	5.60E-07	GPI
chr17	38458758	38458758	3.5460E-01	9.23E-09	5.62E-07	CDC6
chr16	8947846	8947846	1.7174E+00	9.22E-09	5.62E-07	CARHSP1
chr3	57306784	57306784	8.3619E-01	9.69E-09	5.89E-07	APPL1
chrX	46741041	46741041	8.4968E-01	9.84E-09	5.96E-07	RP2
chr11	8941224	8941224	1.0194E+00	1.07E-08	6.47E-07	AKIP1
chr10	105885643	105885643	8.1299E-01	1.11E-08	6.69E-07	SFR1
chr5	174955077	174955077	6.0958E-01	1.13E-08	6.77E-07	SFXN1

chr19	17453199	17453199	7.0479E-01	1.15E-08	6.88E-07	GTPBP3
chr17	29110529	29110529	5.8933E-01	1.17E-08	6.96E-07	CRLF3
chr4	6718860	6718860	7.8590E-01	1.22E-08	7.27E-07	BLOC1S4
chr8	28048201	28048201	1.1233E+00	1.26E-08	7.49E-07	ELP3
chr19	34892839	34892839	3.0247E-01	1.29E-08	7.61E-07	GPI
chr9	33046793	33046793	9.4114E-01	1.33E-08	7.84E-07	SMU1
chr19	34892838	34892838	2.8689E-01	1.38E-08	8.11E-07	GPI
chr20	49577397	49577397	6.0217E-01	1.44E-08	8.40E-07	MOCS3
chr3	57306787	57306787	8.2497E-01	1.46E-08	8.52E-07	APPL1
chr8	28048202	28048202	9.5293E-01	1.60E-08	9.32E-07	ELP3
chr1	3806162	3806162	6.1347E-01	1.66E-08	9.65E-07	C1orf174
chr17	29110530	29110530	6.3489E-01	1.73E-08	1.00E-06	CRLF3
chr8	28048213	28048213	6.5378E-01	1.94E-08	1.12E-06	ELP3
chr13	38936658	38936658	6.0899E-01	2.13E-08	1.22E-06	UFM1
chr1	3806157	3806157	3.7159E-01	2.12E-08	1.22E-06	C1orf174
chr10	105885644	105885644	1.0161E+00	2.22E-08	1.27E-06	SFR1
chr19	59093897	59093897	5.8435E-01	2.28E-08	1.30E-06	CENPBD1P1
chr19	1038692	1038692	9.4396E-01	2.38E-08	1.35E-06	CNN2
chr19	59093874	59093874	4.9988E-01	2.52E-08	1.43E-06	CENPBD1P1
chr4	4237888	4237888	1.8408E+00	2.61E-08	1.47E-06	TMEM128
chr8	104412019	104412019	8.5220E-01	2.71E-08	1.52E-06	SLC25A32
chr10	131978463	131978463	Inf	2.85E-08	1.60E-06	GLRX3
chr15	32930558	32930558	6.1350E-01	3.07E-08	1.72E-06	ARHGAP11A
chr11	8941337	8941337	7.7675E-01	3.26E-08	1.82E-06	AKIP1
chr7	56078952	56078952	6.2421E-01	3.28E-08	1.83E-06	PSPH
chr4	6718844	6718844	5.4839E-01	3.71E-08	2.06E-06	BLOC1S4
chr19	17453214	17453214	4.2998E-01	3.91E-08	2.16E-06	GTPBP3
chr14	75203062	75203062	-3.2380E-01	3.93E-08	2.17E-06	FCF1
chr19	1038686	1038686	9.1719E-01	3.97E-08	2.18E-06	CNN2
chr15	32930527	32930527	5.2225E-01	4.52E-08	2.48E-06	ARHGAP11A
chr4	4237886	4237886	1.3151E+00	4.65E-08	2.54E-06	TMEM128
chr3	33184307	33184307	1.4555E+00	4.91E-08	2.68E-06	CRTAP
chr10	124190683	124190683	1.6533E+00	5.03E-08	2.74E-06	PLEKHA1
chr17	43002701	43002701	5.0985E-01	5.61E-08	3.04E-06	KIF18B
chr14	93649232	93649232	6.9994E-01	5.65E-08	3.06E-06	MOAP1
chr10	105885655	105885655	6.6247E-01	6.14E-08	3.31E-06	SFR1
chr15	32930526	32930526	6.5718E-01	6.40E-08	3.44E-06	ARHGAP11A
chr16	50070170	50070170	1.3976E+00	6.72E-08	3.60E-06	CNEP1R1
chr1	3806163	3806163	6.0703E-01	6.94E-08	3.71E-06	C1orf174
chr8	104411996	104411996	2.1305E+00	7.40E-08	3.95E-06	SLC25A32
chr13	38936675	38936675	3.6604E-01	8.77E-08	4.67E-06	UFM1

chrX	24095237	24095237	2.5079E-01	9.13E-08	4.84E-06	EIF2S3
chr8	28048205	28048205	9.5983E-01	9.31E-08	4.93E-06	ELP3
chr19	59093896	59093896	5.1125E-01	9.49E-08	5.01E-06	CENPBD1P1
chr19	17453191	17453191	8.0187E-01	9.82E-08	5.17E-06	GTPBP3
chr15	32930529	32930529	6.4597E-01	1.03E-07	5.43E-06	ARHGAP11A
chr19	17453193	17453193	6.2640E-01	1.10E-07	5.76E-06	GTPBP3
chr7	102277739	102277739	2.2369E-01	1.12E-07	5.84E-06	POLR2J2
chr19	34892415	34892415	9.1377E-01	1.17E-07	6.11E-06	GPI
chr3	33184303	33184303	8.4926E-01	1.25E-07	6.49E-06	CRTAP
chr16	8947807	8947807	7.7034E-01	1.25E-07	6.49E-06	CARHSP1
chr13	38936657	38936657	6.4317E-01	1.28E-07	6.63E-06	UFM1
chr9	116169900	116169900	1.9293E+00	1.30E-07	6.68E-06	POLE3
chr12	6875862	6875862	7.7658E-02	1.31E-07	6.74E-06	PTMS
chr11	46699398	46699398	4.3688E-01	1.35E-07	6.93E-06	ARHGAP1
chr19	1038687	1038687	1.0896E+00	1.38E-07	7.03E-06	CNN2
chr10	124190691	124190691	7.8217E-01	1.54E-07	7.86E-06	PLEKHA1
chr19	34892385	34892385	1.2044E+00	1.71E-07	8.67E-06	GPI
chr19	34892412	34892412	8.9667E-01	1.73E-07	8.78E-06	GPI
chr1	3806160	3806160	4.5639E-01	1.77E-07	8.94E-06	C1orf174
chr14	75203071	75203071	-1.2950E-01	1.94E-07	9.76E-06	FCF1
chr15	32930563	32930563	7.0946E-01	1.95E-07	9.82E-06	ARHGAP11A
chr1	3697426	3697426	8.9802E-01	2.02E-07	1.01E-05	LRRC47
chr20	49577431	49577431	8.2332E-01	2.05E-07	1.02E-05	MOCS3
chr1	21070459	21070459	8.8088E-01	2.06E-07	1.03E-05	HP1BP3
chr7	56079115	56079115	4.0349E-01	2.19E-07	1.09E-05	PSPH
chr3	33184298	33184298	8.7266E-01	2.29E-07	1.14E-05	CRTAP
chr17	38458721	38458721	4.5764E-01	2.38E-07	1.18E-05	CDC6
chr1	9788152	9788152	6.2177E-01	2.41E-07	1.19E-05	PIK3CD
chr3	33184306	33184306	9.9265E-01	2.46E-07	1.21E-05	CRTAP
chr11	27516909	27516909	1.2097E+00	2.60E-07	1.28E-05	LIN7C
chr9	123522786	123522786	Inf	2.67E-07	1.31E-05	FBXW2
chr7	102277713	102277713	1.1834E+00	2.87E-07	1.40E-05	POLR2J2
chr13	38936682	38936682	3.5298E-01	2.89E-07	1.41E-05	UFM1
chr16	1495765	1495765	4.7642E-01	2.91E-07	1.41E-05	CLCN7
chr15	32930566	32930566	5.5676E-01	3.06E-07	1.49E-05	ARHGAP11A
chr10	131978469	131978469	1.0662E+00	3.12E-07	1.51E-05	GLRX3
chr6	18130383	18130383	6.2176E-01	3.14E-07	1.52E-05	TPMT
chr8	28048196	28048196	1.1001E+00	3.21E-07	1.55E-05	ELP3
chr9	116169898	116169898	2.0394E+00	3.39E-07	1.63E-05	POLE3
chr8	22878069	22878069	1.2922E+00	3.51E-07	1.68E-05	TNFRSF10B
chr7	138745319	138745319	1.5918E+00	3.59E-07	1.71E-05	ZC3HAV1

chr3	33184287	33184287	9.5773E-01	3.65E-07	1.74E-05	CRTAP
chr17	29110521	29110521	4.3550E-01	3.65E-07	1.74E-05	CRLF3
chr4	4237875	4237875	1.6437E+00	3.79E-07	1.80E-05	TMEM128
chr14	92630235	92630235	1.3544E+00	3.80E-07	1.80E-05	CPSF2
chr9	116169901	116169901	1.5876E+00	3.83E-07	1.81E-05	POLE3
chr4	4237889	4237889	1.0430E+00	3.98E-07	1.88E-05	TMEM128
chr16	29754168	29754168	1.2341E+00	4.07E-07	1.91E-05	C16orf54
chr19	34892421	34892421	1.0261E+00	4.09E-07	1.92E-05	GPI
chr19	59093890	59093890	4.2664E-01	4.21E-07	1.97E-05	CENPBD1P1
chr7	56079117	56079117	3.9826E-01	4.22E-07	1.97E-05	PSPH
chr19	59093888	59093888	4.9292E-01	4.44E-07	2.07E-05	CENPBD1P1
chr4	73922609	73922609	5.3557E-01	4.53E-07	2.10E-05	COX18
chr4	166263623	166263623	5.6992E-01	4.77E-07	2.21E-05	MSMO1
chr3	20211770	20211770	3.2805E-01	5.10E-07	2.36E-05	SGO1
chr11	6505433	6505433	7.4611E-01	5.40E-07	2.49E-05	TIMM10B
chr10	131978472	131978472	1.3114E+00	5.48E-07	2.52E-05	GLRX3
chr10	89619514	89619514	9.3459E-01	5.77E-07	2.65E-05	KLLN
chr15	32930531	32930531	4.1124E-01	5.90E-07	2.70E-05	ARHGAP11A
chr19	34892372	34892372	2.6576E+00	5.92E-07	2.70E-05	GPI
chr7	56079108	56079108	3.3401E-01	5.95E-07	2.71E-05	PSPH
chr2	197000375	197000375	2.5264E+00	5.96E-07	2.71E-05	STK17B
chr17	29110546	29110546	2.2047E+00	6.04E-07	2.74E-05	CRLF3
chr19	1038683	1038683	1.2102E+00	6.60E-07	2.98E-05	CNN2
chr10	105885646	105885646	8.2485E-01	6.59E-07	2.98E-05	SFR1
chr15	32930565	32930565	4.4589E-01	6.63E-07	2.98E-05	ARHGAP11A
chr19	34892417	34892417	8.9741E-01	6.82E-07	3.06E-05	GPI
chr3	57306752	57306752	4.2039E-01	6.93E-07	3.11E-05	APPL1
chr16	21191713	21191713	4.9310E-01	7.34E-07	3.28E-05	TMEM159
chr7	138745318	138745318	1.5901E+00	7.60E-07	3.39E-05	ZC3HAV1
chr13	38936692	38936692	5.8393E-01	7.74E-07	3.44E-05	UFM1
chr19	34892381	34892381	8.8393E-01	7.77E-07	3.45E-05	GPI
chr3	57306750	57306750	5.1382E-01	7.80E-07	3.45E-05	APPL1
chr6	18130382	18130382	5.3112E-01	8.05E-07	3.55E-05	TPMT
chr7	138745316	138745316	2.0363E+00	8.17E-07	3.60E-05	ZC3HAV1
chr3	20211783	20211783	3.8237E-01	8.48E-07	3.73E-05	SGO1
chr22	39128852	39128852	2.2582E+00	8.90E-07	3.90E-05	GTPBP1
chr22	39128838	39128838	1.6126E+00	9.24E-07	4.04E-05	GTPBP1
chrX	46741032	46741032	5.5682E-01	9.35E-07	4.08E-05	RP2
chr19	34892405	34892405	8.6852E-01	1.03E-06	4.49E-05	GPI
chr7	138745360	138745360	Inf	1.13E-06	4.93E-05	ZC3HAV1
chr14	92630234	92630234	1.1981E+00	1.15E-06	4.98E-05	CPSF2

chr16	29754182	29754182	1.9375E+00	1.16E-06	5.00E-05	C16orf54
chr8	104412013	104412013	8.3388E-01	1.19E-06	5.15E-05	SLC25A32
chr19	34892835	34892835	2.8753E-01	1.21E-06	5.23E-05	GPI
chr10	131978476	131978476	1.3031E+00	1.28E-06	5.52E-05	GLRX3
chr4	6718862	6718862	1.4342E+00	1.34E-06	5.73E-05	BLOC1S4
chr10	131978475	131978475	1.0515E+00	1.36E-06	5.80E-05	GLRX3
chr7	56079120	56079120	3.6217E-01	1.37E-06	5.83E-05	PSPH
chr11	65180579	65180579	5.0045E-01	1.39E-06	5.91E-05	FRMD8
chr15	41573181	41573181	6.8632E-01	1.40E-06	5.96E-05	CHP1
chr5	10435945	10435945	Inf	1.42E-06	6.02E-05	MARCH6
chr16	3022796	3022796	1.7886E-01	1.46E-06	6.16E-05	PAQR4
chr1	21070457	21070457	8.1015E-01	1.55E-06	6.53E-05	HP1BP3
chr19	34892377	34892377	8.7030E-01	1.56E-06	6.55E-05	GPI
chr19	36032254	36032254	1.1273E+00	1.56E-06	6.55E-05	TMEM147-AS1
chr16	21191708	21191708	3.9000E-01	1.57E-06	6.56E-05	TMEM159
chr16	29754166	29754166	1.2623E+00	1.60E-06	6.71E-05	C16orf54
chr10	131978462	131978462	1.6727E+00	1.63E-06	6.80E-05	GLRX3
chr10	89619516	89619516	7.4539E-01	1.63E-06	6.80E-05	KLLN
chr11	27516908	27516908	9.6070E-01	1.81E-06	7.52E-05	LIN7C
chr17	29110543	29110543	1.0010E+00	1.82E-06	7.54E-05	CRLF3
chr2	202151470	202151470	4.8846E-01	1.83E-06	7.55E-05	CASP8
chr16	21191717	21191717	1.7058E+00	1.87E-06	7.71E-05	TMEM159
chr8	28048195	28048195	6.9374E-01	1.89E-06	7.74E-05	ELP3
chr7	56079099	56079099	4.4291E-01	1.89E-06	7.75E-05	PSPH
chr11	46699395	46699395	6.0288E-01	1.95E-06	7.98E-05	ARHGAP1
chr12	56215547	56215547	1.7624E+00	1.96E-06	8.02E-05	DNAJC14
chr7	102277715	102277715	7.5841E-01	2.00E-06	8.15E-05	POLR2J2
chr11	46699397	46699397	5.4600E-01	2.10E-06	8.53E-05	ARHGAP1
chr19	59093887	59093887	4.3900E-01	2.30E-06	9.34E-05	CENPBD1P1
chr16	21191704	21191704	3.7032E-01	2.34E-06	9.47E-05	TMEM159
chr20	49577395	49577395	5.1213E-01	2.35E-06	9.51E-05	MOCS3
chr11	66034954	66034954	2.1855E-01	2.40E-06	9.69E-05	KLC2
chr20	49577435	49577435	7.1979E-01	2.41E-06	9.71E-05	MOCS3
chr11	27516901	27516901	1.2085E+00	2.47E-06	9.93E-05	LIN7C
chr22	24937475	24937475	4.3032E-01	2.49E-06	9.99E-05	GUCD1
chr10	105885648	105885648	4.8075E-01	2.51E-06	1.00E-04	SFR1
chrX	46407030	46407030	5.8332E-01	2.52E-06	1.00E-04	ZNF674-AS1
chr14	93649250	93649250	5.5544E-01	2.58E-06	1.03E-04	MOAP1
chr12	56215535	56215535	1.1232E+00	2.59E-06	1.03E-04	DNAJC14
chr16	29754172	29754172	1.2962E+00	2.73E-06	1.08E-04	C16orf54
chr8	22878066	22878066	2.0724E+00	2.77E-06	1.10E-04	TNFRSF10B

chr3	57306751	57306751	4.1366E-01	2.83E-06	1.11E-04	APPL1
chr17	59943595	59943595	1.2826E+00	2.82E-06	1.11E-04	INTS2
chr15	41573176	41573176	6.0711E-01	2.83E-06	1.11E-04	CHP1
chr11	46699394	46699394	4.8800E-01	2.88E-06	1.13E-04	ARHGAP1
chr9	135251165	135251165	-5.5141E-01	2.94E-06	1.15E-04	TTF1
chr16	50070173	50070173	1.0139E+00	2.95E-06	1.15E-04	CNEP1R1
chr15	41573177	41573177	6.8409E-01	3.02E-06	1.18E-04	CHP1
chr22	24937490	24937490	1.2476E+00	3.13E-06	1.22E-04	GUCD1
chr20	49577394	49577394	5.6995E-01	3.29E-06	1.28E-04	MOCS3
chr22	39128824	39128824	2.5002E+00	3.42E-06	1.33E-04	GTPBP1
chr15	41573170	41573170	7.4883E-01	3.47E-06	1.34E-04	CHP1
chr3	33184310	33184310	1.0804E+00	3.53E-06	1.36E-04	CRTAP
chr12	72179802	72179802	4.0826E-01	3.54E-06	1.36E-04	RAB21
chr11	8941324	8941324	7.0495E-01	3.56E-06	1.37E-04	AKIP1
chr7	56078951	56078951	2.6962E-01	3.77E-06	1.45E-04	PSPH
chrX	46741030	46741030	4.6038E-01	3.86E-06	1.48E-04	RP2
chr19	59093893	59093893	5.2844E-01	3.87E-06	1.48E-04	CENPBD1P1
chr8	104412000	104412000	1.0752E+00	3.94E-06	1.50E-04	SLC25A32
chr12	57648150	57648150	2.1692E+00	3.94E-06	1.50E-04	R3HDM2
chr3	156258127	156258127	7.3603E-01	4.01E-06	1.52E-04	SSR3
chr12	56215548	56215548	1.7551E+00	4.07E-06	1.54E-04	DNAJC14
chr16	29754179	29754179	1.2751E+00	4.16E-06	1.57E-04	C16orf54
chr19	59093856	59093856	5.4939E-01	4.41E-06	1.67E-04	CENPBD1P1
chrX	46407051	46407051	1.1597E+00	4.45E-06	1.68E-04	ZNF674-AS1
chr11	65542157	65542157	2.1863E+00	4.45E-06	1.68E-04	AP5B1
chr3	57306748	57306748	4.1519E-01	4.49E-06	1.69E-04	APPL1
chr11	27516912	27516912	9.6032E-01	4.54E-06	1.70E-04	LIN7C
chrX	24095339	24095339	6.9796E-01	4.57E-06	1.71E-04	EIF2S3
chr20	49577434	49577434	7.0930E-01	4.56E-06	1.71E-04	MOCS3
chr11	27516916	27516916	9.6029E-01	4.58E-06	1.71E-04	LIN7C
chr19	59093857	59093857	5.4257E-01	4.67E-06	1.74E-04	CENPBD1P1
chr10	105885683	105885683	6.2668E-01	4.67E-06	1.74E-04	SFR1
chr1	28662098	28662098	1.3820E-01	4.95E-06	1.83E-04	MED18
chr16	29754174	29754174	1.2717E+00	5.16E-06	1.91E-04	C16orf54
chr20	35280696	35280696	4.8020E-01	5.25E-06	1.94E-04	NDRG3
chr16	29754165	29754165	1.2677E+00	5.38E-06	1.98E-04	C16orf54
chr11	490937	490937	5.5601E-01	5.51E-06	2.03E-04	PTDSS2
chr19	17453044	17453044	4.2392E-01	5.63E-06	2.07E-04	GTPBP3
chr12	109887425	109887425	1.0550E+00	5.64E-06	2.07E-04	KCTD10
chr1	204392462	204392462	5.0746E-01	5.66E-06	2.07E-04	PIK3C2B
chr14	92630229	92630229	7.6676E-01	5.68E-06	2.07E-04	CPSF2

chr13	38936686	38936686	4.9884E-01	5.71E-06	2.08E-04	UFM1
chr19	36032253	36032253	1.1141E+00	5.82E-06	2.12E-04	TMEM147-AS1
chr7	56079179	56079179	4.2516E-01	5.88E-06	2.13E-04	PSPH
chr14	75202665	75202665	-1.3003E-01	5.90E-06	2.14E-04	FCF1
chr7	72419023	72419023	1.7887E-01	5.98E-06	2.16E-04	NSUN5P2
chr19	34892380	34892380	7.5136E-01	6.11E-06	2.20E-04	GPI
chr19	1038682	1038682	1.0910E+00	6.12E-06	2.21E-04	CNN2
chr10	131978470	131978470	1.2748E+00	6.20E-06	2.23E-04	GLRX3
chr4	83551853	83551853	1.0415E+00	6.40E-06	2.29E-04	SCD5
chr11	6505432	6505432	7.7997E-01	6.39E-06	2.29E-04	TIMM10B
chr20	35280693	35280693	4.1754E-01	6.55E-06	2.34E-04	NDRG3
chr1	9788154	9788154	5.9333E-01	6.66E-06	2.38E-04	PIK3CD
chr12	56215538	56215538	8.0282E-01	6.88E-06	2.45E-04	DNAJC14
chr10	105885638	105885638	9.5628E-01	6.91E-06	2.46E-04	SFR1
chr22	24937492	24937492	5.7854E-01	7.05E-06	2.50E-04	GUCD1
chr16	21191705	21191705	4.0102E-01	7.32E-06	2.59E-04	TMEM159
chr13	38936694	38936694	5.6825E-01	7.46E-06	2.64E-04	UFM1
chr8	77894942	77894942	2.4199E+00	7.89E-06	2.78E-04	PEX2
chr2	197000365	197000365	1.8563E+00	7.89E-06	2.78E-04	STK17B
chr12	72179806	72179806	4.0376E-01	7.88E-06	2.78E-04	RAB21
chr20	35280702	35280702	3.5594E-01	8.22E-06	2.88E-04	NDRG3
chr14	92630206	92630206	5.0435E-01	8.22E-06	2.88E-04	CPSF2
chr19	36032255	36032255	1.4836E+00	8.55E-06	2.99E-04	TMEM147-AS1
chr12	109887417	109887417	8.4644E-01	8.67E-06	3.02E-04	KCTD10
chr8	22878071	22878071	1.5056E+00	8.76E-06	3.05E-04	TNFRSF10B
chr1	100757990	100757990	2.7317E-01	8.90E-06	3.09E-04	RTCA
chr15	41573179	41573179	7.4722E-01	8.97E-06	3.11E-04	CHP1
chr20	39802812	39802812	3.6757E-01	1.00E-05	3.47E-04	PLCG1
chr19	34892414	34892414	7.2085E-01	1.01E-05	3.48E-04	GPI
chr1	115312912	115312912	8.4199E-01	1.01E-05	3.48E-04	SIKE1
chr16	2508384	2508384	2.6961E-01	1.01E-05	3.48E-04	CCNF
chr7	102277741	102277741	2.1971E-01	1.02E-05	3.51E-04	POLR2J2
chr6	18130379	18130379	5.4141E-01	1.04E-05	3.57E-04	TPMT
chr15	41573180	41573180	6.1024E-01	1.05E-05	3.59E-04	CHP1
chr1	23833903	23833903	2.3014E+00	1.06E-05	3.63E-04	E2F2
chr1	204392469	204392469	7.1351E-01	1.08E-05	3.68E-04	PIK3C2B
chr8	77894939	77894939	2.3612E+00	1.10E-05	3.72E-04	PEX2
chr17	43002716	43002716	4.2502E-01	1.10E-05	3.72E-04	KIF18B
chr16	21191714	21191714	3.5901E-01	1.10E-05	3.72E-04	TMEM159
chr11	27516910	27516910	1.1778E+00	1.10E-05	3.72E-04	LIN7C
chr1	3806136	3806136	1.5331E+00	1.10E-05	3.73E-04	C1orf174

chr14	92630231	92630231	6.4845E-01	1.11E-05	3.75E-04	CPSF2
chr15	32930530	32930530	4.3157E-01	1.11E-05	3.75E-04	ARHGAP11A
chrX	46741033	46741033	4.5246E-01	1.15E-05	3.87E-04	RP2
chr10	131978466	131978466	1.6450E+00	1.15E-05	3.87E-04	GLRX3
chr12	107391384	107391384	1.1858E-01	1.16E-05	3.89E-04	CRY1
chr8	104412006	104412006	6.9360E-01	1.19E-05	4.00E-04	SLC25A32
chr3	20211784	20211784	3.3735E-01	1.20E-05	4.02E-04	SGO1
chr22	24937508	24937508	1.0188E+00	1.26E-05	4.19E-04	GUCD1
chr4	1193082	1193082	1.8292E+00	1.27E-05	4.22E-04	LOC100130872
chr19	59093881	59093881	4.4445E-01	1.29E-05	4.27E-04	CENPBD1P1
chr13	38936688	38936688	4.1575E-01	1.30E-05	4.32E-04	UFM1
chr3	156258119	156258119	6.7023E-01	1.34E-05	4.43E-04	SSR3
chr15	41573173	41573173	7.3386E-01	1.38E-05	4.55E-04	CHP1
chr1	179832247	179832247	4.1092E-01	1.39E-05	4.60E-04	TOR1AIP2
chr17	38458720	38458720	5.3164E-01	1.40E-05	4.60E-04	CDC6
chr4	1813580	1813580	7.7920E-01	1.41E-05	4.63E-04	LETM1
chr17	29110545	29110545	9.5791E-01	1.44E-05	4.72E-04	CRLF3
chr10	855695	855695	4.2075E-01	1.47E-05	4.82E-04	LARP4B
chr10	105885684	105885684	9.2306E-01	1.59E-05	5.20E-04	SFR1
chr8	77894938	77894938	2.3500E+00	1.61E-05	5.25E-04	PEX2
chr9	116169885	116169885	2.2112E+00	1.61E-05	5.26E-04	POLE3
chrX	46407033	46407033	5.8298E-01	1.66E-05	5.38E-04	ZNF674-AS1
chr4	4237890	4237890	9.9357E-01	1.66E-05	5.38E-04	TMEM128
chr20	49577432	49577432	5.5309E-01	1.65E-05	5.38E-04	MOCS3
chr8	104411999	104411999	7.1400E-01	1.67E-05	5.43E-04	SLC25A32
chr2	61412650	61412650	4.9689E-01	1.89E-05	6.13E-04	AHSA2
chr12	72179813	72179813	4.0694E-01	1.95E-05	6.28E-04	RAB21
chr12	72179805	72179805	3.5709E-01	1.96E-05	6.30E-04	RAB21
chrX	46741029	46741029	3.3162E-01	1.99E-05	6.39E-04	RP2
chr4	83551916	83551916	2.2405E+00	2.05E-05	6.55E-04	SCD5
chr22	24937491	24937491	7.7287E-01	2.04E-05	6.55E-04	GUCD1
chr17	59943587	59943587	8.9185E-01	2.12E-05	6.78E-04	INTS2
chr20	49577429	49577429	8.7745E-01	2.13E-05	6.79E-04	MOCS3
chr7	56079102	56079102	3.3346E-01	2.17E-05	6.91E-04	PSPH
chr1	100757979	100757979	3.1845E-01	2.19E-05	6.97E-04	RTCA
chr1	100757982	100757982	3.1751E-01	2.19E-05	6.97E-04	RTCA
chr17	43002541	43002541	6.3479E-01	2.21E-05	7.02E-04	KIF18B
chr16	75600303	75600303	9.7821E-01	2.23E-05	7.07E-04	GABARAPL2
chr7	2703820	2703820	-6.0809E-02	2.24E-05	7.08E-04	TTYH3
chr8	22878082	22878082	1.2709E+00	2.27E-05	7.17E-04	TNFRSF10B
chr3	57306791	57306791	1.0614E+00	2.30E-05	7.23E-04	APPL1

chr9	116169916	116169916	2.5393E+00	2.33E-05	7.34E-04	POLE3
chr7	138745310	138745310	1.3590E+00	2.36E-05	7.40E-04	ZC3HAV1
chr8	77894946	77894946	2.3658E+00	2.38E-05	7.43E-04	PEX2
chr7	102277738	102277738	2.1441E-01	2.37E-05	7.43E-04	POLR2J2
chr2	120978199	120978199	7.2154E-01	2.39E-05	7.47E-04	TMEM185B
chr17	57188048	57188048	7.9787E-01	2.46E-05	7.67E-04	SKA2
chr17	20904240	20904240	5.5968E-01	2.49E-05	7.76E-04	USP22
chr16	29754175	29754175	9.1555E-01	2.52E-05	7.84E-04	C16orf54
chr12	56215536	56215536	7.9106E-01	2.55E-05	7.92E-04	DNAJC14
chr12	109887426	109887426	1.0506E+00	2.56E-05	7.93E-04	KCTD10
chr9	116169884	116169884	2.6749E+00	2.57E-05	7.94E-04	POLE3
chr16	29754177	29754177	9.0072E-01	2.57E-05	7.94E-04	C16orf54
chr15	32930570	32930570	5.3588E-01	2.58E-05	7.95E-04	ARHGAP11A
chr1	3729126	3729126	3.3846E-01	2.59E-05	7.96E-04	CEP104
chr22	24937462	24937462	5.6212E-01	2.62E-05	8.05E-04	GUCD1
chr12	56215549	56215549	1.7026E+00	2.63E-05	8.08E-04	DNAJC14
chr2	197000397	197000397	1.5757E+00	2.65E-05	8.11E-04	STK17B
chr20	35280710	35280710	2.4773E-01	2.66E-05	8.15E-04	NDRG3
chr6	18130385	18130385	5.2585E-01	2.78E-05	8.51E-04	TPMT
chr4	83551852	83551852	6.4680E-01	2.79E-05	8.51E-04	SCD5
chr16	21191707	21191707	2.8910E-01	2.83E-05	8.63E-04	TMEM159
chr17	43002787	43002787	5.4159E-01	2.93E-05	8.92E-04	KIF18B
chr17	43002545	43002545	6.3329E-01	3.05E-05	9.26E-04	KIF18B
chr11	27516913	27516913	6.5404E-01	3.13E-05	9.49E-04	LIN7C
chr1	26160972	26160972	9.9919E-01	3.15E-05	9.54E-04	AUNIP
chr13	38936691	38936691	3.7581E-01	3.16E-05	9.54E-04	UFM1
chr9	132572112	132572112	1.1527E+00	3.18E-05	9.57E-04	TOR1B
chr7	92166478	92166478	-2.9215E-01	3.17E-05	9.57E-04	RBM48
chr3	57306747	57306747	2.5254E-01	3.18E-05	9.57E-04	APPL1
chr22	39128841	39128841	1.5674E+00	3.20E-05	9.59E-04	GTPBP1
chr10	105885687	105885687	1.3084E+00	3.23E-05	9.68E-04	SFR1
chr7	138745311	138745311	2.0471E+00	3.28E-05	9.80E-04	ZC3HAV1
chr19	11433654	11433654	Inf	3.29E-05	9.80E-04	RAB3D
chr16	21191712	21191712	3.8536E-01	3.29E-05	9.80E-04	TMEM159
chr2	202151393	202151393	5.0270E-01	3.31E-05	9.86E-04	CASP8
chr11	65180583	65180583	6.5284E-01	3.33E-05	9.89E-04	FRMD8
chr22	24937493	24937493	6.9009E-01	3.43E-05	1.02E-03	GUCD1
chr2	202151462	202151462	3.9434E-01	3.50E-05	1.04E-03	CASP8
chr19	36032246	36032246	9.0430E-01	3.50E-05	1.04E-03	TMEM147-AS1
chr1	26161006	26161006	1.6211E+00	3.51E-05	1.04E-03	AUNIP
chr4	4237914	4237914	2.4314E+00	3.57E-05	1.05E-03	TMEM128

chr19	11433649	11433649	Inf	3.61E-05	1.06E-03	RAB3D
chr4	83551856	83551856	8.1797E-01	3.81E-05	1.12E-03	SCD5
chrX	46741026	46741026	3.8067E-01	3.87E-05	1.13E-03	RP2
chr19	17453190	17453190	5.7391E-01	3.94E-05	1.15E-03	GTPBP3
chr19	36032257	36032257	1.1118E+00	3.94E-05	1.15E-03	TMEM147-AS1
chr16	29754178	29754178	1.2651E+00	3.95E-05	1.15E-03	C16orf54
chr10	124190677	124190677	2.3001E+00	3.94E-05	1.15E-03	PLEKHA1
chr8	104411994	104411994	6.7818E-01	4.06E-05	1.18E-03	SLC25A32
chr16	8947912	8947912	9.4355E-01	4.12E-05	1.20E-03	CARHSP1
chr12	56215539	56215539	6.2183E-01	4.19E-05	1.22E-03	DNAJC14
chr5	174955178	174955178	1.4552E+00	4.25E-05	1.23E-03	SFXN1
chr1	23833902	23833902	1.5068E+00	4.27E-05	1.24E-03	E2F2
chr8	28048133	28048133	5.7055E-01	4.30E-05	1.24E-03	ELP3
chr1	23833866	23833866	1.9474E+00	4.31E-05	1.24E-03	E2F2
chr19	34892420	34892420	7.1298E-01	4.33E-05	1.25E-03	GPI
chr3	156258126	156258126	6.4906E-01	4.42E-05	1.27E-03	SSR3
chr9	33045729	33045729	Inf	4.45E-05	1.28E-03	SMU1
chr15	32930521	32930521	4.3764E-01	4.46E-05	1.28E-03	ARHGAP11A
chr12	109887428	109887428	9.9786E-01	4.51E-05	1.29E-03	KCTD10
chr10	124190679	124190679	1.1907E+00	4.53E-05	1.29E-03	PLEKHA1
chr1	100757983	100757983	3.1034E-01	4.69E-05	1.34E-03	RTCA
chrX	46407059	46407059	1.1372E+00	4.81E-05	1.37E-03	ZNF674-AS1
chr17	59943589	59943589	6.7198E-01	4.88E-05	1.38E-03	INTS2
chr12	109887424	109887424	1.0450E+00	4.94E-05	1.40E-03	KCTD10
chr4	83551854	83551854	8.1357E-01	4.97E-05	1.41E-03	SCD5
chr1	23833894	23833894	1.5210E+00	5.18E-05	1.46E-03	E2F2
chr10	89619504	89619504	2.1332E+00	5.19E-05	1.47E-03	KLLN
chr4	83551845	83551845	6.2426E-01	5.20E-05	1.47E-03	SCD5
chr16	87426015	87426015	2.0587E+00	5.34E-05	1.50E-03	MAP1LC3B
chr1	28661805	28661805	8.5046E-02	5.34E-05	1.50E-03	MED18
chr17	43002543	43002543	5.8336E-01	5.39E-05	1.51E-03	KIF18B
chr19	11433651	11433651	Inf	5.55E-05	1.56E-03	RAB3D
chr2	202151463	202151463	3.5078E-01	5.62E-05	1.57E-03	CASP8
chr7	56079193	56079193	2.3507E-01	5.70E-05	1.59E-03	PSPH
chr10	105885678	105885678	6.4969E-01	5.80E-05	1.62E-03	SFR1
chr22	30093675	30093675	6.9868E-01	5.83E-05	1.62E-03	NF2
chr12	72179804	72179804	3.5058E-01	5.92E-05	1.65E-03	RAB21
chr17	5393310	5393310	8.1763E-01	5.94E-05	1.65E-03	MIS12
chr3	155480837	155480837	6.2473E-01	5.96E-05	1.65E-03	C3orf33
chr4	4237915	4237915	1.8049E+00	5.98E-05	1.66E-03	TMEM128
chr4	1813584	1813584	7.7212E-01	6.02E-05	1.67E-03	LETM1

chr16	75600300	75600300	8.9418E-01	6.03E-05	1.67E-03	GABARAPL2
chr19	34892401	34892401	1.2700E+00	6.10E-05	1.68E-03	GPI
chr12	56390381	56390381	1.8403E+00	6.12E-05	1.68E-03	RAB5B
chr1	26161002	26161002	1.6104E+00	6.12E-05	1.68E-03	AUNIP
chr17	43002542	43002542	5.8988E-01	6.29E-05	1.73E-03	KIF18B
chr11	65180576	65180576	5.4775E-01	6.49E-05	1.78E-03	FRMD8
chr10	131978410	131978410	2.0227E+00	6.51E-05	1.78E-03	GLRX3
chr7	56079095	56079095	5.0262E-01	6.56E-05	1.79E-03	PSPH
chr16	50070190	50070190	6.6345E-01	6.60E-05	1.80E-03	CNEP1R1
chr11	46699401	46699401	4.0364E-01	6.73E-05	1.83E-03	ARHGAP1
chr7	138745353	138745353	2.0129E+00	6.81E-05	1.85E-03	ZC3HAV1
chr5	10435980	10435980	2.3627E+00	6.95E-05	1.89E-03	MARCH6
chr17	5393313	5393313	8.1444E-01	7.13E-05	1.93E-03	MIS12
chr6	20102075	20102075	1.9570E+00	7.17E-05	1.94E-03	MBOAT1
chr16	50070187	50070187	5.4564E-01	7.26E-05	1.96E-03	CNEP1R1
chr20	57486142	57486142	1.4886E+00	7.39E-05	1.99E-03	GNAS
chr17	38458757	38458757	3.7597E-01	7.48E-05	2.01E-03	CDC6
chr11	27516888	27516888	1.1657E+00	7.57E-05	2.04E-03	LIN7C
chr4	83551857	83551857	7.8566E-01	7.61E-05	2.04E-03	SCD5
chr1	23833911	23833911	2.1708E+00	7.67E-05	2.06E-03	E2F2
chr15	41573171	41573171	5.2685E-01	7.76E-05	2.08E-03	CHP1
chr10	89619511	89619511	5.4238E-01	7.79E-05	2.08E-03	KLLN
chr22	24937507	24937507	1.1798E+00	7.83E-05	2.09E-03	GUCD1
chr5	10435942	10435942	Inf	8.00E-05	2.13E-03	MARCH6
chr1	204392471	204392471	6.1414E-01	8.21E-05	2.18E-03	PIK3C2B
chr20	35280714	35280714	2.3866E-01	8.23E-05	2.19E-03	NDRG3
chr1	115312913	115312913	8.0154E-01	8.40E-05	2.23E-03	SIKE1
chr4	159630579	159630579	9.3030E-01	8.43E-05	2.23E-03	PPID
chr2	202151459	202151459	3.9992E-01	8.62E-05	2.27E-03	CASP8
chr10	131978487	131978487	1.2215E+00	8.74E-05	2.30E-03	GLRX3
chr3	141664015	141664015	1.3461E-01	8.76E-05	2.30E-03	TFDP2
chr14	92630228	92630228	5.6528E-01	8.78E-05	2.30E-03	CPSF2
chr22	39128821	39128821	1.2829E+00	8.84E-05	2.31E-03	GTPBP1
chr14	92630191	92630191	7.1548E-01	8.85E-05	2.31E-03	CPSF2
chr7	56079119	56079119	2.8532E-01	8.92E-05	2.33E-03	PSPH
chr22	39128855	39128855	1.1813E+00	9.01E-05	2.35E-03	GTPBP1
chr1	26160975	26160975	9.6362E-01	9.02E-05	2.35E-03	AUNIP
chr4	1381024	1381024	2.2286E+00	9.14E-05	2.38E-03	UVSSA
chr8	22878063	22878063	1.8753E+00	9.28E-05	2.41E-03	TNFRSF10B
chr1	3806023	3806023	1.0894E+00	9.27E-05	2.41E-03	C1orf174
chr9	33045720	33045720	2.5039E+00	9.60E-05	2.48E-03	SMU1

chr10	105885682	105885682	5.8661E-01	9.59E-05	2.48E-03	SFR1
chr1	23833905	23833905	2.2558E+00	9.58E-05	2.48E-03	E2F2
chr17	59943598	59943598	1.0527E+00	9.71E-05	2.50E-03	INTS2
chr16	87426006	87426006	Inf	9.74E-05	2.51E-03	MAP1LC3B
chr20	35280713	35280713	2.4051E-01	9.87E-05	2.53E-03	NDRG3
chr16	29754181	29754181	8.5521E-01	1.00E-04	2.57E-03	C16orf54
chr4	664183	664183	-1.5102E+00	1.02E-04	2.60E-03	PDE6B
chr22	24937501	24937501	6.7449E-01	1.02E-04	2.61E-03	GUCD1
chr19	34892831	34892831	2.8645E-01	1.02E-04	2.61E-03	GPI
chr19	37002422	37002422	1.1122E+00	1.03E-04	2.62E-03	ZNF260
chr11	27516892	27516892	5.6141E-01	1.03E-04	2.63E-03	LIN7C
chr10	105885674	105885674	7.1654E-01	1.03E-04	2.63E-03	SFR1
chr11	65180582	65180582	6.0951E-01	1.05E-04	2.65E-03	FRMD8
chr11	27516919	27516919	9.1689E-01	1.05E-04	2.65E-03	LIN7C
chrX	46407035	46407035	4.7467E-01	1.05E-04	2.67E-03	ZNF674-AS1
chr22	36623223	36623223	5.4719E-01	1.08E-04	2.72E-03	APOL2
chr4	1381016	1381016	2.2631E+00	1.09E-04	2.74E-03	UVSSA
chr12	2997740	2997740	7.0103E-01	1.10E-04	2.77E-03	RHNO1
chr22	24937500	24937500	6.0443E-01	1.11E-04	2.78E-03	GUCD1
chr16	2508375	2508375	2.7852E-01	1.13E-04	2.84E-03	CCNF
chr19	48984892	48984892	1.0289E+00	1.15E-04	2.88E-03	CYTH2
chr16	8947916	8947916	1.3633E+00	1.15E-04	2.89E-03	CARHSP1
chr14	92630188	92630188	5.0259E-01	1.16E-04	2.91E-03	CPSF2
chr4	6718827	6718827	7.9212E-01	1.17E-04	2.93E-03	BLOC1S4
chr19	11433652	11433652	Inf	1.17E-04	2.93E-03	RAB3D
chr12	57648181	57648181	6.5482E-01	1.17E-04	2.93E-03	R3HDM2
chr12	57648147	57648147	2.1198E+00	1.19E-04	2.98E-03	R3HDM2
chr8	77894948	77894948	2.3494E+00	1.21E-04	3.00E-03	PEX2
chr19	11433647	11433647	Inf	1.22E-04	3.02E-03	RAB3D
chr8	77894949	77894949	2.2902E+00	1.22E-04	3.04E-03	PEX2
chr7	2703814	2703814	-4.4413E-02	1.24E-04	3.06E-03	TTYH3
chr1	204373511	204373511	1.0265E-01	1.25E-04	3.09E-03	PPP1R15B
chrX	24095236	24095236	2.1628E-01	1.25E-04	3.10E-03	EIF2S3
chr19	48984894	48984894	9.4767E-01	1.31E-04	3.23E-03	CYTH2
chr12	56390382	56390382	1.8271E+00	1.31E-04	3.23E-03	RAB5B
chr19	1038671	1038671	1.4626E+00	1.34E-04	3.31E-03	CNN2
chrX	46407031	46407031	4.6556E-01	1.35E-04	3.32E-03	ZNF674-AS1
chr8	77894940	77894940	1.5750E+00	1.36E-04	3.35E-03	PEX2
chr10	89619517	89619517	5.9563E-01	1.36E-04	3.35E-03	KLLN
chr16	50070169	50070169	9.6794E-01	1.37E-04	3.36E-03	CNEP1R1
chr7	138745356	138745356	Inf	1.37E-04	3.36E-03	ZC3HAV1

chr7	154795375	154795375	3.4809E-01	1.38E-04	3.38E-03	PAXIP1-AS1
chr12	56390376	56390376	1.1386E+00	1.41E-04	3.45E-03	RAB5B
chr16	75478247	75478247	6.4526E-01	1.42E-04	3.47E-03	TMEM170A
chr16	29754164	29754164	1.2218E+00	1.42E-04	3.47E-03	C16orf54
chr7	2703813	2703813	-4.7754E-02	1.44E-04	3.49E-03	TTYH3
chr14	75202689	75202689	-5.9410E-01	1.44E-04	3.49E-03	FCF1
chr4	73922610	73922610	4.9394E-01	1.45E-04	3.52E-03	COX18
chr12	44181630	44181630	3.7280E-01	1.45E-04	3.52E-03	IRAK4
chr14	92630197	92630197	9.0722E-01	1.46E-04	3.52E-03	CPSF2
chr4	4237921	4237921	2.8200E+00	1.48E-04	3.56E-03	TMEM128
chr1	204373507	204373507	8.8536E-02	1.48E-04	3.56E-03	PPP1R15B
chr10	89619513	89619513	6.1566E-01	1.48E-04	3.56E-03	KLLN
chr7	102277742	102277742	1.9485E-01	1.50E-04	3.62E-03	POLR2J2
chr5	174954969	174954969	3.6731E-01	1.51E-04	3.62E-03	SFXN1
chr4	73922598	73922598	2.8856E-01	1.51E-04	3.62E-03	COX18
chr14	92630199	92630199	7.3229E-01	1.52E-04	3.64E-03	CPSF2
chr15	41573168	41573168	7.0601E-01	1.54E-04	3.68E-03	CHP1
chr1	26161012	26161012	1.6297E+00	1.54E-04	3.68E-03	AUNIP
chrX	46407027	46407027	5.5795E-01	1.55E-04	3.69E-03	ZNF674-AS1
chr12	56215518	56215518	1.1843E+00	1.55E-04	3.69E-03	DNAJC14
chr6	18130380	18130380	2.6539E-01	1.58E-04	3.76E-03	TPMT
chr12	44181624	44181624	3.7110E-01	1.59E-04	3.79E-03	IRAK4
chr1	3806132	3806132	8.8274E-01	1.60E-04	3.81E-03	C1orf174
chr10	131978479	131978479	1.6055E+00	1.62E-04	3.84E-03	GLRX3
chr1	38490374	38490374	1.0491E+00	1.63E-04	3.86E-03	UTP11
chr19	48984889	48984889	7.5051E-01	1.64E-04	3.89E-03	CYTH2
chr1	158299328	158299328	3.8843E-01	1.65E-04	3.90E-03	CD1B
chr20	49577437	49577437	8.6929E-01	1.66E-04	3.92E-03	MOCS3
chr8	104412010	104412010	4.1198E-01	1.71E-04	4.04E-03	SLC25A32
chr19	34892829	34892829	4.6464E-01	1.73E-04	4.07E-03	GPI
chr16	66977845	66977845	-2.1346E+00	1.73E-04	4.08E-03	CES2
chr1	9788149	9788149	4.2052E-01	1.75E-04	4.12E-03	PIK3CD
chr5	36183528	36183528	1.1114E+00	1.76E-04	4.12E-03	SKP2
chr1	38490367	38490367	1.1122E+00	1.76E-04	4.12E-03	UTP11
chr14	92630189	92630189	4.6245E-01	1.78E-04	4.17E-03	CPSF2
chr19	34892852	34892852	4.7129E-01	1.79E-04	4.18E-03	GPI
chr15	41573167	41573167	6.3355E-01	1.79E-04	4.18E-03	CHP1
chr22	36623226	36623226	4.8184E-01	1.80E-04	4.20E-03	APOL2
chr1	26160981	26160981	6.6968E-01	1.82E-04	4.23E-03	AUNIP
chr16	75600306	75600306	6.5556E-01	1.82E-04	4.23E-03	GABARAPL2
chr1	26160974	26160974	6.7986E-01	1.84E-04	4.27E-03	AUNIP

chr6	18130377	18130377	4.4914E-01	1.84E-04	4.27E-03	TPMT
chr10	105885664	105885664	5.2551E-01	1.86E-04	4.31E-03	SFR1
chr11	65180580	65180580	5.9620E-01	1.91E-04	4.41E-03	FRMD8
chr4	73922611	73922611	7.8350E-01	1.93E-04	4.44E-03	COX18
chr1	26161003	26161003	1.5949E+00	1.94E-04	4.46E-03	AUNIP
chr5	10435949	10435949	1.1686E+00	1.95E-04	4.49E-03	MARCH6
chr3	48955492	48955492	1.5866E+00	1.96E-04	4.49E-03	ARIH2OS
chr19	34892828	34892828	4.6038E-01	1.96E-04	4.49E-03	GPI
chr22	24937504	24937504	5.8769E-01	1.99E-04	4.55E-03	GUCD1
chr17	76203484	76203484	6.0336E-01	2.00E-04	4.59E-03	AFMID
chr11	27516918	27516918	7.4493E-01	2.02E-04	4.62E-03	LIN7C
chr11	6505365	6505365	7.8524E-01	2.06E-04	4.70E-03	TIMM10B
chr13	38936689	38936689	3.5286E-01	2.12E-04	4.83E-03	UFM1
chr17	43002551	43002551	3.2270E-01	2.12E-04	4.83E-03	KIF18B
chr3	156258121	156258121	6.1811E-01	2.12E-04	4.83E-03	SSR3
chr1	26160977	26160977	9.5678E-01	2.13E-04	4.83E-03	AUNIP
chr16	8947917	8947917	1.6454E+00	2.16E-04	4.89E-03	CARHSP1
chr11	747455	747455	1.7391E+00	2.16E-04	4.90E-03	TALDO1
chr6	28090891	28090891	1.8325E+00	2.17E-04	4.91E-03	ZSCAN16-AS1
chr11	214977	214977	Inf	2.18E-04	4.92E-03	RIC8A
chr22	30093680	30093680	6.7696E-01	2.19E-04	4.93E-03	NF2
chr3	57306781	57306781	5.9386E-01	2.20E-04	4.95E-03	APPL1
chr3	141664024	141664024	1.7412E-01	2.20E-04	4.95E-03	TFDP2
chr1	26160971	26160971	9.7893E-01	2.21E-04	4.96E-03	AUNIP
chr9	139622572	139622572	1.2426E+00	2.21E-04	4.96E-03	SNHG7
chr12	7085779	7085779	8.5316E-01	2.25E-04	5.04E-03	LPCAT3
chr4	1813581	1813581	5.4365E-01	2.26E-04	5.06E-03	LETM1
chr17	76203502	76203502	6.2899E-01	2.27E-04	5.06E-03	AFMID
chr11	65542158	65542158	1.0293E+00	2.27E-04	5.07E-03	AP5B1
chr3	155480808	155480808	2.0903E+00	2.28E-04	5.08E-03	C3orf33
chr17	76203509	76203509	5.3533E-01	2.32E-04	5.17E-03	AFMID
chr5	140050159	140050159	#NAME?	2.37E-04	5.26E-03	WDR55
chr12	109887429	109887429	7.6822E-01	2.37E-04	5.26E-03	KCTD10
chr16	2508376	2508376	2.8195E-01	2.39E-04	5.29E-03	CCNF
chr1	26160966	26160966	1.5558E+00	2.39E-04	5.29E-03	AUNIP
chr1	26160967	26160967	1.5558E+00	2.39E-04	5.29E-03	AUNIP
chr19	1038680	1038680	1.1736E+00	2.41E-04	5.32E-03	CNN2
chr8	77894943	77894943	1.5660E+00	2.42E-04	5.35E-03	PEX2
chr9	135251188	135251188	-2.4216E-01	2.44E-04	5.37E-03	TTF1
chr9	123522777	123522777	8.0544E-01	2.47E-04	5.43E-03	FBXW2
chr17	5393303	5393303	8.0831E-01	2.48E-04	5.45E-03	MIS12

chr1	220232118	220232118	9.3118E-02	2.49E-04	5.48E-03	BPNT1
chr3	20211785	20211785	2.4345E-01	2.50E-04	5.48E-03	SGO1
chr3	57546193	57546193	1.8267E+00	2.52E-04	5.52E-03	PDE12
chr20	35280709	35280709	2.1326E-01	2.54E-04	5.54E-03	NDRG3
chr2	202151458	202151458	3.6483E-01	2.53E-04	5.54E-03	CASP8
chr16	50139149	50139149	1.4519E-01	2.54E-04	5.54E-03	HEATR3
chr4	77080741	77080741	Inf	2.57E-04	5.60E-03	SCARB2
chr16	50139150	50139150	1.7966E-01	2.60E-04	5.65E-03	HEATR3
chr19	37002417	37002417	7.3464E-01	2.60E-04	5.65E-03	ZNF260
chr9	116169947	116169947	6.8618E-01	2.63E-04	5.71E-03	POLE3
chr10	131978458	131978458	2.3435E+00	2.64E-04	5.72E-03	GLRX3
chr19	59093900	59093900	4.9039E-01	2.66E-04	5.76E-03	CENPBD1P1
chr7	138745359	138745359	Inf	2.68E-04	5.79E-03	ZC3HAV1
chr1	115312933	115312933	7.3355E-01	2.68E-04	5.79E-03	SIKE1
chr11	72540472	72540472	1.2637E-01	2.69E-04	5.79E-03	ATG16L2
chrX	46407034	46407034	4.0044E-01	2.72E-04	5.86E-03	ZNF674-AS1
chr12	44181625	44181625	3.8650E-01	2.77E-04	5.96E-03	IRAK4
chr12	56390373	56390373	1.8124E+00	2.77E-04	5.96E-03	RAB5B
chr1	9788151	9788151	3.9839E-01	2.81E-04	6.03E-03	PIK3CD
chr3	57306674	57306674	3.8856E-01	2.83E-04	6.06E-03	APPL1
chr16	29754192	29754192	Inf	2.82E-04	6.06E-03	C16orf54
chr11	65542164	65542164	1.0674E+00	2.84E-04	6.08E-03	AP5B1
chr2	197000366	197000366	1.1166E+00	2.87E-04	6.14E-03	STK17B
chr14	92630225	92630225	7.5007E-01	2.89E-04	6.18E-03	CPSF2
chr20	43995819	43995819	7.8532E-01	2.91E-04	6.20E-03	SYS1
chr1	3697447	3697447	7.2533E-01	2.91E-04	6.21E-03	LRRC47
chr11	46699404	46699404	4.6181E-01	2.92E-04	6.21E-03	ARHGAP1
chr8	28048147	28048147	9.1629E-01	2.94E-04	6.24E-03	ELP3
chr16	50139165	50139165	2.7493E-01	2.94E-04	6.25E-03	HEATR3
chr19	21907824	21907824	4.7008E-01	2.95E-04	6.26E-03	ZNF100
chr1	28661808	28661808	6.3463E-02	2.97E-04	6.29E-03	MED18
chr12	133298956	133298956	2.2005E-01	2.97E-04	6.30E-03	PGAM5
chr7	2703743	2703743	-4.9509E-02	2.99E-04	6.31E-03	TTYH3
chr14	92630226	92630226	8.4239E-01	2.98E-04	6.31E-03	CPSF2
chr4	1813586	1813586	5.5904E-01	3.02E-04	6.38E-03	LETM1
chr14	92630185	92630185	6.9468E-01	3.06E-04	6.45E-03	CPSF2
chr4	1813587	1813587	5.8039E-01	3.08E-04	6.47E-03	LETM1
chr1	204373506	204373506	8.3121E-02	3.07E-04	6.47E-03	PPP1R15B
chr17	5393297	5393297	7.9513E-01	3.10E-04	6.52E-03	MIS12
chr15	41573166	41573166	6.8802E-01	3.12E-04	6.54E-03	CHP1
chr15	32930523	32930523	3.3621E-01	3.15E-04	6.61E-03	ARHGAP11A

chr11	65542145	65542145	2.1111E+00	3.20E-04	6.68E-03	AP5B1
chr1	204392470	204392470	4.7176E-01	3.20E-04	6.68E-03	PIK3C2B
chr9	123522770	123522770	8.9035E-01	3.24E-04	6.77E-03	FBXW2
chr16	29818417	29818417	3.6336E-02	3.33E-04	6.95E-03	MAZ
chr9	116169917	116169917	1.7980E+00	3.34E-04	6.96E-03	POLE3
chr16	29818427	29818427	6.3386E-02	3.38E-04	7.03E-03	MAZ
chr10	105885640	105885640	4.7853E-01	3.38E-04	7.03E-03	SFR1
chr2	120978203	120978203	5.4202E-01	3.41E-04	7.07E-03	TMEM185B
chr3	141464950	141464950	3.5354E-01	3.51E-04	7.28E-03	RNF7
chr7	138745355	138745355	1.9705E+00	3.52E-04	7.29E-03	ZC3HAV1
chr7	44620447	44620447	7.9750E-01	3.57E-04	7.37E-03	TMED4
chr18	23596658	23596658	1.5764E+00	3.56E-04	7.37E-03	SS18
chr16	87426007	87426007	1.3180E+00	3.60E-04	7.43E-03	MAP1LC3B
chr12	72179801	72179801	3.0204E-01	3.61E-04	7.44E-03	RAB21
chr20	417651	417651	5.4210E-01	3.62E-04	7.45E-03	TBC1D20
chr3	57546175	57546175	1.6567E+00	3.63E-04	7.47E-03	PDE12
chr16	2508367	2508367	2.6005E-01	3.67E-04	7.54E-03	CCNF
chr9	123522773	123522773	6.3429E-01	3.69E-04	7.59E-03	FBXW2
chrX	46407064	46407064	1.1299E+00	3.76E-04	7.71E-03	ZNF674-AS1
chr20	35280711	35280711	2.1002E-01	3.76E-04	7.72E-03	NDRG3
chr22	24937461	24937461	6.8289E-01	3.80E-04	7.78E-03	GUCD1
chr19	34892392	34892392	1.1503E+00	3.86E-04	7.88E-03	GPI
chr12	57648178	57648178	6.3964E-01	3.86E-04	7.88E-03	R3HDM2
chrX	46741017	46741017	1.1455E+00	3.86E-04	7.88E-03	RP2
chr3	33184305	33184305	7.9755E-01	4.00E-04	8.14E-03	CRTAP
chr19	36032240	36032240	5.6858E-01	4.09E-04	8.32E-03	TMEM147-AS1
chr17	20904229	20904229	5.4940E-01	4.09E-04	8.32E-03	USP22
chr2	202151399	202151399	6.4658E-01	4.14E-04	8.39E-03	CASP8
chr19	17453035	17453035	3.9575E-01	4.14E-04	8.39E-03	GTPBP3
chr15	58245807	58245807	2.5052E+00	4.22E-04	8.54E-03	ALDH1A2
chr19	36032258	36032258	8.5739E-01	4.25E-04	8.60E-03	TMEM147-AS1
chr19	14072168	14072168	-7.3898E-02	4.26E-04	8.62E-03	DCAF15
chr16	70284458	70284458	7.4134E-01	4.27E-04	8.62E-03	EXOSC6
chr12	57648143	57648143	8.4999E-01	4.27E-04	8.62E-03	R3HDM2
chr16	2508373	2508373	2.6536E-01	4.31E-04	8.69E-03	CCNF
chrX	24095340	24095340	5.7626E-01	4.34E-04	8.74E-03	EIF2S3
chr4	166263656	166263656	5.0307E-01	4.35E-04	8.75E-03	MSMO1
chr10	105885668	105885668	4.4253E-01	4.36E-04	8.75E-03	SFR1
chr14	23517106	23517106	#NAME?	4.39E-04	8.81E-03	CDH24
chr17	5393304	5393304	6.5104E-01	4.42E-04	8.85E-03	MIS12
chr3	10193717	10193717	-4.0927E-01	4.42E-04	8.85E-03	VHL

chr3	20211754	20211754	8.3384E-01	4.44E-04	8.88E-03	SGO1
chr3	57306668	57306668	5.1162E-01	4.53E-04	9.04E-03	APPL1
chr12	7085788	7085788	8.2231E-01	4.55E-04	9.07E-03	LPCAT3
chr12	7085786	7085786	8.2744E-01	4.56E-04	9.09E-03	LPCAT3
chr16	21191685	21191685	8.3674E-01	4.57E-04	9.10E-03	TMEM159
chr15	41573117	41573117	6.8088E-01	4.58E-04	9.10E-03	CHP1
chr5	10435979	10435979	Inf	4.63E-04	9.20E-03	MARCH6
chr14	92630182	92630182	5.6741E-01	4.65E-04	9.24E-03	CPSF2
chrX	46407057	46407057	7.6544E-01	4.70E-04	9.31E-03	ZNF674-AS1
chr10	105885637	105885637	4.6659E-01	4.74E-04	9.38E-03	SFR1
chr16	2508369	2508369	2.9499E-01	4.77E-04	9.43E-03	CCNF
chr22	36623225	36623225	5.2775E-01	4.80E-04	9.48E-03	APOL2
chr4	1813560	1813560	6.8784E-01	4.81E-04	9.49E-03	LETM1
chr7	102277708	102277708	4.4966E-01	4.82E-04	9.49E-03	POLR2J2
chr5	10435966	10435966	Inf	4.82E-04	9.49E-03	MARCH6
chr16	21191698	21191698	4.7449E-01	4.86E-04	9.56E-03	TMEM159
chr19	18979588	18979588	#NAME?	4.90E-04	9.62E-03	GDF1
chr19	1038734	1038734	1.3973E+00	4.91E-04	9.63E-03	CNN2
chr4	77080742	77080742	Inf	5.04E-04	9.87E-03	SCARB2
chr15	32930520	32930520	4.0605E-01	5.05E-04	9.88E-03	ARHGAP11A
chr8	22878064	22878064	1.5907E+00	5.12E-04	1.00E-02	TNFRSF10B
chr11	6505361	6505361	8.4004E-01	5.19E-04	1.01E-02	TIMM10B
chr14	92630194	92630194	7.6051E-01	5.25E-04	1.02E-02	CPSF2
chr11	46699451	46699451	7.2935E-01	5.25E-04	1.02E-02	ARHGAP1
chr11	65542149	65542149	1.0342E+00	5.34E-04	1.04E-02	AP5B1
chr1	3729122	3729122	#NAME?	5.34E-04	1.04E-02	CEP104
chr1	211918968	211918968	6.7239E-01	5.40E-04	1.05E-02	LPGAT1
chr1	204392474	204392474	4.6268E-01	5.41E-04	1.05E-02	PIK3C2B
chr2	197000369	197000369	1.0793E+00	5.49E-04	1.06E-02	STK17B
chr17	43002550	43002550	3.6537E-01	5.49E-04	1.06E-02	KIF18B
chr3	141664022	141664022	1.3387E-01	5.57E-04	1.08E-02	TFDP2
chr2	202151385	202151385	4.8011E-01	5.57E-04	1.08E-02	CASP8
chr22	24937478	24937478	3.7204E-01	5.63E-04	1.09E-02	GUCD1
chr9	139978743	139978743	1.0377E+00	5.65E-04	1.09E-02	UAP1L1
chr15	58245733	58245733	2.1554E+00	5.72E-04	1.10E-02	ALDH1A2
chr4	1381022	1381022	2.2164E+00	5.73E-04	1.10E-02	UVSSA
chr19	37002430	37002430	6.2065E-01	5.84E-04	1.12E-02	ZNF260
chr17	35875696	35875696	6.8848E-01	5.89E-04	1.13E-02	SYNRG
chr7	92166481	92166481	-3.0745E-01	5.94E-04	1.14E-02	RBM48
chr3	141464939	141464939	3.3798E-01	5.94E-04	1.14E-02	RNF7
chr1	235294700	235294700	1.7755E+00	5.93E-04	1.14E-02	RBM34,ARID4B

chr10	131978465	131978465	1.5630E+00	5.95E-04	1.14E-02	GLRX3
chr4	664182	664182	-1.1023E+00	6.07E-04	1.16E-02	PDE6B
chr22	24937494	24937494	4.7597E-01	6.10E-04	1.16E-02	GUCD1
chr1	100757981	100757981	2.8833E-01	6.15E-04	1.17E-02	RTCA
chr7	138745315	138745315	1.0153E+00	6.16E-04	1.17E-02	ZC3HAV1
chr20	417654	417654	4.7961E-01	6.17E-04	1.17E-02	TBC1D20
chr12	56390375	56390375	1.1350E+00	6.26E-04	1.19E-02	RAB5B
chr1	23833875	23833875	1.6626E+00	6.27E-04	1.19E-02	E2F2
chr17	18147693	18147693	8.6331E-01	6.30E-04	1.19E-02	LLGL1
chr12	57648155	57648155	1.9281E+00	6.36E-04	1.20E-02	R3HDM2
chr3	57306669	57306669	4.0223E-01	6.41E-04	1.21E-02	APPL1
chr1	3806169	3806169	7.5336E-01	6.46E-04	1.22E-02	C1orf174
chr11	46699403	46699403	4.1173E-01	6.57E-04	1.24E-02	ARHGAP1
chr10	103606597	103606597	1.8837E+00	6.56E-04	1.24E-02	C10orf76
chr8	22878078	22878078	1.0460E+00	6.62E-04	1.24E-02	TNFRSF10B
chr14	75202683	75202683	-3.9733E-01	6.68E-04	1.26E-02	FCF1
chr15	32930547	32930547	4.3432E-01	6.73E-04	1.26E-02	ARHGAP11A
chr16	87426012	87426012	1.2795E+00	6.78E-04	1.27E-02	MAP1LC3B
chr16	87426013	87426013	1.3032E+00	6.78E-04	1.27E-02	MAP1LC3B
chr4	4237920	4237920	2.3087E+00	6.94E-04	1.30E-02	TMEM128
chr7	38417798	38417798	4.4932E-01	7.00E-04	1.31E-02	TRG-AS1
chr13	48620845	48620845	1.5186E+00	7.16E-04	1.34E-02	NUDT15
chr22	24937516	24937516	1.7244E+00	7.22E-04	1.35E-02	GUCD1
chr3	48955491	48955491	1.5496E+00	7.28E-04	1.36E-02	ARIH2OS
chr15	65295172	65295172	1.1343E+00	7.31E-04	1.36E-02	MTFMT
chr1	23833872	23833872	1.1286E+00	7.35E-04	1.37E-02	E2F2
chr10	111894356	111894356	4.6843E-01	7.38E-04	1.37E-02	ADD3
chr10	13252175	13252175	1.1868E+00	7.40E-04	1.37E-02	MCM10
chr17	20904227	20904227	6.5248E-01	7.42E-04	1.38E-02	USP22
chr1	26161009	26161009	9.5966E-01	7.42E-04	1.38E-02	AUNIP
chr19	17453032	17453032	3.3865E-01	7.46E-04	1.38E-02	GTPBP3
chr19	1038731	1038731	1.2972E+00	7.52E-04	1.39E-02	CNN2
chr16	15960951	15960951	1.6555E+00	7.51E-04	1.39E-02	FOPNL
chr1	115312922	115312922	5.9272E-01	7.56E-04	1.39E-02	SIKE1
chr3	155480846	155480846	5.1793E-01	7.58E-04	1.40E-02	C3orf33
chr22	39128851	39128851	7.1151E-01	7.59E-04	1.40E-02	GTPBP1
chr2	10270817	10270817	7.3802E-01	7.73E-04	1.42E-02	RRM2
chr7	138745349	138745349	1.3379E+00	7.77E-04	1.43E-02	ZC3HAV1
chr22	39128820	39128820	9.5834E-01	7.77E-04	1.43E-02	GTPBP1
chr4	1381012	1381012	1.4502E+00	7.90E-04	1.45E-02	UVSSA
chr3	57306670	57306670	4.3326E-01	7.89E-04	1.45E-02	APPL1

chr5	10435943	10435943	1.7870E+00	7.95E-04	1.45E-02	MARCH6
chr4	1813556	1813556	6.2462E-01	7.95E-04	1.45E-02	LETM1
chr7	92166479	92166479	-3.0690E-01	7.99E-04	1.46E-02	RBM48
chr1	204373510	204373510	9.5487E-02	8.01E-04	1.46E-02	PPP1R15B
chr1	38490365	38490365	6.1649E-01	8.03E-04	1.47E-02	UTP11
chr14	75203020	75203020	7.5660E-02	8.12E-04	1.48E-02	FCF1
chrX	46407065	46407065	8.0156E-01	8.14E-04	1.48E-02	ZNF674-AS1
chr16	75478248	75478248	7.9151E-01	8.15E-04	1.48E-02	TMEM170A
chr4	73922596	73922596	2.4420E-01	8.36E-04	1.52E-02	COX18
chr7	105097476	105097476	1.4477E+00	8.38E-04	1.52E-02	PUS7
chr22	30093678	30093678	5.5581E-01	8.41E-04	1.53E-02	NF2
chr12	72179798	72179798	3.2725E-01	8.45E-04	1.53E-02	RAB21
chr17	20904230	20904230	5.9466E-01	8.47E-04	1.53E-02	USP22
chr1	179832254	179832254	3.4099E-01	8.47E-04	1.53E-02	TOR1AIP2
chr11	46401805	46401805	1.3169E-01	8.49E-04	1.53E-02	DGKZ
chr22	36623091	36623091	1.4954E+00	8.51E-04	1.54E-02	APOL2
chr1	204392453	204392453	8.5493E-01	8.54E-04	1.54E-02	PIK3C2B
chr10	105885675	105885675	4.6097E-01	8.58E-04	1.54E-02	SFR1
chr1	115312925	115312925	4.6819E-01	8.58E-04	1.54E-02	SIKE1
chr6	20102070	20102070	1.3730E+00	8.65E-04	1.56E-02	MBOAT1
chr17	5393296	5393296	7.7573E-01	8.70E-04	1.56E-02	MIS12
chr1	179832238	179832238	5.8967E-01	8.70E-04	1.56E-02	TOR1AIP2
chr8	28048222	28048222	9.9311E-01	8.82E-04	1.58E-02	ELP3
chr1	179832259	179832259	3.9183E-01	8.85E-04	1.59E-02	TOR1AIP2
chr4	6718859	6718859	6.4184E-01	8.88E-04	1.59E-02	BLOC1S4
chr1	115312916	115312916	4.2505E-01	8.92E-04	1.59E-02	SIKE1
chr1	9788148	9788148	4.4287E-01	8.97E-04	1.60E-02	PIK3CD
chr3	10193711	10193711	-4.3963E-01	9.01E-04	1.61E-02	VHL
chr1	179832255	179832255	3.0412E-01	9.13E-04	1.63E-02	TOR1AIP2
chr12	57648148	57648148	1.0213E+00	9.21E-04	1.64E-02	R3HDM2
chr16	87426009	87426009	1.9880E+00	9.28E-04	1.65E-02	MAP1LC3B
chr5	10435969	10435969	1.3072E+00	9.40E-04	1.67E-02	MARCH6
chr5	176778564	176778564	-5.5790E-02	9.40E-04	1.67E-02	LMAN2
chr12	57648151	57648151	1.3078E+00	9.41E-04	1.67E-02	R3HDM2
chr19	17453033	17453033	3.4468E-01	9.43E-04	1.67E-02	GTPBP3
chr11	120203373	120203373	4.9790E-01	9.45E-04	1.67E-02	TMEM136
chr1	23833909	23833909	1.0512E+00	9.51E-04	1.68E-02	E2F2
chr11	65542150	65542150	1.0204E+00	9.54E-04	1.69E-02	AP5B1
chr22	30093687	30093687	5.6206E-01	9.56E-04	1.69E-02	NF2
chr19	1038730	1038730	1.2408E+00	9.61E-04	1.70E-02	CNN2
chr4	83551860	83551860	1.2766E+00	9.64E-04	1.70E-02	SCD5

chr17	76203505	76203505	7.1722E-01	9.66E-04	1.70E-02	AFMID
chr11	65542148	65542148	1.0337E+00	9.67E-04	1.70E-02	AP5B1
chr22	24937495	24937495	3.6027E-01	9.83E-04	1.73E-02	GUCD1
chr5	177631819	177631819	-3.0632E-01	9.84E-04	1.73E-02	HNRNPAB
chr19	1038729	1038729	1.1599E+00	9.90E-04	1.74E-02	CNN2
chr7	156803082	156803082	-1.4379E+00	1.01E-03	1.78E-02	MNX1
chr3	57306916	57306916	1.3061E+00	1.02E-03	1.78E-02	APPL1
chr6	20102078	20102078	1.9369E+00	1.02E-03	1.79E-02	MBOAT1
chr17	76203488	76203488	6.1196E-01	1.03E-03	1.79E-02	AFMID
chr16	50139142	50139142	1.8915E-01	1.04E-03	1.81E-02	HEATR3
chr16	29754197	29754197	Inf	1.04E-03	1.81E-02	C16orf54
chr17	43002718	43002718	4.7045E-01	1.05E-03	1.83E-02	KIF18B
chr15	32930412	32930412	2.5053E-01	1.07E-03	1.86E-02	ARHGAP11A
chr11	768267	768267	-9.1893E-01	1.07E-03	1.87E-02	GATD1
chr6	108533306	108533306	Inf	1.08E-03	1.88E-02	SNX3
chr1	179832258	179832258	4.6723E-01	1.09E-03	1.89E-02	TOR1AIP2
chr19	48984886	48984886	9.1243E-01	1.10E-03	1.90E-02	CYTH2
chr14	92629370	92629370	7.7683E-01	1.11E-03	1.92E-02	CPSF2
chr12	7085780	7085780	7.6276E-01	1.11E-03	1.93E-02	LPCAT3
chr11	65542170	65542170	1.2814E+00	1.12E-03	1.93E-02	AP5B1
chr4	89179643	89179643	5.0025E-01	1.13E-03	1.94E-02	PPM1K
chr9	123522769	123522769	5.4983E-01	1.13E-03	1.94E-02	FBXW2
chr16	69153594	69153594	-1.1795E-01	1.13E-03	1.95E-02	CHTF8
chr8	104411997	104411997	4.7767E-01	1.13E-03	1.95E-02	SLC25A32
chr3	10194431	10194431	6.5586E-02	1.15E-03	1.98E-02	VHL
chr6	28090890	28090890	1.3963E+00	1.15E-03	1.98E-02	ZSCAN16-AS1
chr17	43002791	43002791	5.6562E-01	1.17E-03	2.00E-02	KIF18B
chr21	45547952	45547952	3.1392E-01	1.17E-03	2.00E-02	PWP2
chr15	44158983	44158983	1.7166E+00	1.18E-03	2.02E-02	WDR76
chr14	92630184	92630184	5.4958E-01	1.18E-03	2.02E-02	CPSF2
chr12	133298897	133298897	8.3152E-01	1.18E-03	2.02E-02	PGAM5
chr19	17453029	17453029	4.0224E-01	1.19E-03	2.02E-02	GTPBP3
chr11	46694887	46694887	5.7471E-01	1.20E-03	2.04E-02	ATG13
chr19	45911189	45911189	6.2279E-01	1.21E-03	2.05E-02	ERCC1
chr17	5393309	5393309	6.3052E-01	1.20E-03	2.05E-02	MIS12
chr11	46699457	46699457	4.0154E-01	1.21E-03	2.05E-02	ARHGAP1
chr1	115312926	115312926	4.1322E-01	1.21E-03	2.05E-02	SIKE1
chr3	141464938	141464938	3.1690E-01	1.22E-03	2.07E-02	RNF7
chr9	123522783	123522783	7.7489E-01	1.23E-03	2.08E-02	FBXW2
chr12	56390378	56390378	7.9608E-01	1.24E-03	2.10E-02	RAB5B
chr17	5393300	5393300	6.3491E-01	1.25E-03	2.12E-02	MIS12

chr16	87426010	87426010	1.2867E+00	1.25E-03	2.12E-02	MAP1LC3B
chr19	1430258	1430258	-8.1084E-02	1.26E-03	2.12E-02	DAZAP1
chr17	20904243	20904243	6.2520E-01	1.26E-03	2.12E-02	USP22
chr3	141464943	141464943	3.5423E-01	1.26E-03	2.12E-02	RNF7
chr2	120978204	120978204	4.3808E-01	1.28E-03	2.15E-02	TMEM185B
chr12	57648179	57648179	5.2231E-01	1.28E-03	2.15E-02	R3HDM2
chr16	29818415	29818415	7.7959E-02	1.28E-03	2.15E-02	MAZ
chr8	22878074	22878074	1.7051E+00	1.29E-03	2.16E-02	TNFRSF10B
chr7	102277707	102277707	3.6719E-01	1.30E-03	2.18E-02	POLR2J2
chr22	39128857	39128857	7.3291E-01	1.30E-03	2.18E-02	GTPBP1
chr7	44620443	44620443	6.5009E-01	1.31E-03	2.19E-02	TMED4
chr11	46699465	46699465	4.3184E-01	1.32E-03	2.21E-02	ARHGAP1
chr3	133292766	133292766	3.1850E-01	1.32E-03	2.21E-02	CDV3
chr5	10435953	10435953	1.8341E+00	1.33E-03	2.22E-02	MARCH6
chr5	10435951	10435951	1.1233E+00	1.33E-03	2.22E-02	MARCH6
chr11	768843	768843	-8.6904E-01	1.34E-03	2.23E-02	GATD1
chr20	35280717	35280717	1.9752E-01	1.35E-03	2.24E-02	NDRG3
chr19	1038704	1038704	1.0733E+00	1.35E-03	2.25E-02	CNN2
chr10	15117874	15117874	2.1615E+00	1.35E-03	2.25E-02	ACBD7
chr3	10194433	10194433	5.6230E-02	1.36E-03	2.25E-02	VHL
chr3	156258123	156258123	4.7296E-01	1.37E-03	2.27E-02	SSR3
chr12	56390372	56390372	1.7459E+00	1.38E-03	2.28E-02	RAB5B
chr20	417652	417652	4.6909E-01	1.38E-03	2.28E-02	TBC1D20
chr9	117407193	117407193	1.2109E+00	1.38E-03	2.28E-02	TMEM268
chr1	26160969	26160969	9.2424E-01	1.39E-03	2.29E-02	AUNIP
chr1	26161011	26161011	6.6974E-01	1.39E-03	2.29E-02	AUNIP
chr4	664178	664178	-8.2719E-01	1.40E-03	2.30E-02	PDE6B
chr19	48984893	48984893	9.9610E-01	1.40E-03	2.30E-02	CYTH2
chr12	122216088	122216088	6.0057E-01	1.40E-03	2.30E-02	RHOF
chr4	73922569	73922569	2.4774E-01	1.41E-03	2.31E-02	COX18
chr1	204392449	204392449	7.2460E-01	1.42E-03	2.32E-02	PIK3C2B
chr22	36623094	36623094	1.2232E+00	1.47E-03	2.41E-02	APOL2
chr7	2703744	2703744	-4.3230E-02	1.48E-03	2.42E-02	TTYH3
chr15	65295195	65295195	3.0649E-01	1.48E-03	2.42E-02	MTFMT
chr7	106300874	106300874	2.1688E-01	1.50E-03	2.45E-02	CCDC71L
chr5	10435948	10435948	1.1238E+00	1.51E-03	2.46E-02	MARCH6
chr4	73922875	73922875	-6.2531E-01	1.51E-03	2.46E-02	COX18
chr16	50139147	50139147	1.1296E-01	1.51E-03	2.46E-02	HEATR3
chr1	26161001	26161001	6.7449E-01	1.51E-03	2.46E-02	AUNIP
chr1	3806028	3806028	1.3270E+00	1.54E-03	2.50E-02	C1orf174
chr15	32930551	32930551	2.7336E-01	1.56E-03	2.53E-02	ARHGAP11A

chr7	44620444	44620444	7.7678E-01	1.56E-03	2.53E-02	TMED4
chr3	10194429	10194429	6.7131E-02	1.57E-03	2.54E-02	VHL
chr16	70284465	70284465	6.6709E-01	1.57E-03	2.54E-02	EXOSC6
chr20	417650	417650	4.5621E-01	1.57E-03	2.54E-02	TBC1D20
chr17	17115893	17115893	-8.3066E-01	1.57E-03	2.54E-02	FLCN
chr19	48984901	48984901	5.7659E-01	1.57E-03	2.54E-02	CYTH2
chr19	34892489	34892489	1.6259E+00	1.58E-03	2.56E-02	GPI
chr4	83551930	83551930	8.2588E-01	1.59E-03	2.56E-02	SCD5
chrX	46741020	46741020	4.8703E-01	1.60E-03	2.57E-02	RP2
chr16	66977829	66977829	-1.3546E+00	1.60E-03	2.57E-02	CES2
chr20	34635175	34635175	1.2330E+00	1.61E-03	2.59E-02	NORAD
chr1	204392473	204392473	4.0812E-01	1.61E-03	2.59E-02	PIK3C2B
chr9	15460677	15460677	1.7237E+00	1.62E-03	2.59E-02	SNAPC3
chr15	65295199	65295199	2.5544E-01	1.65E-03	2.65E-02	MTFMT
chr8	104412004	104412004	9.8654E-01	1.66E-03	2.66E-02	SLC25A32
chr3	10194428	10194428	6.8081E-02	1.68E-03	2.69E-02	VHL
chr11	768839	768839	-1.8865E-01	1.68E-03	2.69E-02	GATD1
chr19	56164374	56164374	-1.3191E-01	1.69E-03	2.71E-02	CCDC106
chr20	25428739	25428739	2.4201E-01	1.74E-03	2.78E-02	GINS1
chr1	26161005	26161005	6.4795E-01	1.74E-03	2.78E-02	AUNIP
chr9	135251139	135251139	-4.3415E-01	1.75E-03	2.80E-02	TTF1
chr22	24937498	24937498	3.5094E-01	1.76E-03	2.80E-02	GUCD1
chr3	141664010	141664010	1.4317E-01	1.76E-03	2.80E-02	TFDP2
chr12	6875850	6875850	5.4551E-02	1.77E-03	2.82E-02	PTMS
chr6	28090882	28090882	1.5360E+00	1.83E-03	2.91E-02	ZSCAN16-AS1
chr16	66977830	66977830	-1.3303E+00	1.83E-03	2.91E-02	CES2
chr1	47682970	47682970	1.7201E+00	1.86E-03	2.94E-02	TAL1
chr7	48003367	48003367	4.3870E-01	1.86E-03	2.94E-02	HUS1
chr17	1325709	1325709	2.2291E+00	1.86E-03	2.95E-02	CRK
chr7	156803078	156803078	-1.4236E+00	1.87E-03	2.96E-02	MNX1
chr2	197000368	197000368	6.5350E-01	1.87E-03	2.96E-02	STK17B
chr1	204373508	204373508	7.7438E-02	1.89E-03	2.98E-02	PPP1R15B
chr1	115312915	115312915	5.4007E-01	1.90E-03	3.00E-02	SIKE1
chr6	149980490	149980490	Inf	1.91E-03	3.00E-02	LATS1
chr4	1381017	1381017	1.0842E+00	1.91E-03	3.01E-02	UVSSA
chr1	115312919	115312919	3.8938E-01	1.93E-03	3.03E-02	SIKE1
chr10	101370929	101370929	2.5633E+00	1.94E-03	3.06E-02	SLC25A28
chr9	36215088	36215088	3.6889E-01	1.95E-03	3.06E-02	GNE
chr12	133298957	133298957	2.2305E-01	1.96E-03	3.08E-02	PGAM5
chr2	120978201	120978201	4.2570E-01	1.96E-03	3.08E-02	TMEM185B
chr4	73922606	73922606	4.0062E-01	2.00E-03	3.12E-02	COX18

chr17	18112313	18112313	-6.6277E-02	2.00E-03	3.12E-02	ALKBH5
chr15	32930326	32930326	3.8199E-01	2.00E-03	3.13E-02	ARHGAP11A
chr10	51590548	51590548	Inf	2.00E-03	3.13E-02	NCOA4
chr9	15460675	15460675	1.6169E+00	2.02E-03	3.15E-02	SNAPC3
chr7	44620441	44620441	7.6909E-01	2.03E-03	3.16E-02	TMED4
chr1	204373499	204373499	7.1863E-02	2.03E-03	3.16E-02	PPP1R15B
chr7	92166482	92166482	-3.0025E-01	2.04E-03	3.17E-02	RBM48
chr15	65295174	65295174	2.5789E-01	2.03E-03	3.17E-02	MTFMT
chr17	43002719	43002719	5.2300E-01	2.05E-03	3.19E-02	KIF18B
chr6	20102069	20102069	1.3508E+00	2.05E-03	3.19E-02	MBOAT1
chr22	39128814	39128814	2.3102E+00	2.07E-03	3.22E-02	GTPBP1
chr16	2508366	2508366	2.7632E-01	2.11E-03	3.27E-02	CCNF
chr10	13252178	13252178	1.1591E+00	2.12E-03	3.28E-02	MCM10
chr1	109759493	109759493	8.8573E-01	2.12E-03	3.28E-02	SARS
chr2	197000379	197000379	5.7906E-01	2.13E-03	3.29E-02	STK17B
chr17	76203487	76203487	5.8199E-01	2.13E-03	3.29E-02	AFMID
chrX	46741054	46741054	1.9160E+00	2.15E-03	3.32E-02	RP2
chr5	140050164	140050164	-1.5898E+00	2.15E-03	3.32E-02	WDR55
chr19	56164372	56164372	-1.2675E-01	2.17E-03	3.34E-02	CCDC106
chr7	56078954	56078954	8.3551E-02	2.17E-03	3.35E-02	PSPH
chr6	28090884	28090884	1.3190E+00	2.19E-03	3.36E-02	ZSCAN16-AS1
chr5	176778563	176778563	-5.3550E-02	2.18E-03	3.36E-02	LMAN2
chr17	5393306	5393306	5.1502E-01	2.19E-03	3.36E-02	MIS12
chr12	57648188	57648188	4.9352E-01	2.18E-03	3.36E-02	R3HDM2
chr11	65180573	65180573	5.0347E-01	2.19E-03	3.36E-02	FRMD8
chr19	13884594	13884594	1.2527E-01	2.19E-03	3.36E-02	MRI1
chr19	17453036	17453036	3.4106E-01	2.19E-03	3.36E-02	GTPBP3
chr3	10194432	10194432	5.6174E-02	2.22E-03	3.40E-02	VHL
chr3	150480482	150480482	2.3156E-01	2.22E-03	3.40E-02	SIAH2
chr6	18130413	18130413	1.6756E-01	2.23E-03	3.40E-02	TPMT
chr22	24937572	24937572	7.0039E-01	2.23E-03	3.41E-02	GUCD1
chr4	148555998	148555998	5.7500E-01	2.25E-03	3.42E-02	TMEM184C
chr2	10270803	10270803	3.6429E-01	2.25E-03	3.43E-02	RRM2
chr22	36623122	36623122	1.4290E+00	2.26E-03	3.44E-02	APOL2
chr16	50139159	50139159	2.2527E-01	2.27E-03	3.45E-02	HEATR3
chr4	73922607	73922607	3.2008E-01	2.31E-03	3.51E-02	COX18
chr7	100210428	100210428	-3.2023E-01	2.34E-03	3.56E-02	MOSPD3
chr22	39128854	39128854	8.6454E-01	2.37E-03	3.60E-02	GTPBP1
chr17	17115886	17115886	-8.2565E-01	2.40E-03	3.64E-02	FLCN
chr16	29818430	29818430	5.4040E-02	2.42E-03	3.65E-02	MAZ
chr15	40712056	40712056	2.3126E+00	2.41E-03	3.65E-02	IVD

chr12	57648157	57648157	1.2108E+00	2.42E-03	3.65E-02	R3HDM2
chr1	115312921	115312921	4.9899E-01	2.42E-03	3.65E-02	SIKE1
chr2	197000399	197000399	8.7512E-01	2.42E-03	3.65E-02	STK17B
chr22	36623242	36623242	3.8869E-01	2.42E-03	3.65E-02	APOL2
chr17	18147602	18147602	8.7259E-01	2.44E-03	3.68E-02	LLGL1
chr14	20811510	20811510	-1.2623E-01	2.45E-03	3.69E-02	RPPH1
chr11	65180574	65180574	6.2312E-01	2.45E-03	3.69E-02	FRMD8
chr11	120203382	120203382	9.1020E-01	2.45E-03	3.69E-02	TMEM136
chr10	13252164	13252164	9.1662E-01	2.46E-03	3.70E-02	MCM10
chr1	23833906	23833906	1.0071E+00	2.48E-03	3.71E-02	E2F2
chr17	5393312	5393312	5.1141E-01	2.52E-03	3.77E-02	MIS12
chr14	75202676	75202676	-4.1949E-01	2.54E-03	3.80E-02	FCF1
chr1	109759500	109759500	8.8020E-01	2.54E-03	3.80E-02	SARS
chr9	123522779	123522779	7.6801E-01	2.55E-03	3.81E-02	FBXW2
chr4	4237871	4237871	1.5017E+00	2.56E-03	3.82E-02	TMEM128
chr3	156258117	156258117	4.5413E-01	2.56E-03	3.82E-02	SSR3
chr22	24937471	24937471	3.3012E-01	2.56E-03	3.82E-02	GUCD1
chr19	1038725	1038725	1.2184E+00	2.57E-03	3.83E-02	CNN2
chr17	5393299	5393299	5.1292E-01	2.59E-03	3.85E-02	MIS12
chr20	62371264	62371264	-2.4604E-01	2.59E-03	3.86E-02	SLC2A4RG
chr1	179832256	179832256	3.5668E-01	2.60E-03	3.87E-02	TOR1AIP2
chr16	75600307	75600307	5.2238E-01	2.61E-03	3.87E-02	GABARAPL2
chr4	1381020	1381020	1.0947E+00	2.61E-03	3.87E-02	UVSSA
chr4	1381014	1381014	8.2887E-01	2.64E-03	3.92E-02	UVSSA
chr13	48620840	48620840	1.8710E+00	2.66E-03	3.94E-02	NUDT15
chr17	17115899	17115899	-8.1673E-01	2.67E-03	3.96E-02	FLCN
chr9	36215185	36215185	-4.0536E-01	2.69E-03	3.97E-02	GNE
chr8	77894931	77894931	1.1187E+00	2.69E-03	3.98E-02	PEX2
chr12	58152524	58152524	-6.5617E-02	2.70E-03	3.98E-02	MARCH9
chr12	102139693	102139693	1.0665E+00	2.70E-03	3.98E-02	GNPTAB
chr1	109759489	109759489	1.4435E+00	2.70E-03	3.98E-02	SARS
chr19	1038722	1038722	1.3840E+00	2.70E-03	3.98E-02	CNN2
chr4	73922597	73922597	2.1218E-01	2.72E-03	4.00E-02	COX18
chr19	1038727	1038727	1.1314E+00	2.72E-03	4.00E-02	CNN2
chr10	104498170	104498170	1.1614E+00	2.73E-03	4.02E-02	SFXN2
chr11	120203379	120203379	4.7322E-01	2.76E-03	4.05E-02	TMEM136
chr2	204296301	204296301	-2.2176E+00	2.77E-03	4.06E-02	ABI2
chr19	14072173	14072173	-6.5297E-02	2.77E-03	4.06E-02	DCAF15
chr2	120978207	120978207	8.1585E-01	2.78E-03	4.06E-02	TMEM185B
chrX	13731090	13731090	8.1661E-01	2.78E-03	4.07E-02	TRAPPC2
chr8	23290130	23290130	9.5647E-01	2.79E-03	4.08E-02	ENTPD4

chr19	37002416	37002416	9.7703E-01	2.79E-03	4.08E-02	ZNF260
chr9	33045723	33045723	9.8974E-01	2.83E-03	4.11E-02	SMU1
chr20	35221342	35221342	8.8556E-01	2.82E-03	4.11E-02	TGIF2
chr2	197000400	197000400	1.0708E+00	2.83E-03	4.11E-02	STK17B
chr16	21191694	21191694	3.7522E-01	2.82E-03	4.11E-02	TMEM159
chr10	105885677	105885677	4.1107E-01	2.83E-03	4.12E-02	SFR1
chr17	35875796	35875796	4.8462E-01	2.85E-03	4.15E-02	SYNRG
chr5	10435952	10435952	1.0819E+00	2.86E-03	4.15E-02	MARCH6
chr19	37035999	37035999	1.0542E+00	2.88E-03	4.17E-02	ZNF529
chr1	115312937	115312937	6.4387E-01	2.88E-03	4.17E-02	SIKE1
chr1	23833865	23833865	1.1435E+00	2.89E-03	4.18E-02	E2F2
chr7	44620419	44620419	7.4517E-01	2.90E-03	4.20E-02	TMED4
chr10	15117880	15117880	2.1068E+00	2.91E-03	4.22E-02	ACBD7
chr22	24937560	24937560	6.8538E-01	2.92E-03	4.22E-02	GUCD1
chr3	141664019	141664019	1.2606E-01	2.94E-03	4.24E-02	TFDP2
chr6	28090881	28090881	1.3219E+00	2.97E-03	4.29E-02	ZSCAN16-AS1
chr4	159630578	159630578	8.2699E-01	3.00E-03	4.34E-02	PPID
chr19	1038675	1038675	8.5178E-01	3.01E-03	4.34E-02	CNN2
chr11	65542162	65542162	7.8488E-01	3.01E-03	4.34E-02	AP5B1
chr14	92630173	92630173	5.5708E-01	3.05E-03	4.39E-02	CPSF2
chr3	57306742	57306742	5.6145E-01	3.06E-03	4.40E-02	APPL1
chr19	14072167	14072167	-6.2842E-02	3.07E-03	4.41E-02	DCAF15
chr1	3806178	3806178	1.1313E+00	3.08E-03	4.42E-02	C1orf174
chr3	141464941	141464941	3.3808E-01	3.09E-03	4.43E-02	RNF7
chr17	43002792	43002792	5.8299E-01	3.10E-03	4.45E-02	KIF18B
chr19	1038620	1038620	6.1263E-01	3.11E-03	4.46E-02	CNN2
chr3	10193718	10193718	-3.3239E-01	3.12E-03	4.47E-02	VHL
chr9	140099920	140099920	1.6214E+00	3.13E-03	4.47E-02	TMEM203
chr2	202151461	202151461	3.0674E-01	3.13E-03	4.48E-02	CASP8
chr19	13884592	13884592	1.1758E-01	3.14E-03	4.49E-02	MRI1
chr4	1813569	1813569	1.1451E+00	3.15E-03	4.50E-02	LETM1
chr4	83551862	83551862	1.2427E+00	3.16E-03	4.51E-02	SCD5
chr13	48620850	48620850	1.1829E+00	3.17E-03	4.52E-02	NUDT15
chr3	57546339	57546339	Inf	3.18E-03	4.53E-02	PDE12
chr19	13884595	13884595	1.0856E-01	3.18E-03	4.53E-02	MRI1
chr3	141464942	141464942	2.9080E-01	3.22E-03	4.58E-02	RNF7
chr7	2703821	2703821	-7.0753E-02	3.23E-03	4.59E-02	TTYH3
chr6	28090893	28090893	1.4946E+00	3.26E-03	4.62E-02	ZSCAN16-AS1
chr9	123522804	123522804	9.7223E-01	3.28E-03	4.65E-02	FBXW2
chr15	52840582	52840582	1.0077E+00	3.32E-03	4.70E-02	ARPP19
chr17	20904232	20904232	5.5609E-01	3.32E-03	4.71E-02	USP22

chr20	35221353	35221353	8.8347E-01	3.33E-03	4.71E-02	TGIF2
chr17	17116046	17116046	-6.6979E-01	3.34E-03	4.73E-02	FLCN
chr19	21368518	21368518	2.2349E-01	3.37E-03	4.76E-02	ZNF431
chr1	3806166	3806166	5.0168E-01	3.37E-03	4.76E-02	C1orf174
chr11	66626366	66626366	9.7205E-02	3.38E-03	4.77E-02	LRFN4
chr19	14072166	14072166	-6.3645E-02	3.40E-03	4.79E-02	DCAF15
chr10	13252177	13252177	1.1399E+00	3.40E-03	4.79E-02	MCM10
chr7	135360733	135360733	9.8725E-01	3.42E-03	4.82E-02	STMP1
chr4	166263658	166263658	6.4993E-01	3.44E-03	4.84E-02	MSMO1
chr12	57648141	57648141	9.9547E-01	3.45E-03	4.86E-02	R3HDM2
chr6	28090885	28090885	1.2885E+00	3.48E-03	4.89E-02	ZSCAN16-AS1
chr11	768861	768861	-9.1631E-02	3.50E-03	4.92E-02	GATD1
chr11	124749818	124749818	1.3694E+00	3.51E-03	4.92E-02	ROBO3
chr1	3697420	3697420	8.7170E-01	3.53E-03	4.95E-02	LRRC47
chrX	46741061	46741061	1.3296E+00	3.54E-03	4.95E-02	RP2
chr14	93649251	93649251	7.4332E-01	3.54E-03	4.95E-02	MOAP1
chr22	30093679	30093679	5.3183E-01	3.55E-03	4.96E-02	NF2
chr6	28090895	28090895	1.6951E+00	3.55E-03	4.96E-02	ZSCAN16-AS1

KEGG_hyper

Category	Term	PValue	Genes	Fold Enrichment
KEGG_PATHWAY	hsa04110:Cell cycle	0.001428995	4998, 7040, 51343, 990, 7027, 7709, 1028, 7029, 6502, 1870	3.690450758
KEGG_PATHWAY	hsa05169:Epstein-Barr virus infection	0.008152694	6693, 5293, 548644, 5335, 5757, 5709, 6908, 5433, 6502, 208, 246721	2.649355176
KEGG_PATHWAY	hsa04140:Regulation of autophagy	0.009727205	89849, 9776, 55054, 11345, 11337	5.866870436
KEGG_PATHWAY	hsa05211:Renal cell carcinoma	0.013099377	1398, 7040, 5293, 2885, 7428, 208	4.224146714
KEGG_PATHWAY	hsa01130:Biosynthesis of antibiotics	0.016715894	2224, 6307, 6888, 10380, 2222, 5230, 2597, 5723, 5198, 91373, 2821	2.374422092
KEGG_PATHWAY	hsa00564:Glycerophospholipid metabolism	0.01689451	154141, 10162, 8525, 81490, 54675, 9926, 79143	3.371906588
KEGG_PATHWAY	hsa05220:Chronic myeloid leukemia	0.019679986	1398, 7040, 5293, 2885, 208, 1870	3.813465784
KEGG_PATHWAY	hsa05142:Chagas disease (American trypanosomiasis)	0.025202388	7040, 841, 5293, 2778, 2773, 51135, 208	3.080106979
KEGG_PATHWAY	hsa00240:Pyrimidine metabolism	0.025202388	548644, 54107, 5558, 6241, 5433, 9583, 246721	3.080106979
KEGG_PATHWAY	hsa01100:Metabolic pathways	0.031457004	10998, 5230, 54107, 5558, 6241, 2224, 6888, 93100, 2222, 10380,	1.378810104

			7358, 2597, 8525, 125061, 5287, 84245, 246721, 3712, 8854, 154141, 10020, 5723, 9563, 5198, 81490, 91373, 23545, 6307, 548644, 5335, 133686, 51, 5433, 54675, 4669, 2821, 54802	
KEGG_PATHWAY	hsa05223:Non-small cell lung cancer	0.032775214	5293, 5335, 2885, 208, 1870	4.085856197
KEGG_PATHWAY	hsa00230:Purine metabolism	0.037307842	548644, 5158, 54107, 5558, 6241, 5198, 5433, 9583, 246721	2.340081276
KEGG_PATHWAY	hsa04722:Neurotrophin signaling pathway	0.045938982	1398, 5293, 5335, 2885, 397, 51135, 208	2.669426049
KEGG_PATHWAY	hsa05214:Glioma	0.052126186	5293, 5335, 2885, 208, 1870	3.520122262
KEGG_PATHWAY	hsa04962:Vasopressin-regulated water reabsorption	0.069617083	5869, 397, 2778, 10540	4.160144491
KEGG_PATHWAY	hsa04068:FoxO signaling pathway	0.070898869	7040, 5293, 2885, 11345, 11337, 6502, 208	2.39053079
KEGG_PATHWAY	hsa03022:Basal transcription factors	0.073450683	6908, 6882, 129685, 2967	4.067696836
KEGG_PATHWAY	hsa05200:Pathways in cancer	0.084293638	1398, 841, 7040, 5293, 5335, 2885, 8031, 26060, 7428, 2778, 2773, 6502, 208, 1870	1.630183846
KEGG_PATHWAY	hsa00520:Amino sugar and nucleotide sugar metabolism	0.08549845	7358, 10020, 91373, 2821	3.813465784
KEGG_PATHWAY	hsa01200:Carbon metabolism	0.098740379	6888, 5230, 2597, 5723, 9563, 2821	2.429818906

KEGG_hypo

Category	Term	PValue	Genes	Fold Enrichment
KEGG_PATHWAY	hsa00520:Amino sugar and nucleotide sugar metabolism	0.062948868	64841, 10020, 3074	7.1979
KEGG_PATHWAY	hsa04144:Endocytosis	0.068255788	7040, 116988, 30011, 156, 29924, 9146	2.6783
KEGG_PATHWAY	hsa05145:Toxoplasmosis	0.079297473	7040, 331, 51135, 4793	3.9040

BP_hyper

Category	Term	PValue	Genes	Fold Enrichment
GOTERM_BP_DIRECT	GO:0015031~protein transport	4.18E-05	53916, 26515, 91056, 55054, 326624, 9545, 9907, 23011, 57132, 9094, 222068, 5481, 89849, 55327, 348995,	2.8086

			8724, 11345, 5869, 285521, 85406, 11337, 6102	
GOTERM_BP_DIRECT	GO:0006260~DNA replication	6.07E-05	10524, 84289, 84296, 672, 54107, 6241, 3364, 4998, 55388, 54921, 990, 83695, 5763	4.2293
GOTERM_BP_DIRECT	GO:0007067~mitotic nuclear division	4.37E-04	55920, 348235, 899, 51343, 9113, 348995, 55839, 990, 10776, 5536, 151648, 54461, 23137, 10540, 79003	3.0500
GOTERM_BP_DIRECT	GO:0008654~phospholipid biosynthetic process	1.09E-03	154141, 10162, 81490, 54675, 9926, 79143	7.5640
GOTERM_BP_DIRECT	GO:0000724~double-strand break repair via homologous recombination	3.42E-03	7517, 119392, 672, 7516, 9907, 3364, 23137	4.7701
GOTERM_BP_DIRECT	GO:0006270~DNA replication initiation	3.49E-03	4998, 55388, 84296, 990, 5558	7.8791
GOTERM_BP_DIRECT	GO:0000082~G1/S transition of mitotic cell cycle	4.12E-03	4998, 51379, 55388, 9113, 990, 5558, 6241, 6502	3.9550
GOTERM_BP_DIRECT	GO:0008156~negative regulation of DNA replication	5.87E-03	7040, 990, 4771, 3364	10.6161
GOTERM_BP_DIRECT	GO:0001933~negative regulation of protein phosphorylation	7.06E-03	80279, 7040, 2280, 84919, 5536, 11261	4.9600
GOTERM_BP_DIRECT	GO:0000045~autophagosome assembly	8.54E-03	81631, 89849, 9776, 55054, 11345	6.1496
GOTERM_BP_DIRECT	GO:0000278~mitotic cell cycle	8.54E-03	55270, 146909, 7027, 7516, 7029	6.1496
GOTERM_BP_DIRECT	GO:0007059~chromosome segregation	1.11E-02	348235, 9391, 672, 55839, 151648, 79003	4.4494
GOTERM_BP_DIRECT	GO:1901796~regulation of signal transduction by p53 class mediator	1.17E-02	10524, 84289, 672, 6908, 6882, 83695, 3364, 79813	3.2533
GOTERM_BP_DIRECT	GO:0042795~snRNA transcription from RNA polymerase II promoter	1.24E-02	6619, 57508, 6908, 6882, 5433, 129685	4.3223
GOTERM_BP_DIRECT	GO:0002098~tRNA wobble uridine modification	1.28E-02	27304, 84705, 55140	16.8088
GOTERM_BP_DIRECT	GO:0070475~rRNA base methylation	1.28E-02	91893, 155400, 260294	16.8088
GOTERM_BP_DIRECT	GO:0071260~cellular response to mechanical stimulus	1.32E-02	7040, 841, 22927, 8795, 1265, 835	4.2614
GOTERM_BP_DIRECT	GO:0000732~strand displacement	1.42E-02	7517, 10524, 672, 7516	7.7579
GOTERM_BP_DIRECT	GO:0050765~negative regulation of phagocytosis	1.58E-02	7040, 8724, 1265	15.1279
GOTERM_BP_DIRECT	GO:0010212~response to ionizing radiation	1.58E-02	7517, 10524, 672, 7516, 84446	5.1456
GOTERM_BP_DIRECT	GO:0043065~positive regulation of apoptotic process	1.63E-02	8854, 84289, 7040, 5481, 64112, 9113, 9148, 23385, 51118, 64857, 90427, 10687, 835	2.1851
GOTERM_BP_DIRECT	GO:0007062~sister chromatid cohesion	1.65E-02	55920, 348235, 348995, 55839, 151648, 79003, 23137	3.4270

GOTERM_BP_DIRECT	GO:0016236~macroautophagy	1.73E-02	81631, 9776, 55054, 11345, 10670, 11337	3.9810
GOTERM_BP_DIRECT	GO:0051301~cell division	2.25E-02	55920, 146909, 2773, 151648, 57132, 348235, 899, 51343, 348995, 9113, 990, 10776, 79003, 23137	2.0171
GOTERM_BP_DIRECT	GO:0061014~positive regulation of mRNA catabolic process	2.26E-02	10236, 56829, 9567	12.6066
GOTERM_BP_DIRECT	GO:0001833~inner cell mass cell proliferation	2.26E-02	84296, 9837, 129685	12.6066
GOTERM_BP_DIRECT	GO:0071479~cellular response to ionizing radiation	2.29E-02	7040, 2885, 83695, 3364	6.5066
GOTERM_BP_DIRECT	GO:0032781~positive regulation of ATPase activity	2.29E-02	11345, 3337, 163590, 130872	6.5066
GOTERM_BP_DIRECT	GO:0006312~mitotic recombination	2.63E-02	2067, 7516, 7155	11.6369
GOTERM_BP_DIRECT	GO:0070306~lens fiber cell differentiation	2.63E-02	7040, 51343, 4771	11.6369
GOTERM_BP_DIRECT	GO:0006364~rRNA processing	2.69E-02	6228, 5822, 79707, 6143, 51077, 3921, 118460, 51118, 54853, 6187	2.3564
GOTERM_BP_DIRECT	GO:0000038~very long-chain fatty acid metabolic process	3.03E-02	10998, 51, 5828	10.8057
GOTERM_BP_DIRECT	GO:0000731~DNA synthesis involved in DNA repair	3.14E-02	7517, 10524, 672, 7516	5.7630
GOTERM_BP_DIRECT	GO:0006271~DNA strand elongation involved in DNA replication	3.45E-02	84296, 9837, 5558	10.0853
GOTERM_BP_DIRECT	GO:0008033~tRNA processing	3.63E-02	54517, 6301, 91893, 54802	5.4515
GOTERM_BP_DIRECT	GO:0006978~DNA damage response, signal transduction by p53 class mediator resulting in transcription of p21 class mediator	3.90E-02	10524, 672, 4582	9.4550
GOTERM_BP_DIRECT	GO:0039689~negative stranded viral RNA replication	3.92E-02	89849, 55054	50.4264
GOTERM_BP_DIRECT	GO:0010824~regulation of centrosome duplication	5.34E-02	7517, 57132, 54461	7.9621
GOTERM_BP_DIRECT	GO:0006413~translational initiation	5.48E-02	123263, 6228, 8668, 6143, 1968, 3921, 6187	2.5765
GOTERM_BP_DIRECT	GO:0006094~gluconeogenesis	5.60E-02	5230, 2597, 1407, 2821	4.5842
GOTERM_BP_DIRECT	GO:0000819~sister chromatid segregation	5.82E-02	9113, 7155	33.6176
GOTERM_BP_DIRECT	GO:0031572~G2 DNA damage checkpoint	5.86E-02	51343, 672, 84446	7.5640
GOTERM_BP_DIRECT	GO:0006283~transcription-coupled nucleotide-excision repair	5.88E-02	57461, 2067, 57654, 5433, 2967	3.4072
GOTERM_BP_DIRECT	GO:0006412~translation	6.52E-02	6228, 399512, 81034, 6301, 6143, 64960, 81894, 3921, 2967, 6187	1.9931
GOTERM_BP_DIRECT	GO:0035264~multicellular organism growth	7.39E-02	2067, 7516, 59338, 1028, 2778	3.1517
GOTERM_BP_DIRECT	GO:0001731~formation of translation preinitiation	7.51E-02	8668, 8562, 1968	6.5774

	complex			
GOTERM_BP_DIRECT	GO:0000083~regulation of transcription involved in G1/S transition of mitotic cell cycle	7.51E-02	4998, 990, 6241	6.5774
GOTERM_BP_DIRECT	GO:0010716~negative regulation of extracellular matrix disassembly	7.68E-02	7040, 1471	25.2132
GOTERM_BP_DIRECT	GO:0071514~genetic imprinting	7.68E-02	1028, 2778	25.2132
GOTERM_BP_DIRECT	GO:0051280~negative regulation of release of sequestered calcium ion into cytosol	7.68E-02	7040, 2280	25.2132
GOTERM_BP_DIRECT	GO:0000707~meiotic DNA recombinase assembly	7.68E-02	7517, 7516	25.2132
GOTERM_BP_DIRECT	GO:0051984~positive regulation of chromosome segregation	7.68E-02	990, 23137	25.2132
GOTERM_BP_DIRECT	GO:0031146~SCF-dependent proteasomal ubiquitin-dependent protein catabolic process	8.09E-02	899, 9616, 54461	6.3033
GOTERM_BP_DIRECT	GO:0006887~exocytosis	8.21E-02	3996, 55327, 84958, 9545, 51248	3.0377
GOTERM_BP_DIRECT	GO:0071158~positive regulation of cell cycle arrest	8.68E-02	7040, 51379, 672	6.0512
GOTERM_BP_DIRECT	GO:0098609~cell-cell adhesion	9.02E-02	57142, 392, 1968, 5093, 56829, 3337, 64837, 8496, 6187, 1265	1.8608
GOTERM_BP_DIRECT	GO:0006368~transcription elongation from RNA polymerase II promoter	9.08E-02	6908, 6882, 5433, 55140, 2967	2.9318
GOTERM_BP_DIRECT	GO:0061621~canonical glycolysis	9.29E-02	5230, 2597, 2821	5.8184
GOTERM_BP_DIRECT	GO:0017157~regulation of exocytosis	9.29E-02	3996, 9545, 23011	5.8184
GOTERM_BP_DIRECT	GO:0006432~phenylalanyl-tRNA aminoacylation	9.50E-02	57470, 91893	20.1706
GOTERM_BP_DIRECT	GO:1903361~protein localization to basolateral plasma membrane	9.50E-02	55327, 51248	20.1706
GOTERM_BP_DIRECT	GO:0002192~IRES-dependent translational initiation	9.50E-02	8562, 5936	20.1706
GOTERM_BP_DIRECT	GO:0071569~protein ufmylation	9.50E-02	80279, 51569	20.1706
GOTERM_BP_DIRECT	GO:0090656~t-circle formation	9.50E-02	7517, 2067	20.1706
GOTERM_BP_DIRECT	GO:1903874~ferrous iron transmembrane transport	9.50E-02	4891, 81894	20.1706
GOTERM_BP_DIRECT	GO:0006281~DNA repair	9.55E-02	7517, 2067, 51343, 672, 7516, 2177, 5536, 3364, 2967	1.9312

BP_hypo

Category	Term	PValue	Genes	Fold Enrichment
GOTERM_BP_DIRECT	GO:0042059~negative regulation of epidermal growth factor receptor signaling pathway	3.25E-03	55658, 30011, 29924, 9146	13.3270
GOTERM_BP_DIRECT	GO:0070936~protein K48-linked ubiquitination	6.90E-03	54926, 55658, 7326, 84937	10.2079
GOTERM_BP_DIRECT	GO:0016477~cell migration	1.42E-02	10152, 7040, 399687, 146206, 30011, 983	4.1841
GOTERM_BP_DIRECT	GO:0030511~positive regulation of transforming growth factor beta receptor signaling pathway	1.67E-02	201163, 4221, 3622	14.9929
GOTERM_BP_DIRECT	GO:0018105~peptidyl-serine phosphorylation	2.02E-02	1455, 5566, 5585, 156, 983	4.7977
GOTERM_BP_DIRECT	GO:0000077~DNA damage checkpoint	2.54E-02	1869, 3014, 8682	11.9943
GOTERM_BP_DIRECT	GO:0043161~proteasome-mediated ubiquitin-dependent protein catabolic process	2.69E-02	54926, 55658, 84937, 153830, 81858, 983	3.5451
GOTERM_BP_DIRECT	GO:0006363~termination of RNA polymerase I transcription	2.70E-02	5434, 7270, 2967	11.6074
GOTERM_BP_DIRECT	GO:0006361~transcription initiation from RNA polymerase I promoter	3.04E-02	5434, 7270, 2967	10.9039
GOTERM_BP_DIRECT	GO:0006383~transcription from RNA polymerase III promoter	3.04E-02	27297, 5434, 6621	10.9039
GOTERM_BP_DIRECT	GO:0018107~peptidyl-threonine phosphorylation	3.94E-02	5566, 156, 983	9.4692
GOTERM_BP_DIRECT	GO:0045599~negative regulation of fat cell differentiation	4.72E-02	7040, 1869, 161882	8.5673
GOTERM_BP_DIRECT	GO:0045591~positive regulation of regulatory T cell differentiation	4.87E-02	7040, 146206	39.9810
GOTERM_BP_DIRECT	GO:0006412~translation	5.94E-02	1468, 79751, 64146, 65003, 6139, 2967	2.8445
GOTERM_BP_DIRECT	GO:0010628~positive regulation of gene expression	6.68E-02	8880, 7040, 1869, 9146, 983, 1265	2.7468
GOTERM_BP_DIRECT	GO:0042127~regulation of cell proliferation	6.78E-02	55658, 331, 8764, 3727, 1265	3.2417
GOTERM_BP_DIRECT	GO:0050765~negative regulation of phagocytosis	7.98E-02	7040, 1265	23.9886
GOTERM_BP_DIRECT	GO:0008535~respiratory chain complex IV assembly	7.98E-02	1353, 285521	23.9886
GOTERM_BP_DIRECT	GO:0030097~hemopoiesis	8.58E-02	861, 201163, 1265	6.0988
GOTERM_BP_DIRECT	GO:0031065~positive regulation of histone deacetylation	8.74E-02	7040, 3622	21.8078
GOTERM_BP_DIRECT	GO:0030851~granulocyte differentiation	8.74E-02	863, 161882	21.8078
GOTERM_BP_DIRECT	GO:0050727~regulation of inflammatory response	9.59E-02	22904, 7791, 331	5.7116

GOTERM_BP_DIRECT	GO:0006915~apoptotic process	9.89E-02	9220, 7818, 84971, 9262, 331, 90525, 30011, 983, 8682	1.9039
GOTERM_BP_DIRECT	GO:0006914~autophagy	9.92E-02	410, 84971, 79142, 9146	3.6073
